# Improved Antibacterial Activity of 1,3,4-Oxadiazole-Based Compounds That Restrict *Staphylococcus aureus* Growth Independent of LtaS Function

**DOI:** 10.1021/acsinfecdis.3c00250

**Published:** 2023-10-13

**Authors:** Edward J. A. Douglas, Brandon Marshall, Arwa Alghamadi, Erin A. Joseph, Seána Duggan, Serena Vittorio, Laura De Luca, Michaela Serpi, Maisem Laabei

**Affiliations:** † Department of Life Sciences, University of Bath, Bath BA2 7AY, U.K.; ‡ School of Chemistry, Cardiff University, Cardiff CF10 3AT, Wales, U.K.; § Medical Research Council Centre for Medical Mycology at the University of Exeter, University of Exeter, Exeter EX4 4DQ, U.K.; ∥ Department of Chemical, Biological, Pharmaceutical and Environmental Sciences, University of Messina, Messina I-98125, Italy

**Keywords:** Staphylococcus aureus, lipoteichoic acid inhibitors, antimicrobial resistance, drug discovery, 1,3,4 oxadiazole

## Abstract

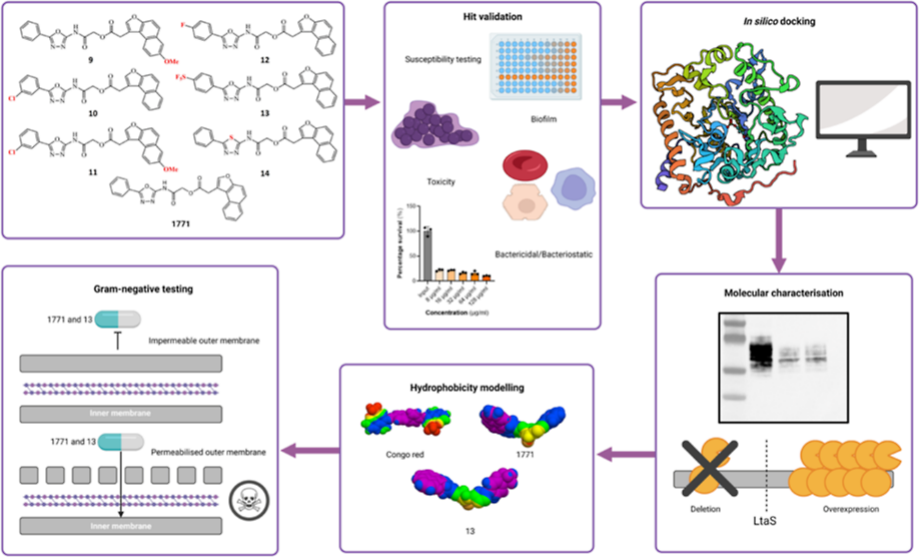

The lipoteichoic acid (LTA) biosynthesis pathway has emerged as a promising antimicrobial therapeutic target. Previous studies identified the 1,3,4 oxadiazole compound 1771 as an LTA inhibitor with activity against Gram-positive pathogens. We have succeeded in making six 1771 derivatives and, through subsequent hit validation, identified the incorporation of a pentafluorosulfanyl substituent as central in enhancing activity. Our newly described derivative, compound **13**, showed a 16- to 32-fold increase in activity compared to 1771 when tested against a cohort of multidrug-resistant *Staphylococcus aureus* strains while simultaneously exhibiting an improved toxicity profile against mammalian cells. Molecular techniques were employed in which the assumed target, lipoteichoic acid synthase (LtaS), was both deleted and overexpressed. Neither deletion nor overexpression of LtaS altered 1771 or compound **13** susceptibility; however, overexpression of LtaS increased the MIC of Congo red, a previously identified LtaS inhibitor. These data were further supported by comparing the docking poses of 1771 and derivatives in the LtaS active site, which indicated the possibility of an additional target(s). Finally, we show that both 1771 and compound **13** have activity that is independent of LtaS, extending to cover Gram-negative species if the outer membrane is first permeabilized, challenging the classification that these compounds are strict LtaS inhibitors.

*Staphylococcus aureus* is a major human pathogen, responsible for a broad spectrum of illnesses ranging from minor skin disease to life-threating systemic infections and toxinoses.^1^ In a recent global systematic analysis of antimicrobial burden, *S. aureus* was identified as one of the top six pathogens responsible for deaths associated with and attributed to antimicrobial resistance. Moreover, this analysis indicated that methicillin-resistant *S. aureus* (MRSA) was responsible for over 100,000 deaths worldwide in 2019.^2^

Vancomycin (glycopeptide), daptomycin (lipopeptide), and linezolid (oxazolidinone) remain the treatments of choice for severe MRSA infections.^1^ However, along with poor tissue penetration and relatively slow bactericidal activity, there is growing concern over the decreasing susceptibility of MRSA to vancomycin.^3,4^ Similarly, resistance toward daptomycin primarily due to mutations in the bacterial phospholipid synthase and flippase gene *mprF*(5) and toward linezolid due to ribosomal mutations^6^ and the acquisition of *cfr* (chloramphenicol-florfenicol resistance) methyltransferase^7^ are increasingly reported. Therefore, more concerted efforts are required to identify alternative Gram-positive and or specific *S. aureus* targets for the design of therapeutic compounds, which we have recently reviewed.^8^

Teichoic acids are glycopolymeric structures that form an integral part of the Gram-positive cell envelope and can either be attached to peptidoglycan as wall teichoic acids or anchored to membrane lipids as lipoteichoic acids (LTAs).^9,10^ Both structures have been shown to be important for *S. aureus* growth and virulence and therefore are considered attractive druggable targets.^9,10^ Recent studies have reported success in targeting LTA biosynthesis.^11−14^ LTA consists of a polyglycerophosphate chain, which is covalently linked to a diglucosyl-diacylglycerol (Glc_2_-DAG) anchor in the membrane. Five types of LTAs exist in bacteria with *S. aureus* producing type I LTA, which consists of polyglycerol units that are joined through phosphodiester linkages and further coated with d-alanine or carbohydrate residues.^15^

The structural and enzymatic machinery involved in the synthesis of *S. aureus* LTA has been elucidated (Figure 1), and findings center around the function of LtaS, which catalyzes the formation of the poly glycerol phosphate backbone.^15^ The LTA pathway contains several crucial enzymes that are confirmed to be either essential (PgsA, DgkB, and CdsA)^16−18^ or conditionally essential (LtaS),^19^ making them potential drug targets for inhibiting LTA synthesis.

**Figure 1:**
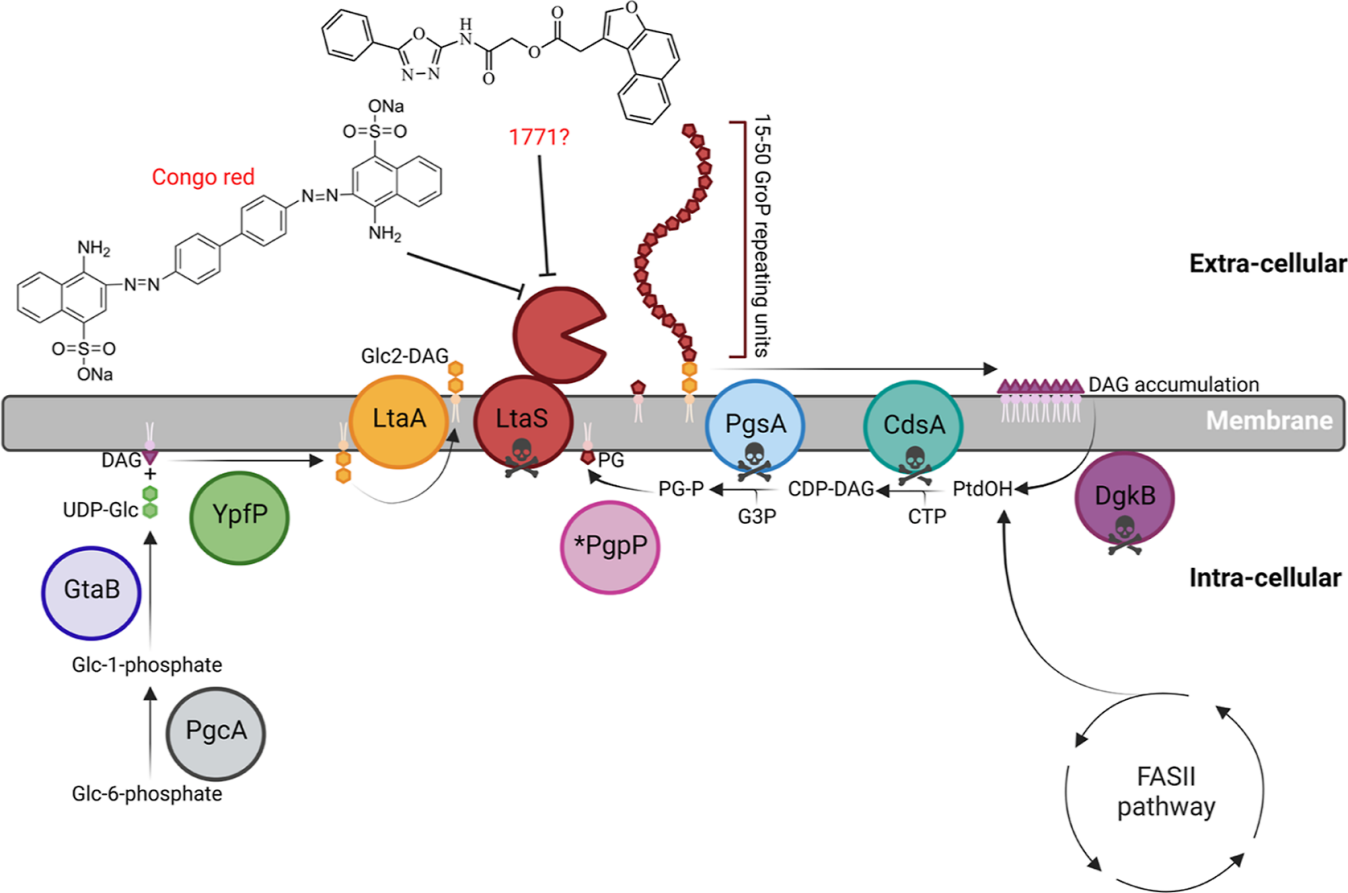
LTA biosynthesis pathway in *S. aureus*. LTA biogenesis begins with the synthesis of the diglucosyl-diacylglycerol (Glc_2_-DAG) anchor. PgcA and GtaB are responsible for generating nucleotide-activated sugar UDP-glucose. The diacylglycerol β-glucosyltransferase YpfP then combines DAG with UDP glucose to form the membrane anchor. The glycolipid permease LtaA subsequently transfers the Glc_2_-DAG anchor from the inner to the outer leaflet of the membrane. The LtaS enzyme then hydrolyzes the glycerolphosphate headgroup from the membrane lipid phosphatidyl glycerol (PG) and transfers between 15 and 50 of these moieties to the Glc_2_-DAG anchor. The hydrolysis of PG by LtaS results in the accumulation of DAG, which must be recycled to replace the loss of PG. This is achieved through the action of DgkB, which phosphorylates DAG into phosphatidic acid (PtdOH), which is also produced through the FASII pathway. Phosphatidic acid is fed into the phospholipid synthesis pathway through the action of CdsA and PgsA. CDP-diacylglycerol (CDP-DAG) is produced from PtdOH and cytidine triphosphate through the action of cytidylyltransferase CdsA. The CDP-diacylglycerol-glycerol-3-phosphate 3-phosphatidyltransferase PgsA then synthesizes phosphatidylglycerol-phosphate by replacing cytidine monophosphate with glycerol phosphate. A proposed but currently undiscovered protein designated as PgpP is believed to dephosphorylate phosphatidylglycerol-phosphate (PG-P) into PG. Essential proteins in this pathway are indicated with skull and crossbones and thought to include LtaS, DgkB, CdsA, and PgsA. LTA inhibiting compounds 1771 and Congo red are highlighted in red.

Previous studies have identified the 1,3,4 oxadiazole-based small molecule named 1771 (Figure 1), which has been shown to block LTA synthesis and inhibit in vitro growth of MRSA strains at concentrations between 8 and 16 μg/mL.^12^ This compound has also been shown to have activity against vancomycin-resistant *Enterococcus faecium* (VRE) strains.^12,20^ Although the first study demonstrating 1771 activity attributed this to LtaS inhibition,^12^ the target(s) of 1771 remains unclear. Vickery et al. reconstituted LtaS into artificial liposomes, observing LtaS polymerization activity and LTA production. Using this approach, 1771 did not show inhibitory activity; however, the azo dye Congo red blocked LtaS function.^11^ On the contrary, our^21^ and others^14^ in silico investigations highlighted that 1771 might behave as a competitive inhibitor of LtaS, in line with biophysical data indicating binding between 1771 and derivatives with the extracellular domain of LtaS (eLtaS).^12,14^

In this study, we sought to design and synthesize a small series of derivatives of 1771 with the aim to not only improve the antistaphylococcal activity of 1771 but also correlate the compound’s activity with its in silico interaction with LtaS. Furthermore, we investigated the molecular targets for 1771 and novel derivatives with an emphasis on establishing possible mechanisms of action of this class of antibacterial compounds.

## 2 Results

### 2.1 Chemistry

**Scheme 1:**
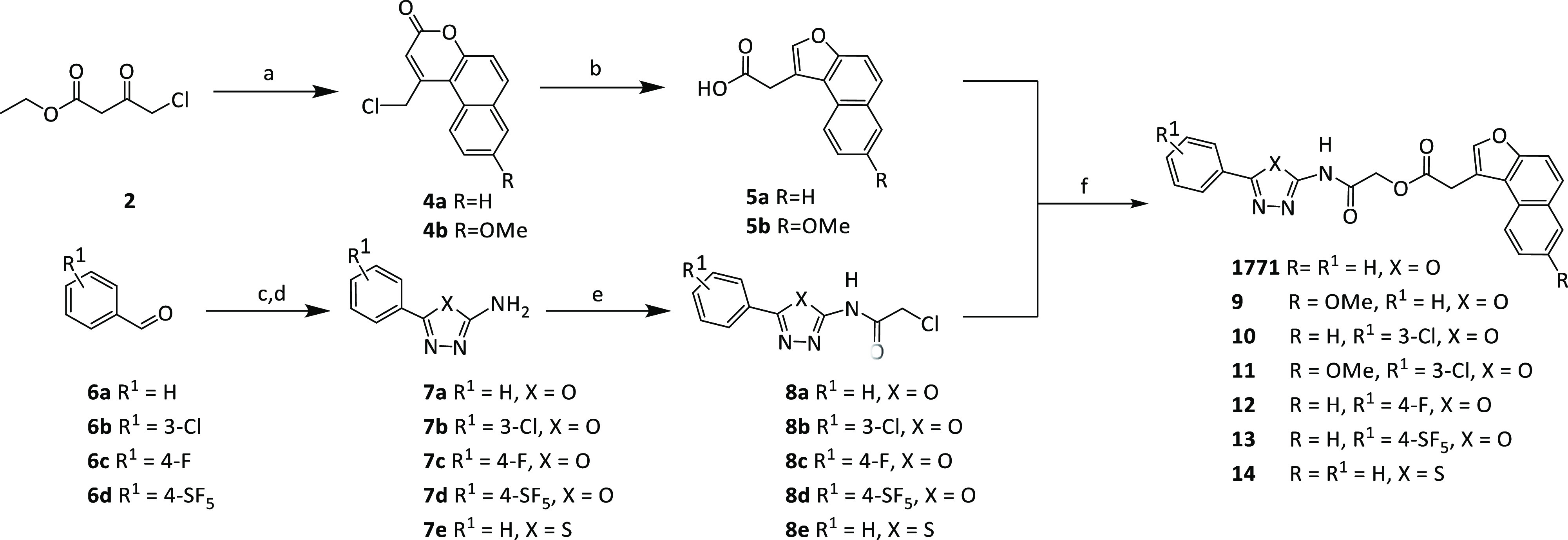
Synthesis of 1771 and Compounds **9–14** Reagents and conditions: (a) naphthalen-2-ol (**3a**) or 6-methoxynaphthalen-2-ol (**3b**), conc H_2_SO_4_, 0–5 °C, 24–72 h, 89–96%; (b) 1 M NaOH, reflux, 4 h, 43–93%; (c) semicarbazide hydrochloride, sodium acetate, MeOH, H_2_O, rt, 30 min, quant; (d) K_2_CO_3_, I_2_, 1,4-dioxane, 80–95 °C, 3–20 h, 36–60% over two steps; (e) chloroacetyl chloride, toluene, 80 °C, 18 h, 78–96%; (f) NaI, Et_3_N, DMF, 80–90 °C, 2–5 h, 4–25%.

Compounds **9–14** were synthesized using the same convergent synthesis method previously reported for 1771,^21^ as shown in Scheme 1. Briefly, transesterification of ethyl 4-chloro-3-oxobutanoate **2** with naphthalen-2-ol (**3a**) or 6-methoxynaphthalen-2-ol (**3b**) after Pechmann condensation yielded the corresponding desired angular chlorocoumarines **4a,b**, which under basic conditions underwent a rearrangement to give compounds **5a,b**. Condensation of a benzaldehyde (**6a–d**) with semicarbazide hydrochloride, followed by iodine-mediated oxidative cyclization, afforded 1,3,4-oxadiazole compounds **7a–d**. Compounds **7a–e** were reacted with chloroacetyl chloride in toluene affording compounds **8a–e**. The final step was accomplished by coupling the two building blocks (**5a,b** and **8a–e**) in the presence of triethylamine and a catalytic amount of sodium iodide in anhydrous dimethylformamide to afford the desired 1771 analogues **9–14**.

### 2.2 Derivatives of 1771 Show Improved Antibacterial Activity against Gram-Positive Organisms

In this study, we designed six derivatives of the LTA inhibitor 1771, named here as compounds **9–14**. To evaluate the antimicrobial activity of these new compounds, the minimum inhibitory concentration (MIC) was compared to 1771 using a genetically diverse collection of *S. aureus* and *Staphylococcus epidermidis* strains (Table 1). The MIC of 1771 ranged from 4 to 16 μg/mL against *S. aureus* and from 8 to 16 μg/mL against *S. epidermidis*. Compounds **9**, **10**, and **14** appeared to have poorer antistaphylococcal activity with a 4- to 16-fold increase in the MIC when compared to 1771. The MIC of **12** was comparable to that of 1771 against *S. aureus*, but it appeared to have slightly improved activity against the *S. epidermidis* strains tested. Compounds **11** and **13** showed superior antibacterial activity, with **13** displaying the highest potency with an MIC_90_ of 0.5 μg/mL against *S. aureus* and of 1 μg/mL against *S. epidermidis*.

**Table 1:** Compounds **11** and **13** Display Enhanced Antibacterial Activity against *S. aureus* and *S. epidermidis*

		minimum inhibitory concentration (μg/mL)					
strain	1771	**9**	**10**	**11**	**12**	**13**	**14**
S. aureus							
LAC	8	32–64	64	4	8	0.5	>64
MW2	8	64	64	2	8	0.5	>64
Newman	4–8	32	64	2	8	0.5	>64
SH1000	4–8	32	64	2	8	0.5	>64
MRSA252	8	64	64	4	16	1	>64
TW20	8	32	64	2	8	0.5	>64
Mu50	4–8	64	64	2	4–8	0.5	>64
Mu3	8–16	64	64	2	8	0.5	>64
EMRSA-15	8–16	64	32–64	2	8	0.5	>64
S. epidermidis							
RP62A	8–16	32	64	2–4	4–8	0.5	>64
311	8–16	64	32–64	2	4–8	0.5	>64
771	8–16	64	32–64	2	4–8	0.5	>64
780	8–16	32	32–64	4	4–8	0.5–1	>64
319	8–16	64	64	2	4–8	1	>64
322	16	64	64	4	8	0.5	>64
305	8–16	64	32–64	2–4	8	0.5–1	>64

Having identified **13** as the most potent derivative of 1771, we sought to test whether its activity also extended toward other Gram-positive species (Table 2). MICs of 1771 and **13** were repeated against a cohort of six Gram-positive species that comprised *Bacillus subtilis*, *Enterococcus faecalis*, *E. faecium*, *Streptococcus pyogenes*, *S. agalactiae*, and *Streptococcus dysgalactiae*. Compound **13** showed marked improvement in its anti-Gram-positive activity against *B. subtilis* (strain W168) and *E. faecalis* (strain JH2**–**2) but displayed similar activity to 1771 against *S. pyogenes*, *S. agalactiae*, and *S. dysgalactiae* isolates with a twofold decrease in the MIC.

**Table 2:** Compound **13** Displays Enhanced Activity against Other Gram-Positive Species

	MIC (μg/mL)	
bacterial species (strain)	1771	**13**
B. subtilis (W168)	4–8	0.5–1
E. faecalis (JH2-2)	16	2
E. faecium (C68)	>128	>128
S. pyogenes (NCTC 8198)	32–64	16–32
S. pyogenes (NCTC 12048)	128	64
S. agalactiae (18R521)	128	64
S. agalactiae (COH1)	>128	128
S. dysgalactiae (NCTC 13762)	128	64
S. dysgalactiae (NCTC 10238)	>128	64

To evaluate the synergistic potential of 1771 and **13**, checkerboard MIC analysis was performed in combination with five clinically relevant antistaphylococcal antibiotics (oxacillin, daptomycin, vancomycin, gentamicin, and linezolid) and FIC indexes were calculated (Figure S1). The only synergistic combination observed was gentamicin in combination with 1771 with an FIC index of 0.375.

### 2.3 1771 and Its Derivatives Inhibit Biofilm Formation

**Figure 2:**
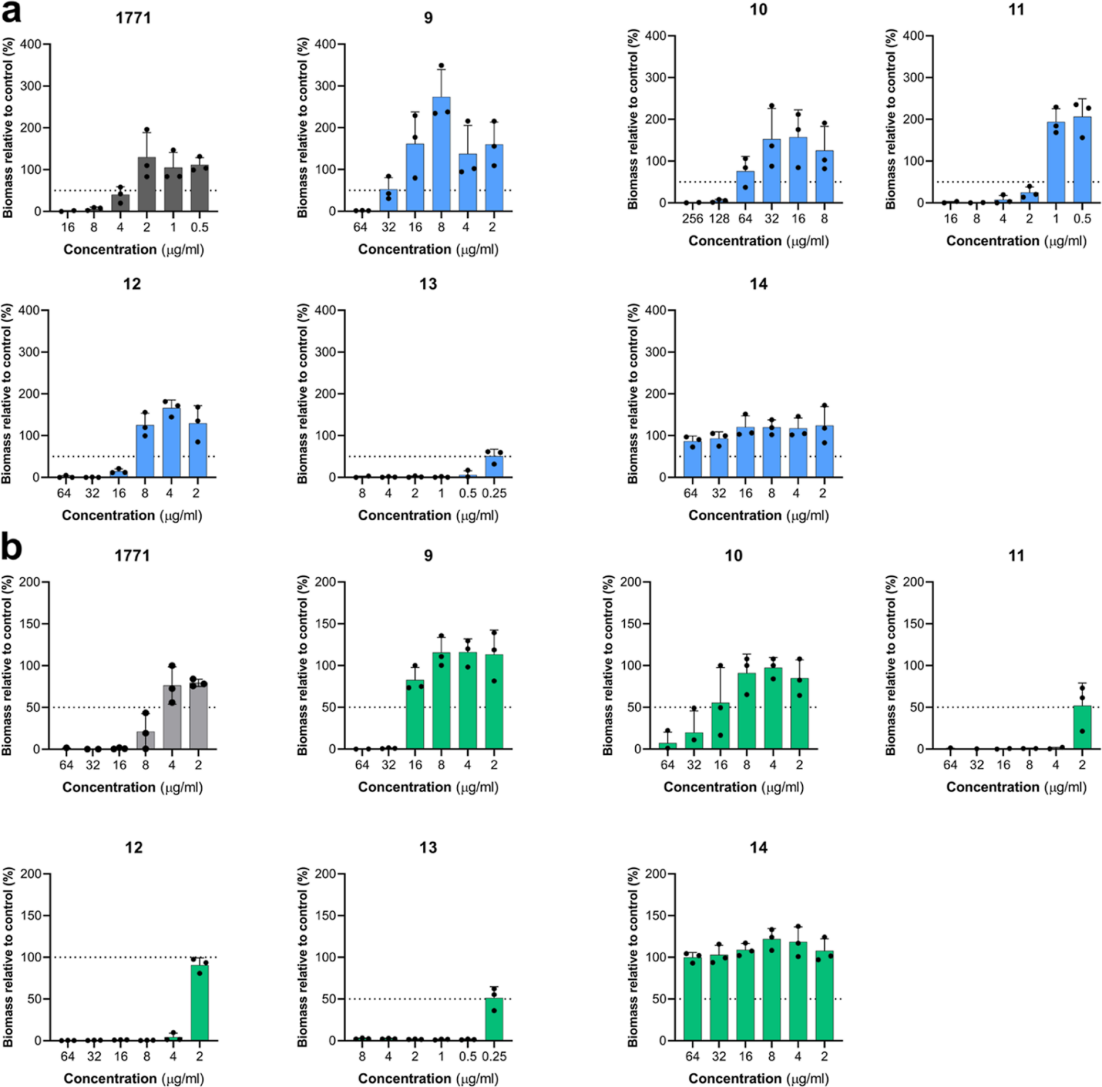
Biofilm prevention ability of 1771 and its derivatives **9–14**. The biofilm prevention ability of 1771 and compounds **9–14** against (a) *S. aureus* strain SH1000 and (b) *S. epidermidis* strain RP62A was examined using the crystal violet assay. 1771 and compounds **9–14** were added at a final concentration ranging from 256 to 0.25 μg/mL. Absorbance at 595_nm_ was recorded following 24 h incubation, and the biomass (%) relative to the negative control without antibiotic was calculated. The dots represent biological replicates, with the bars representing the mean biomass value and the error bars representing the standard deviation.

*S. aureus* and *S. epidermidis* routinely form sessile microbial communities called biofilms. Biofilms are a frequent cause of chronic and persistent infections and are refractory to clinically used antibiotics. Accordingly, we investigated the biofilm-prevention capabilities of 1771 and the newly synthesized derivatives against methicillin-sensitive *S. aureus* strain SH1000 and *S. epidermidis* strain RP62A, two previously characterized strong biofilm formers^22,23^ (Figure 2). We observed a 50% reduction in biofilm formation of SH1000 and RP62A following treatment with 1771 at a concentration between 4 and 8 μg/mL, respectively. Compounds **9**, **10**, and **14** all performed poorly at biofilm prevention, requiring concentrations of 32–64 μg/mL for SH1000 and 16–64 μg/mL for RP62A to reduce biofilm production by 50%. Compound **12** performed worse than 1771 in preventing SH1000 biofilm production but better in preventing RP62A biofilm production. Similar to the MIC analysis, **11** and **13** performed better than 1771 at preventing the biofilm production of SH1000 and RP62A, with **13** requiring the lowest concentration of 0.25 μg/mL to cause a 50% reduction in biofilm formation in both SH1000 and RP62A.

### 2.4 1771-Based Compounds Are Bacteriostatic

**Figure 3:**
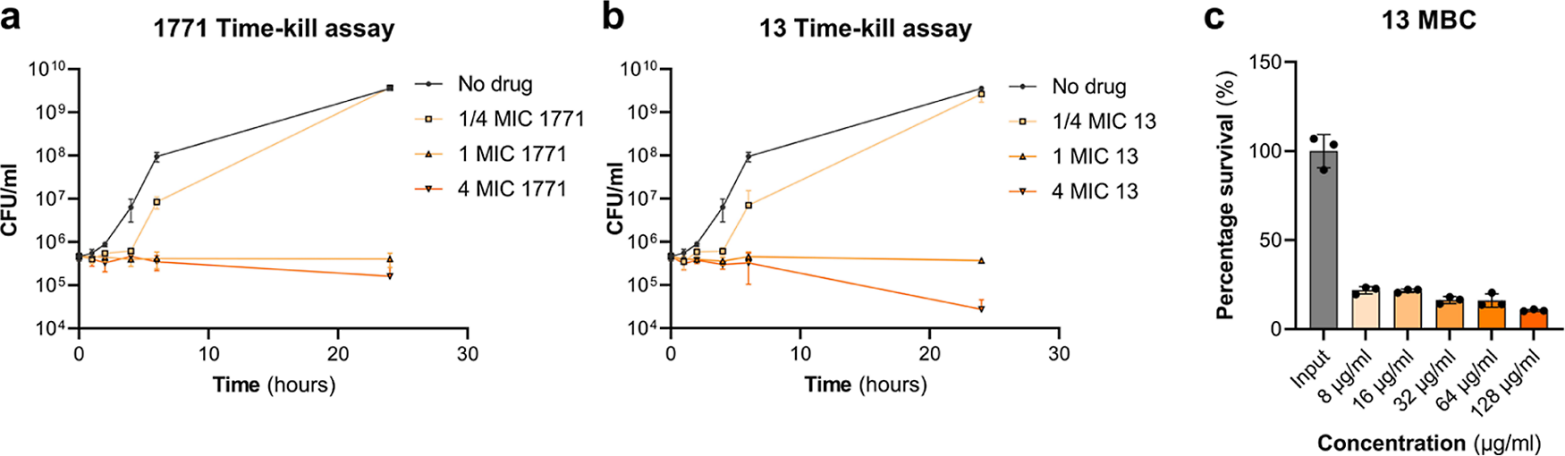
1771 and **13** display limited bactericidal activity. Time-kill kinetics of (a) 1771 and (b) **13** against LAC using a starting inoculum of 5 × 10^5^ cfu. LAC was incubated in MHB containing no drug and 0.25×, 1×, and 4× MIC of either 1771 or **13**. Symbols represent the mean, and error bars represent the standard deviation. (c) Minimum bactericidal concentration of **13**. Following application of the MIC protocol, 50 μL of suspension was plated out, and the percentage survival was calculated according to the 5 × 10^5^ cfu starting inoculum. The dots represent individual data points, the bars represent the mean value, and the error bars represent the standard deviation.

Since its discovery as a compound that inhibits *S. aureus* growth, the kill kinetics of 1771 is yet to be investigated in detail. To answer this, a time kill assay was performed against 1771 and the most active derivative **13** (Figure 3a,b). MRSA strain LAC was incubated in MHB containing 0.25×, 1×, 2×, or 4× MIC of 1771 or **13**, and CFUs were plated on TSA for enumeration at 0, 1, 2, 4, 6, and 24 h. As expected, LAC was able to grow in the presence of 0.25× MIC of 1771 and **13**, although the growth rate was impacted (Figure 3a,b). Incubation in 1× MIC of either 1771 or **13** completely inhibited the growth of LAC; however, no killing effect was observed, with the inoculum staying at approximately 5 × 10^5^ cfu over the 24 h period. A killing effect of roughly 65% was observed for LAC incubated with 4× MIC 1771. This was much more evident for LAC incubated with 4× MIC of **13**, where only 1% of the starting inoculum survived the exposure. For an antibiotic to be classified as bactericidal, treatment must result in a greater than 3-log_10_ reduction in CFU.^24^ Accordingly, the minimal bactericidal concentration (MBC) of **13** was examined (Figure 3c) using concentrations from 8 to 128 μg/mL. Despite increasing the exposure of LAC to 256× MIC of **13**, a proportion of the starting inoculum consistently survived, meaning that an MBC could not be elucidated. This further supports the classification of 1771 and **13** as bacteriostatic agents.

### 2.5 1771 and **13** Exhibit Minimal Toxicity and Display a Low Propensity to Develop Resistance

**Figure 4:**
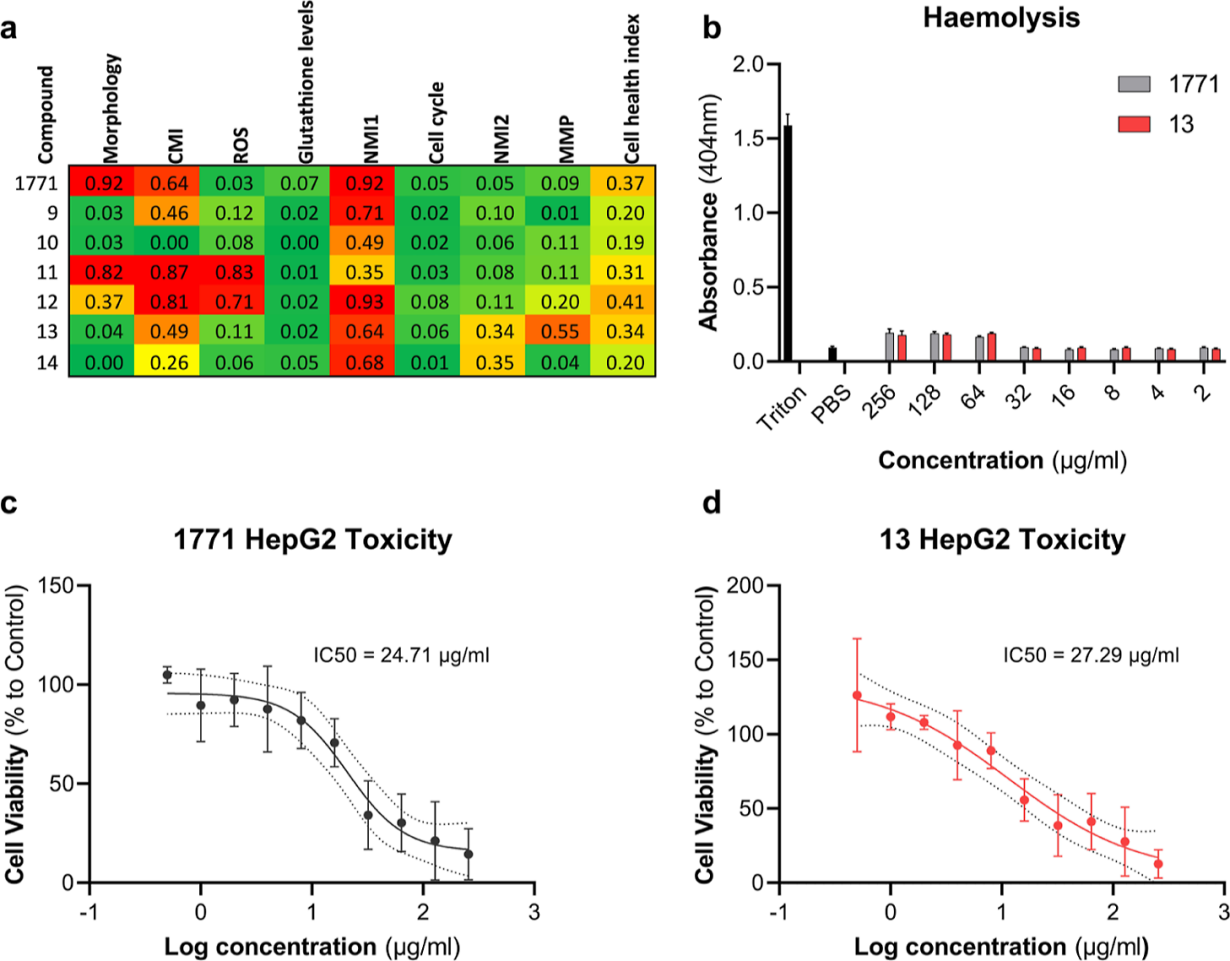
Toxicity profile of 1771 and compounds **9–14**. (a) Cell health screen of 1771 and compounds **9–14** using the AsedaSciences SYSTEMETRIC assay. (b) Hemolysis assay of 1771 and **13** at a concentration range of 256–2 μg/mL. Maximum erythrocyte lysis is provided by incubation with Triton X100, and minimum lysis is provided by incubation with PBS. Log 1771 (c) and **13** (d) concentration was plotted against cell viability of HepG2 cells following 24 h incubation with the relevant compound. Dots represent the mean, and the error bars represent the standard deviation. The line is a nonlinear regression, with the dotted lines representing the 95% confidence interval of this analysis. The IC_50_ value was calculated through standard curve interpolation and defined as the concentration responsible for 50% HepG2 cell death.

A key feature of any prospective antibiotic is that the compound has specific toxicity toward bacterial cells at low concentrations while displaying minimal toxicity to host cells. In this study, we screened 1771 and compounds **9–14** for acute toxicity using the AsedaSciences SYSTEMETRIC Cell Health Screen.^25,26^ This system measures eight parameters using a fluorescent readout of multiple dyes that increase or decrease the fluorescence on a per cell basis, depending on how a compound affects that specific biological parameter. The parameters measured included cell morphology, cytoplasmic membrane integrity (CMI), mitochondrial reactive oxygen species (ROS), glutathione levels, nuclear membrane integrity (two dyes are used, NMI 1–2), cell cycle activity, and mitochondrial membrane potential (MMP). Overall, all compounds were within the lower toxicity risks where the cell health index (CHI), a final probability score that quantifies the similarity of a test compound’s cell stress phenotype to the high-risk class in the training set,^25,26^ was below 0.5 (Figure 4a). 1771, **9**, and **12–14** did score high on one of the NMI tests, indicating potential cellular damage. In addition, **10** and **11** gave a high readout for both ROS and CMI, indicating potential intracellular membrane damage. Lastly, both 1771 and **11** were associated with morphological changes that were not observed with the other compounds. Notably, other clinically relevant antibiotics used in the training set indicated similar CHI values to our compounds: vancomycin (CHI = 0.16), streptomycin (CHI = 0.19), and erythromycin (CHI = 0.22) (Table S2).

Antibiotic compounds are usually metabolized by the liver and can in certain cases, such as with rifampicin and colistin, cause drug-induced liver injury or antibiotic-induced hepatotoxicity.^27^ To further evaluate the suitability of 1771 and **13** as antibiotic agents, the toxicity of these compounds was analyzed using the HepG2 human hepatocyte cell line (Figure 4c,d). 1771 gave a HepG2 IC_50_ of 24.71 μg/mL, which given its MIC_50_ of 8 μg/mL gives a selectivity index (IC_50_/MIC_50_) of 3.08. Compound **13** was slightly less toxic, with a HepG2 IC_50_ of 27.29 μg/mL. However, the MIC_50_ of compound **13** was 0.5 μg/mL, giving a significantly improved selectivity index of 54.28. This shows that while 1771 has been shown to be nontoxic to other human cell lines,^12^ it is relatively toxic toward liver cells, restricting its potential as a Gram-positive antibiotic. Compound **13** on the other hand, due to its 16- to 32-fold increase in potency, has a robust therapeutic window in which it would be selective to bacterial cells over human cells. 1771 and **13** were also tested against erythrocytes (Figure 4b), where neither caused any lysis at concentrations as high as 256 μg/mL.

A limiting therapeutic value of many existing antibiotics is the propensity of bacteria to acquire resistance due to spontaneous mutations. The work of Richter et al. has shown that incubation with 1771 for 3–4 days did not result in resistant mutant selection, which was in stark contrast to streptomycin, which was selected for mutants at a frequency of <10^–7^.^12^ To further evaluate whether *S. aureus* could acquire resistance to 1771, a serial passaging experiment was performed (Figure S2). Following 25 days of serial passage, the MIC increased twofold against both LAC and EMRSA-15 after 14 days and remained stable for 11 days, indicating that these 1,3,4-oxadiazole-based antibiotics are potentially resistance-proof compounds.

### 2.6 1771 and **13** Are Inhibitors of LTA Synthesis

Incubation with 1771 has been shown to cause a dose-dependent reduction in the LTA signal.^12^

**Figure 5:**
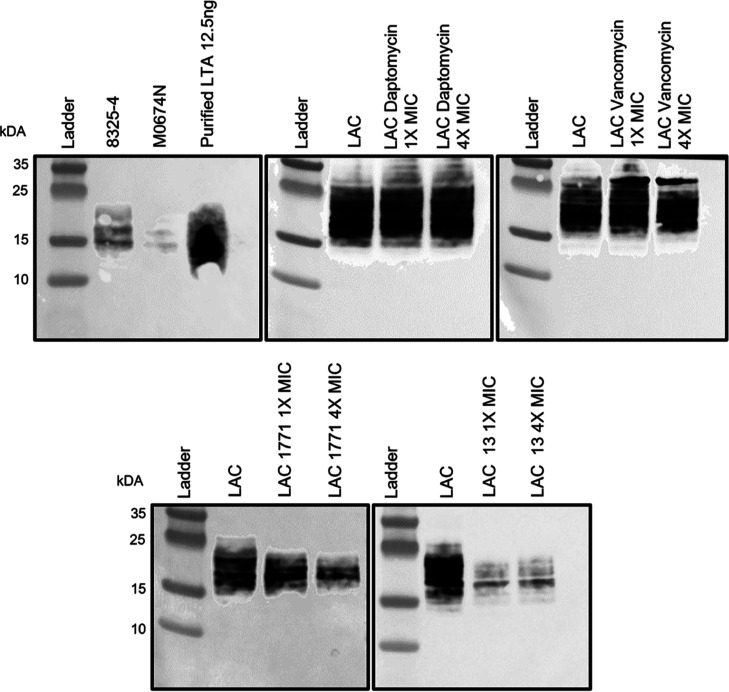
LTA inhibitory activity of 1771 and **13**. The suitability of the LTA antibody was first tested against the isogenic strains 8325-4 and M0674N alongside 12.5 ng of purified LTA. The LTA inhibitory ability of 1771 and **13** was compared to that of non-LTA inhibitory antibiotics daptomycin and vancomycin at 1× and 4× the relevant MIC.

We were interested in testing whether **13** also resulted in a reduction in the LTA signal and whether **13** was superior at inhibiting LTA biosynthesis compared to 1771. The anti-LTA antibody was first tested against 8325-4 (wild-type) and *ltaS* mutant M0674N alongside purified LTA. The 8325-4 strains gave a band between 15 and 25 kDa, which was absent in strain M0674N, confirming the suitability of the antibody (Figure 5). We next tested whether any reduction in LTA signal could be observed for daptomycin and vancomycin, two antibiotics not known for their LTA inhibitory activity but that target the bacterial cell envelope. No reduction in LTA could be seen for strain LAC incubated with 1× and 4× the MIC of daptomycin or vancomycin. Conversely, when strain LAC was incubated with 1× and 4× the MIC of 1771 or **13**, a reduction in LTA abundance was observed (Figure 5). The LTA inhibitory action of **13** was more apparent compared with 1771, with a greater reduction in abundance at the 1× MIC and consistent with the enhanced activity observed in the MIC analysis.

### 2.7 Molecular Modeling of Compounds **9–14** with LtaS

**Figure 6:**
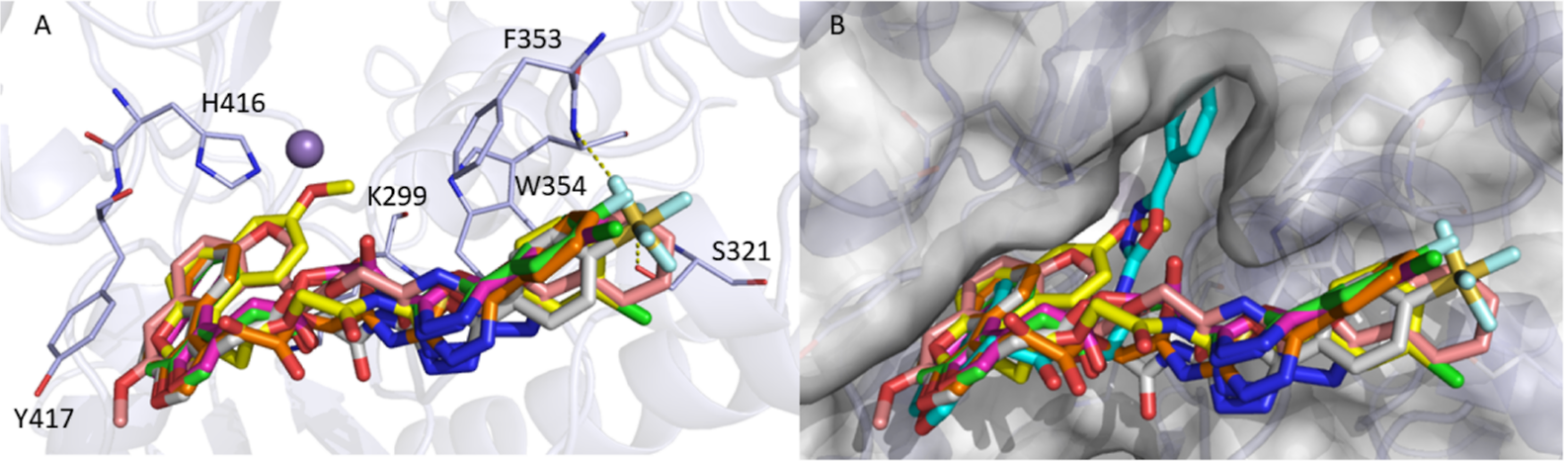
Molecular modeling of 1771 and compounds **9–14** with eLtaS. (a) Binding mode of compounds **9** (pink), **10** (magenta), **11** (yellow), **12** (green), **13** (gray), and **14** (orange) within the LtaS active site. The residues of the binding pocket involved in the interactions with the ligands are represented as light blue sticks. H-bonds are displayed as yellow dashed lines. (b) Docking pose of 1771 (cyan sticks) superimposed to derivatives **9–14**.

The putative binding mode of compounds **9–14** within the LtaS active site was investigated by molecular docking employing the X-ray structure of the extracellular domain of the enzyme in complex with glycerol-phosphate (PDB ID 2w5s).^15^ Despite the extracellular domain containing the catalytic site of LtaS, experimental evidence indicated the importance of the transmembrane (TM) domain for enzyme function.^28^ On this basis, we analyzed the LtaS model retrieved from AlphaFold repository^29^ (AF-Q7A1I3-F1), which includes the TM domain. As depicted in Figure S3, the residues 106–107 and 109–112 of the AlphaFold structure, which belong to the TM domain, were predicted with a low confidence score (70 > pLDDT < 50). Since these residues are located in close proximity to the binding site, we discarded the AlphaFold structure as model for docking studies. On the contrary, the X-ray extracellular domain structure proved to be a reliable template for structure-based modeling studies.^14^ The docking outcomes revealed that derivatives **9–14** may interact with the LtaS binding pocket assuming a similar orientation as displayed in Figure 6a. The phenyl ring occupies a niche of the pocket lined by W354, F353, and S321, while the naphtho[2,1-*b*]furan moiety is situated close to H416 and Y417. All the synthesized compounds may establish hydrophobic and/or π-stacking interactions with (i) F353 and W354 through the phenyl portion and (ii) H416 and Y417 through the naphtho[2,1-*b*]furan system. Moreover, hydrophobic contacts between naphtho[2,1-*b*]furan and K299 were detected for **9** and **11**. In addition, the SF_5_ group of **13** could form hydrogen bonds with the side chain of S321 and the backbone of W354.

The docking poses of the newly synthesized derivatives **9–14** was compared to that of the parent compound 1771 previously reported.^21^ As shown in Figure 6b, the phenyl-1,3,4-oxadiazole portion of 1771 (cyan sticks) can fit into a deep part of the LtaS active site, while the same moiety is located in the rim of the pocket in the case of compounds **9–14**. Significantly, the oxadiazole ring, which appeared to play a pivotal role in the binding to LtaS in the docking model reported by both us^21^ and Chee Wezen et al.,^14^ is not involved in the interaction between the enzyme and compounds **9–14**.

### 2.8 1771 and **13** Display Antimicrobial Activity Independent of LtaS Function

To better understand whether 1771 and **13** exert their LTA inhibitory activity through an interaction with LtaS, we examined both the impact of deleting and overexpressing LtaS on drug susceptibility (Table 3a,b). Mutants lacking LtaS are hypersusceptible to osmotic lysis and can only be grown under osmotically stabilizing conditions at 30 °C^19^ or after the acquisition of suppressor mutations.^30−32^ One such suppressor mutation is the loss of the ClpX chaperone. Indeed, in ClpX mutants, *ltaS* mutations arise spontaneously at 30 °C and reverse the growth defect associated with ClpX loss.^30^ Making use of this conditional essentiality, we tested three isogenic sets of strains consisting of a WT, a ClpX mutant, and a ClpX/LtaS double mutant (Table 3a). Deletion of ClpX reduced the MIC of 1771 from 16 to 8 μg/mL and the MIC of **13** from 0.5 to 1 to 0.25 μg/mL in the 8325-4 background. Deletion of ClpX had no impact on the susceptibility of 1771 or **13** in the SA564 background. We hypothesized that a true LtaS inhibitor would not be able to inhibit the growth of a ClpX/LtaS double mutant. However, the ClpX/LtaS double mutants exhibited the same 1771 and **13** MIC as those of the ClpX single mutants. A slight increase in MIC was observed for 1771 (8 to 16 μg/mL) and **13** (0.25 to 0.5 μg/mL) against SA564*clpX* knockout and *ltaS*H476Q mutant compared with the JE2 *clpX*I265E mutant. We also tested the susceptibility of an LtaS deletion mutant M0674N against 1771 and **13** and found that deletion of *ltaS* in this background had no impact on MIC.

**Table 3:** (a,b) Impact of LtaS Deletion and Overexpression on the Susceptibility of *S. aureus* to 1771 and **13**

(a)		
strain	MIC (μg/mL)	
	1771	**13**
8325-4 (wild type)	16	0.5–1
8325-4 *clpX* knockout mutant	8	0.25
8325-4 *clpX* knockout and *ltaS*382STOP mutant	8	0.25
SA564 (wild type)	16	0.25
SA564 *clpX* knockout mutant	16	0.25
SA564*clpX* knockout and *ltaS*H476Q mutant	16	0.25
JE2 (wild type)	16	0.5
JE2 *clpX*I265E mutant	8	0.25
JE2 *clpX*I265E and *ltaS*339STOP mutant	16	0.5
8325-4 (wild type)	8	0.5
M0674N (*ltaS* knockout mutant in 8325-4)	8	0.5

(b)			
strain	Congo red	MIC (μg/mL)	
		1771	**13**
Newman	>512	16	1
Newman Δ*tarO*	64	16	1
Newman Δ*tarO* pRMC2	64	16	1
Newman Δ*tarO* p*ltaS*	256	16	1

Congo red has been shown to inhibit the activity of LtaS in vitro.^11^ Unlike 1771, Congo red is only toxic to TarO mutants, leading the authors to suggest that LtaS inhibition is only synthetically lethal in WTA-deficient strains.^19,33^ Similarly, we show that Congo red only has antistaphylococcal activity when TarO is deleted, while 1771 and **13** exhibit the same MIC as the WT Newman strain when TarO is deleted (Table 3b). We subsequently hypothesized that overexpression of *ltaS* would confer resistance or at least result in an elevation in MIC, against an LtaS inhibitor. Overexpression of *ltaS* in the Newman TarO mutant did show increased LTA expression by Western blot compared to controls (Figure S4). In addition, overexpression of *ltaS* resulted in an increase in the Congo red MIC from 64 μg/mL in Newman Δ*tarO* pRMC2 to 256 μg/mL in Newman Δ*tarO* p*ltaS*. However, the overexpression of *ltaS* has no impact on the 1771 or **13** susceptibility, indicating that these compounds exert an inhibitory effect on other targets.

### 2.9 1771 and Compound **13** Have Antimicrobial Activity against Gram-Negative Bacteria

**Figure 7:**
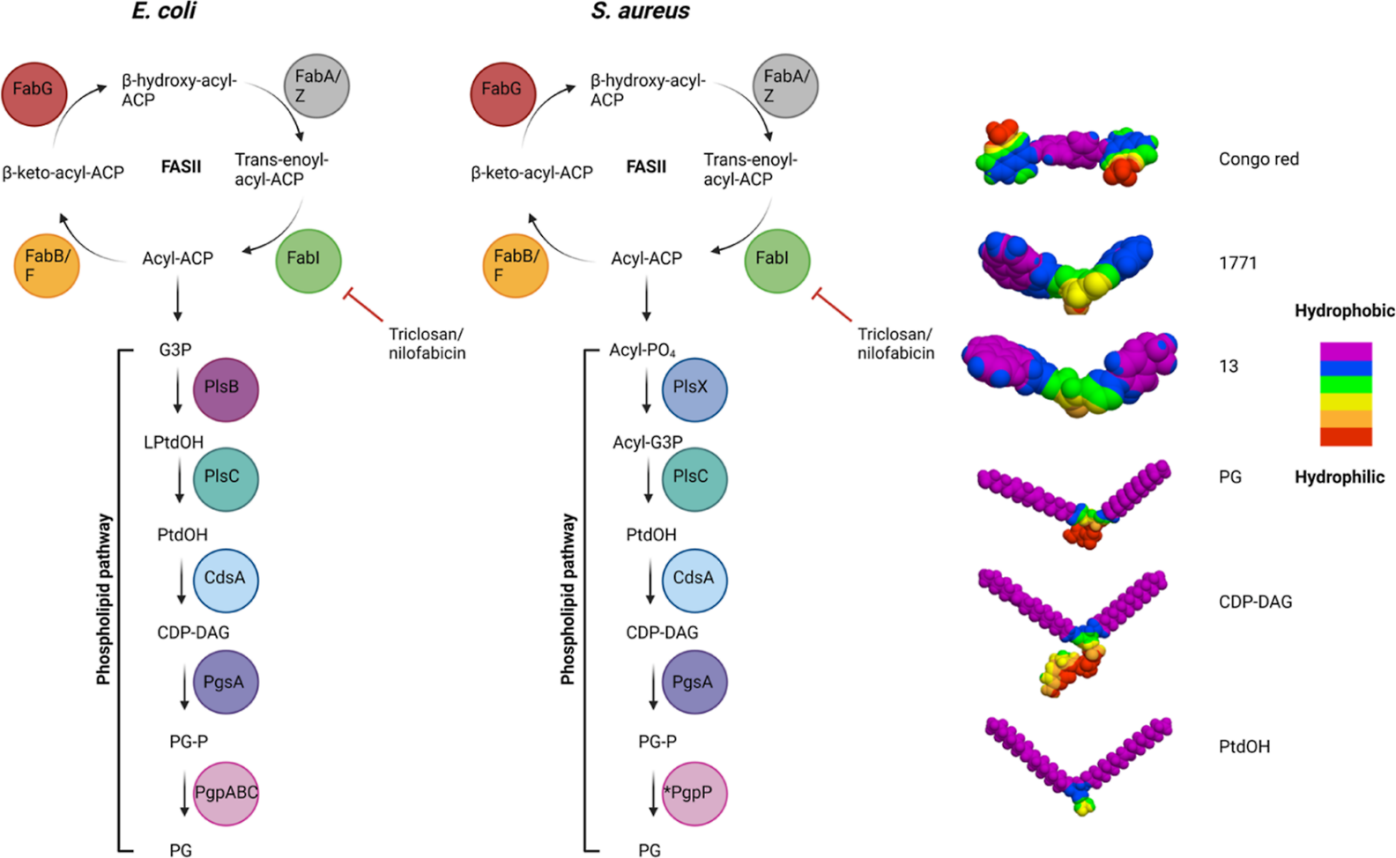
Similarities of the *E. coli* and *S. aureus* FASII/phospholipid biosynthetic pathway. 3D model highlighting the molecular hydrophobicity potential (MHP) of Congo red, **13**, and 1771 and the PG, CDP-DAG, and PtdOH lipids. Color coding for hydrophobic and hydrophilic residues is shown in the key. Models were made using the Molinspiration Galaxy 3D Structure Generator v2022.11 software.

Phosphatidyl glycerol (PG) head groups are used as the building blocks of LTA; therefore, a constant supply of PG is required for LTA biosynthesis.^9^ As such, the upstream enzymes of the phospholipid biosynthetic pathway, which produce PG, are necessary to produce LTA and thus are targets for LTA inhibition.^34^ We confirmed this by incubating *S. aureus* with fatty acid inhibitors, nilofabicin or triclosan, both of which target the enoyl-acyl carrier protein reductase FabI of the FASII pathway (Figure 7),^35,36^ which resulted in a substantial decrease in LTA production (Figure S5).

Previous work examined the molecular hydrophobicity of 1771 and noted that the naphthofuranyl groups and aryl 1,3,4 oxadiazolyl acetamide group resembled the substrate for LtaS, PG, reinforcing the hypothesis that 1771 targeted LtaS.^12^Figure 7 portrays 3D models indicating the molecular hydrophobicity potential of LTA inhibitors, Congo red, 1771, and **13** as well as components central to both phospholipid and LTA biosynthesis: PG, CDP-DAG, and PtdOH. Here, we observe that structural similarity exists when comparing PG, CDP-DAG, and PtdOH and that 1771 and **13** have a greater structural resemblance to these intermediates of the phospholipid pathway compared to Congo red. Thus, our data suggest that the LTA inhibitors (1771 and **13**) may target components of phospholipid biosynthesis and not solely PG, resulting in LTA disruption and bacterial death.

Several Gram-positive antibiotics have been shown to have Gram-negative activity if the outer membrane is first breached^37^ or efflux pumps are inactivated or bypassed.^38^ For example, the Gram-positive-specific FabI inhibitor afabicin (Debio-1452) has been successfully converted into a derivative, Debio1452-NH_3_, which can accumulate within and target the FASII/phospholipid biosynthetic pathway in Gram-negatives^39^ as these pathways are remarkably similar, as shown in Figure 7.

**Figure 8:**
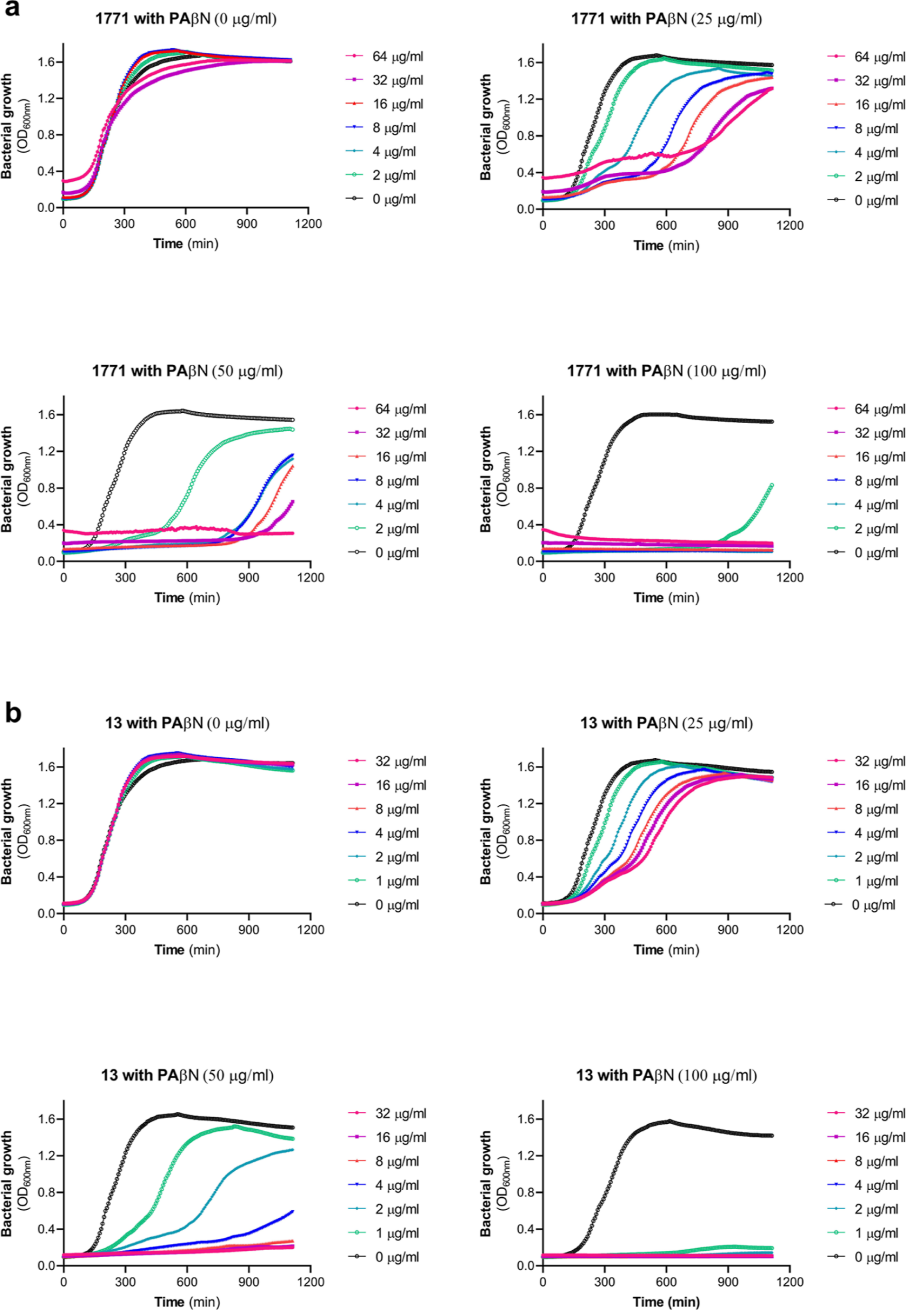
Permeabilization of the outer membrane renders *E. coli* susceptible to 1771 and **13**. Bacterial growth curves of (a) 1771 and (b) **13** (0–64 μg/mL) in combination with 0, 25, 50, and 100 μg/mL of PAβN. The bacterial growth (OD_600nm_) was plotted against time following 18 h of growth at 37 °C. The icons represent the mean of three biological replicates.

In the initial small-molecule library screen, 1771 was identified as it selectively inhibited the growth of *S. aureus* while having no effect on the growth of *Escherichia coli*.^12^ We wanted to investigate whether the reason 1771 did not inhibit *E. coli* growth was due to poor penetration of the outer membrane rather than the absence of the target. Phenylalanine-arginine β-naphthylamide (PAβN) is a well-studied efflux pump inhibitor, which can also permeabilize the outer membrane of Gram-negative organisms.^40^ To test whether 1771 or **13** displayed any growth inhibition toward *E. coli*, these compounds were combined with either 0, 25, 50, or 100 μg/mL of PAβN and monitored for growth over 18 h (Figure 8). Without the addition of PAβN (0 μg/mL), neither 1771 nor **13** had any growth inhibitory activity at concentrations up to 64 μg/mL. With the addition of 25 μg/mL of PAβN, the *E. coli* strain K12 could still grow in the presence of 64 μg/mL of 1771 or **13**; however, the growth rate was severely impacted in a dose-dependent manner. When 50 μg/mL PAβN was added, growth was completely inhibited at a concentration of 64 μg/mL for 1771 and compound **13**. Finally, 1771 and **13** in combination with 100 μg/mL of PAβN resulted in MIC values of 4 and 1 μg/mL, respectively. Importantly, the addition of PAβN up to a concentration of 100 μg/mL had a very limited impact on growth, indicating the growth inhibitory activity was specific to 1771 or **13**, confirming that these compounds possess anti-Gram-negative activity when the outer membrane is disrupted. To further evaluate the Gram-negative coverage of 1771 and **13**, we also tested 1771 or **13** in combination with PAβN against *Pseudomonas aeruginosa* strain PAO1, *Klebsiella pneumoniae* strain 699 (clinical isolate), and *Acinetobacter baumannii* strain DF1000 (clinical isolate) (Figure S6a–c). An MIC of 4 μg/mL was observed in combination with 100 μg/mL PAβN for strains 699 and DF1000, while PAO1 growth was severely inhibited with 32 μg/mL **13** in combination with 100 μg/mL PaβN, indicating that LtaS cannot be the sole target of these antibiotics.

## 3 Discussion

A primary aim of this study was to investigate whether substitutions at the phenyl and/or naphtho[1,2-*b*]furanyl rings could improve the antibacterial activity of 1771. Specifically, we have looked at chloro, fluoro, and pentafluorosulfanyl (SF_5_) groups at the phenyl ring and methoxy substituent at the naphtho[1,2-*b*]furanyl ring. We also investigated whether replacing 1,3,4-oxadiazole with a 1,3,4-thiadiazole ring altered the antimicrobial activity. As a result, we generated a series of molecules with varying activity against a panel of clinically relevant staphylococcal isolates. In general, substitutions to the phenyl ring show retention or increase in the antibacterial activity, whereas the methoxy substitution on the naphtho[1,2-*b*]furanyl ring shows a significant decrease of the compound activity. Replacement of the oxygen of the 1,3,4-oxadiazole ring with a sulfur atom led to complete loss of antibacterial activity, indicating that the 1,3,4-oxadiazole moiety is essential for activity. Importantly, **13** bearing a pentafluorosulfanyl group (SF_5_) is, to our knowledge, among the most potent derivatives of 1771, displaying an MIC of 0.5–1 μg/mL against multidrug-resistant *S. aureus*, including MRSA and VRSA, a 16- to 32-fold increase in activity compared to that of 1771. Due to the distinctive combination of electronegativity, size, and lipophilicity, the fluorine atom can have a substantial impact on the molecular conformation of organic molecules, which may affect the binding affinity to the target protein.^41^ Recent years have seen an increased use of higher polyfluorinated groups in medicinal chemistry.^42^ Among them, SF_5_ is considered as a CF_3_, *tert*-butyl, halogen, or nitro group bioisostere, is stable under physiological conditions, and possesses unique physical and chemical properties such as high electronegativity coupled with an unusual lipophilicity and a higher antibacterial activity.^43,44^ SF_5_ has also a different electron density profile (pyramidal for SF_5_ opposite to spherical for CF_3_) as well as a larger molar volume than CF_3_.^45^

We have confirmed that **13** specifically inhibits LTA biosynthesis and that LTA inhibition is not a general feature of cell envelope-acting antibiotics but is disrupted following challenge with fatty acid inhibitors. Importantly, we have tested the toxicity of 1771 and its derivatives against multiple mammalian cell types using the AsedaSciences SYSTEMETRIC assay as well as classically used HepG2 liver cells and human red blood cells. Our toxicity analysis revealed that **13** exhibited limited toxicity, in line with toxicity values derived from clinically used antibiotics such as linezolid.^46^ Importantly, **13** displayed a superior selectivity index over 1771 primarily based on the improved antimicrobial activity associated with this molecule. 1771 and derivatives are reported to be synergistic with daptomycin and gentamicin against *E. faecium*(47) and with methicillin and carbenicillin^14^ against *S. aureus*. Our analysis showed limited synergistic potential with clinically relevant antibiotics, with 1771 displaying synergy only with gentamicin.

Several studies have investigated the mode of action of 1771. Richter et al. observed that 1771 inhibited eLtaS binding to and cleavage of PG in vitro using size exclusion chromatography, nitro-benzoxadiazole glycerol-phosphate as a substrate for eLtaS, and mass spectrometry.^12^ We^21^ and others^14^ have performed molecular docking of 1771 in the eLtaS catalytic site. Overall, both in silico investigations highlighted that 1771 might function as a competitive inhibitor of LtaS and emphasized the key role of the oxadiazole ring in the ligand–protein recognition process, in agreement with the experimental studies according to which this moiety is crucial for activity.^12^ However, when we studied the binding mode of compounds **9–14** within the LtaS active site, we observed that the phenyl-oxadiazole portion is located at the entrance of the pocket leading to the loss of some key interactions with the extracellular catalytic domain of the enzyme. Chee Wezen et al. used biophysical assays employing differential scanning fluorimetry and isothermal titration calorimetry and observed binding of 1771 and derivatives to eLtaS.^14^ In contrast, studies employing cell-free, proteoliposome-based systems, whereby LtaS was reconstituted and LtaS polymerization activity was examined, have shown that 1771 did not interrupt LTA biosynthesis, whereas Congo red displayed inhibition.^11^ In addition, other studies showed that an LtaS inhibitor would be synthetically lethal in a WTA-deficient strain.^19,33^ Vickery et al. found this was the case for Congo red, but 1771 was found to kill both wild-type and WTA-deficient strains.^11^ The authors of this study concluded that 1771 must exert its LTA depletory effects by targeting one or more enzymes required for polymer production such as enzymes involved in the biosynthesis of PG. This hypothesis aligns with our results that show that treatment with fatty acid inhibitors, nilofabicin, or triclosan resulted in a decrease in LTA abundance. To determine the requirement of LtaS function for 1771 and **13** activity, we tested these compounds against several *S. aureus* LtaS mutants (Table 3a,b). To our surprise, we did not see any significant difference in the MIC of 1771 or **13** when wild-type or *ltaS* mutants were challenged. Furthermore, we tested *S. aureus* strains that were engineered to overexpress *ltaS* from a chemically inducible promoter and found no difference in the MIC of 1771 or **13** compared with WT strains and empty vector control. Interestingly, overexpression of LtaS in a *S. aureus* TarO mutant conferred increased resistance to Congo red, which corroborates with previous studies indicating LtaS as a target for Congo red.^11^ Currently, the precise mechanism of action of 1771 is not completely understood, and while our data provide evidence that LtaS is not the sole target of 1771, we cannot disregard the possibility that LtaS may be one of multiple targets that is inhibited by 1771.

The initial study that illustrated the antimicrobial activity of 1771 showed no activity against *E. coli*.^12^ Importantly, Gram-negative bacteria are intrinsically resistant to numerous antibiotic classes due to the selective barrier imposed by the outer membrane and/or the activity of efflux pumps.^48^ When the integrity of this permeability barrier is compromised, antibiotics normally reserved for Gram-positives demonstrate antibacterial activity against Gram-negative pathogens.^49^ Interestingly, 1771 and **13** inhibited the growth of *E. coli* and other multidrug-resistant Gram-negative pathogens when combined with the efflux pump inhibitor PAβN. Given the absence of LtaS and LTA production in these organisms, we hypothesized that these compounds could inhibit components of pathways present in both Gram-positive and Gram-negative bacteria that directly feed into the LTA biosynthetic pathway of *S. aureus*. Given the similarity between the phospholipid biosynthetic pathways of both *S. aureus* and *E. coli* and the importance of PG for LTA production, we hypothesize that 1,3,4-oxadiazole-based compounds may exert LTA synthesis inhibitory activity via one of the upstream phospholipid biosynthesis enzymes. An alternative mode of action may involve the inhibiton of trans-translation, the primary ribosome rescue pathway.^50^ Ribosome stalling is frequently observed in bacteria and occurs when ribsosomes stall at the 3′ end of an mRNA that lacks a stop codon or due to damage of the mRNA. Unresolved stalling of ribosomes results in a loss of protein synthesis and bacterial death. Trans-translation is a rescue pathway employing a transfer-mRNA (tmRNA)-SmpB ribonucleoprotein complex that directs the growing polypeptide for degradation and releases the ribosome using a stop codon housed within the tmRNA.^50^ Oxadiazole small-molecule inhibitors have been shown to inhibit trans-translation in *S. aureus*(51) and other bacteria^52,53^ and represent a potential mechanism of activity of 1771 and **13**. Work ongoing in our laboratories is focused on resolving the specific binding partner(s) of 1771 and **13** in both *S. aureus* and *E. coli*.

Although this work questions whether 1771 targets specifically LtaS, small-molecule inhibitors of LtaS represent promising antimicrobial compounds. LtaS exhibits the features of a strong drug target, namely, a homologue is not present in eukaryotes, and inhibition of LtaS abolishes bacterial growth. Furthermore, the enzymatic domain of LtaS is also believed to be displayed and function on the outside of the bacterial membrane, preventing the need for inhibitory molecules to cross the membrane.^54^ LtaS is specific to Gram-positive bacteria; thus, LtaS inhibitors represent narrow-spectrum antimicrobials that have the potential to mitigate the selection and spread of resistance and limit the disruption of the host microbiome.^55^

However, *ltaS* mutants that lack the LTA polymer can be generated in the laboratory at low temperatures and when grown under osmotically stabilizing conditions.^19^ Using a suppressor screen approach, several studies have shown that bacteria can acquire compensatory mutations that permit the growth of *ltaS* mutants under normal conditions and improve the morphological defects associated with LTA deficiency.^30−32^ Although mutations in *ltaS* that disrupt enzymatic activity and LTA production would confer resistance to LtaS inhibitors, resistant mutants would display significant fitness costs associated with the lack of LTA polymer formation and compensatory mutations. LTA plays important roles in directing cell division machinery,^56^ regulating cell size and autolytic activity,^19^ facilitating biofilm formation^57^ and interactions with host cell receptors,^10^ and conferring resistance to bactericidal antimicrobial peptides and fatty acids.^58^ The emergence of resistance would likely result in bacteria that are less able to colonize and cause infection and be more prone to immune elimination. Frequently observed compensatory mutations occur in genes coding for ClpX, GtpP, SgtB, MazE, and VraT, typically resulting in frameshift mutations and introduction of premature stop codons.^30−32^ Inactivation of LtaS in these mutants reduces peptidoglycan cross-linking and increases susceptibility to cell wall acting antibiotics such as vancomycin and oxacillin.^30−32^ Moreover, inactivation of ClpX has been shown to significantly reduce virulence in animal models of infection.^59^ Looking forward, inhibitors of LtaS would be beneficial both as monotherapy or as part of combination therapy, for example, with a WTA inhibitor, as loss of both LTA and WTA leads to synthetic lethality in *S. aureus*.^19^

Our data illustrate for the first time that 1771 (and **13**) can be considered a broad spectrum and are not restricted to LTA producing Gram-positive organisms. Considerable effort is being directed at developing multidrug efflux pump inhibitors for the treatment of Gram-negative infections;^60^ the discovery that 1771 and **13** are active against multidrug-resistant Gram-negative pathogens holds promise for future combinatorial therapy with efflux pump inhibitors. In addition, we and others^12^ have observed limited resistance development against 1771 (Figure S2) and similar compounds^13^ following extensive in vitro serial passage, which may be explained if 1771 and related molecules inhibit multiple targets, preventing the rapid emergence of resistance.

Although compound 1771 and related molecules showed antistaphylococcal activity at therapeutically viable concentrations, the presence of an ester moiety makes this class of compounds susceptible to esterase hydrolysis in blood. Previously, to rule out that 1771 antimicrobial activity could be due to 1771 breakdown products, we examined the hydrolysis pathway of 1771 in serum, identifying the three major metabolites.^21^ We chemically prepared these metabolites and showed that they are not responsible for 1771 antibacterial activity.^21^ Thus, 1771 and compounds developed in this study are still liable to esterase-mediated hydrolysis and currently may not be suitable for the treatment of bloodstream infection. Future work ongoing in our laboratory aims to address this sensitivity as well as to conclusively identify 1,3,4-oxadiazole binding partners to enhance our molecular understanding of this promising class of small-molecule inhibitors.

## 4 Materials and Methods

### 4.1 Chemistry

All commercially available reagents were supplied by Sigma-Aldrich, Fisher, Apollo, Acros Organics, or Fluorochem and used without further purification. 1771 was purchased from Enamine (catalogue number: EN300-97918). Solvents were supplied by Fisher or Acros Organics, anhydrous solvents were supplied by Acros Organics stored over molecular sieves and under nitrogen, and HPLC solvents were supplied by Fisher. For analytical thin-layer chromatography (TLC), precoated aluminum-backed plates (60 F-54, 0.2 mm thickness; supplied by E. Merck AG, Darmstadt, Germany) were used and developed by an ascending elution method. After solvent evaporation, compounds were detected by quenching of fluorescence at 254 nm upon irradiation with a UV lamp and basic KMnO_4_ dip followed by heating until yellow spots appeared. Column chromatography purifications were performed by an automatic Biotage Isolera One or manually using 40–60 μm silica. Fractions containing the product were identified by TLC and pooled, and the solvent was removed in vacuo. ^1^H, ^13^C, and ^19^F NMR spectra were recorded on a Bruker Ascend 500, Bruker Ultrashield 400, or Bruker Fourier 300 spectrometer at 500, 400, and 300 MHz, respectively, for ^1^H NMR; at 125, 100, and 75 MHz for ^13^C NMR; and at 470 and 376 MHz, respectively, for ^19^F NMR. Spectra were calibrated to the deuterated solvent reference peak in ^1^H NMR and ^13^C NMR. All ^13^C NMR spectra were proton-decoupled. Chemical shifts were given in parts per million (ppm), and coupling constants (*J*) were measured in hertz (Hz). The following abbreviations were used in the assignment of NMR signals: s (singlet), d (doublet), dd (doublet of doublets), ddd (doublet of doublet of doublets), td (triplet of doublets), q (quartet), quin (quintet), m (multiplet), and br (broad). Analytical high-performance liquid chromatography (HPLC) analysis was performed using an Agilent 1260 Infinity HPLC system on an Agilent Pursuit C18, 3 × 100 mm, 5 μm column using acetonitrile (ACN) with 0.1% v/v trifluoroacetic acid and water (H_2_O) with 0.1% trifluoroacetic acid in a gradient that is isocratic at 90:10 H_2_O/ACN for 3 min, has a 90:10–0:100 H_2_O/ACN gradient over 30 min, and is isocratic at 0:100 H_2_O/ACN for 2 min at 1 mL/min monitoring at λ = 270 nm at ambient temperature unless stated otherwise. Low- and high-resolution mass spectrometry was performed on a Thermo Scientific Exactive GC orbitrap (chemical ionization) or a Waters Xevo G2XS (electrospray ionization).

### 4.2 Synthesis of 1771 Derivatives

#### 4.2.1 General Procedures

##### 4.2.1.1 General Procedure 1: Synthesis of Coumarins **4a,b**

Ethyl chloroacetoacetate (**2**) (1 equiv) was added to a mixture of concentrated sulfuric acid (2 equiv) and either naphthalen-2-ol (**3a**) or 6-methoxynaphthalen-2-ol (**3b**) (1 equiv) and stirred at 0–5 °C for 24–72 h. The resulting mixture was precipitated in water, filtered, and then dried under high vacuum to afford the products **4a,b**.

##### 4.2.1.2 General Procedure 2: Synthesis of Naphthylfuran Acetic Acids **5a,b**

Compounds **4a,b** (1 equiv) were suspended in a 1 M aqueous sodium hydroxide solution (2.5 equiv), and the mixture was heated to 80 °C at reflux for 4 h. The resulting solution was then allowed to cool to room temperature and was acidified using concentrated hydrochloric acid to pH 2. The resulting precipitate was filtered and then dried under high vacuum to afford the desired products **5a,b**.

##### 4.2.1.3 General Procedure 3: Synthesis of 5-Aryl-2-amino-1,3,4-oxadiazoles **7a–d**

First step: a solution of an appropriate benzaldehyde **6a–d** (1 equiv) in methanol was added to a solution of semicarbazide hydrochloride (1 equiv) and sodium acetate (1 equiv) in water, a white precipitate usually formed within seconds of addition. After stirring for 20 min in room temperature, the precipitate was filtered and washed with ether and then dried under a vacuum pump to afford the desired semicarbazone. Second step: To the semicarbazone were added anhydrous K_2_CO_3_ (3–4.5 equiv) and iodine (1.2 equiv) and then dissolved in anhydrous 1,4-dioxane. The reaction mixture became a purple-brown suspension and was stirred for 16–20 h at 80–95 °C under nitrogen. The mixture turned into a light brown suspension. After cooling to ambient temperature, the product was treated with 5% Na_2_S_2_O_3_ (w/v) and extracted with a mixture of dichloromethane and methanol (9:1). The combined organic layers were dried over anhydrous Mg_2_SO_4_ and concentrated. The crude material was then purified via silica gel column chromatography using a gradient of methanol in dichloromethane, and the product containing fractions were pooled, evaporated, and dried under high vacuum to afford the desired products **7a–d**.

##### 4.2.1.4 General Procedure 4: Synthesis of 2-Chloro-*N*-(5-(aryl)-1,3,4-oxadiazol-2-yl)-acetamides **8a–e**

Compounds **7a–e** (1 equiv) were suspended in dry toluene, and chloroacetyl chloride (1.1 equiv) was added. The suspension was then heated to 80 °C for 18 h under an anhydrous calcium chloride guard tube. The resulting mixture was cooled to room temperature, and then toluene and residual chloroacetyl chloride were evaporated on a rotary evaporator to afford the desired products **8a–e**.

##### 4.2.1.5 General Procedure 5: Synthesis of Compounds **9–14**

Sodium iodide (0.1 equiv) and triethylamine (1.1 equiv) were added to a solution of compounds **5a,b** (1 equiv) and compounds **8a–e** (1 equiv) in anhydrous dimethyl formamide; then the mixture was heated (60–90 °C) for 2–18 h. The crude reaction mixture was worked up by partitioning between ethyl acetate and water, and the organic layer was washed twice with water, then washed with brine dried over magnesium sulfate, and evaporated under vacuum. The crude material was then purified via silica gel column chromatography using a gradient of methanol in dichloromethane; the product containing fraction swere pooled, evaporated, and dried under high vacuum to afford the desired products **9–**–**14** in 4–50% yield.

##### 4.2.1.6 1-(Chloromethyl)-3*H*-benzo[*f*]chromen-3-one (**4a**)

**4a** was prepared according to general procedure 1 from ethyl-4-chloroacetoacetate (**2**) (10.0 g, 8.21 mL, 61 mmol), naphthalen-2-ol (**3a**) (8.8 g, 61 mmol), and concentrated sulfuric acid (6.5 mL, 122 mmol), 24 h, and obtained as a yellow amorphous powder (14.2 g, 96%). *R*_*f*_: 0.31 (30% ethyl acetate in hexanes); ^1^H NMR (500 MHz, DMSO-*d*_6_): 8.55 (d, *J* = 8.9 Hz, 1H), 8.25 (d, *J* = 8.9 Hz 1H), 8.09 (dd, *J* = 8.1 and 1.4 Hz, 1H), 7.78–7.74 (m, 1H), 7.66–7.63 (m, 1H), 7.60 (d, *J* = 8.9 Hz, 1H), 6.88 (s, 1H), 5.41 (s, 2H); ^13^C NMR (125 MHz, DMSO-*d*_6_): 159.17, 154.67, 151.86, 134.42, 130.89, 129.57, 128.39, 128.30, 125.73, 125.49, 117.53, 116.99, 111.89, 46.24.

##### 4.2.1.7 1-(Chloromethyl)-8-methoxy-3*H*-benzo[*f*]chromen-3-one (**4b**)

**4b** was prepared according to general procedure 1 from ethyl 4-chloroacetoacetate (**2**) (0.945 g, 0.77 mL, 5.74 mmol), 6-methoxynaphthylen-2-ol (**3b**) (1.00 g, 5.74 mmol), and concentrated sulfuric acid (0.612 mL, 11.5 mmol), 72 h (1.4 g, 89%). ^1^H NMR (300 MHz, DMSO-*d*_6_): 8.45 (1H, d, *J* = 9.5 Hz), 8.16 (1H, d, *J* = 9.0 Hz), 7.57 (1H, d, *J* = 8.8 Hz), 7.55 (1H, s), 7.39 (1H, dd, *J* = 9.5, 2.9 Hz), 6.85 (1H, s), 5.38 (2H, s), 3.92 (3H, s); ^13^C NMR (101 MHz, DMSO-*d*_6_): 159.24, 156.63, 153.25, 151.65, 133.33, 132.70, 127.02, 122.93, 119.63, 117.86, 117.05, 112.07, 108.63, 55.32, 46.24.

##### 4.2.1.8 2-(Naphtho[2,1-*b*]furan-1-yl)acetic Acid (**5a**)

**5a** was prepared according to general procedure 2 from **4a** (2.2 g, 9.0 mmol) and 1 M sodium hydroxide solution (25 mL) and obtained as an amber amorphous solid (1.9 g, 93%). TLC [dichloromethane]: *R*_*f*_: 0.5; ^1^H NMR (300 MHz, CDCl_3_) 8.13 (1H, d, *J* = 8.3 Hz), 7.82 (1H, d, *J* = 8.1 Hz), 7.51 (1H, dd, *J* = 8.9, 1.8 Hz), 7.45 (1H, ddd, *J* = 8.4, 7.0, 1.4 Hz), 7.35 (1H, ddd, *J* = 8.1, 7.1, 1.2 Hz), 3.92 (2H, s); ^13^C NMR (75 MHz, CDCl_3_) 177.16, 153.57, 143.07, 130.89, 129.29, 128.38, 126.70, 126.18, 124.49, 122.91, 120.94, 114.16, 112.84, 31.59.

##### 4.2.1.9 2-(7-Methoxynaphtho[2,1-*b*]furan-1-yl)acetic Acid (**5b**)

**5b** was prepared according to general procedure 2 from **4b** (1.3 g, 4.7 mmol) and 1 M aqueous NaOH (25 mL) and obtained as a brown amorphous solid (1.0 g, 43%). TLC [5% methanol in dichloromethane]: *R*_*f*_: 0.51; ^1^H NMR (400 MHz, DMSO-*d*_6_): 8.45 (1H, d, *J* = 9.5 Hz), 8.16 (1H, d, *J* = 9.0 Hz), 7.60–7.52 (2H, m), 7.39 (1H, dd, *J* = 9.5, 2.9 Hz), 6.85 (2H, s), 5.38 (2H, s), 3.92 (3H, s); ^13^C NMR (101 MHz, DMSO-*d*_6_): 159.24, 156.63, 153.25, 151.65, 133.33, 132.70, 127.40, 127.02, 122.93, 119.63, 117.86, 117.05, 112.07, 108.63, 55.32, 46.24.

##### 4.2.1.10 2-Amino-5-phenyl-1,3,4-oxadiazole (**7a**)

The title compound was prepared according to general procedure 3 using, for the first step, benzaldehyde (**6a**) (1.0 g, 0.97 mL, 9.5 mmol), semicarbazide hydrochloride (1.1 g, 9.5 mmol), sodium acetate (0.78 g, 9.5 mmol), methanol (19 mL), and water (19 mL) and, for the second step, crude semicarbazone, anhydrous K_2_CO_3_ (3.9 g, 28.5 mmol), iodine (2.9 g, 11.4 mmol), and 1,4-dioxane (94 mL) at 95 °C, 3 h. Purification by silica gel column chromatography using a gradient of methanol (2 to 10%) in dichloromethane afforded compound **7a** as a light-yellow powder (0.89 g, 59%). *R*_*f*_: 0.32 (4% methanol in dichloromethane); ^1^H NMR (500 MHz, DMSO-*d*_6_): δ_H_ 7.81–7.80 (m, 2H), 7.56–7.50 (m, 3H), 7.25 (br, 2H); ^13^C NMR (125 MHz, DMSO-*d*_6_): δ_C_ 164.35, 157.80, 130.82, 129.69, 125.49, 124.87.

##### 4.2.1.11 5-(3-Chlorophenyl)-1,3,4-oxadiazol-2-amine (**7b**)

The title compound was prepared according to general procedure 3 using for the first step 3-chlorobenzaldehyde (**6b**) (1.0 g, 7.1 mmol), semicarbazide hydrochloride (0.81 g, 7.1 mmol), sodium acetate (0.58 g, 7.1 mmol), methanol (10 mL), and water (10 mL) and, for the second step, semicarbazone, anhydrous K_2_CO_3_ (4.4 g, 31.9 mmol), iodine (2.2 g, 8.63 mmol), and anhydrous 1,4-dioxane (30 mL) at 80 °C for 18 h. Purification by silica gel column chromatography using a gradient of methanol (2 to 10%) in dichloromethane afforded compound **7b** that was collected as an off-white amorphous solid (0.85 g, 60%). ^1^H NMR (400 MHz, DMSO-*d*_6_): 7.85–7.68 (1H, m), 7.68–7.49 (1H, m), 7.36 (1H, s); ^13^C NMR (75 MHz, DMSO-*d*_6_): 164.19, 156.34, 134.01, 131.36, 130.23, 126.38, 124.61, 123.72.

##### 4.2.1.12 5-(4-(Fluoro)phenyl)-1,3,4-oxadiazol-2-amine (**7c**)

The title compound was prepared according to general procedure 3 using, for the first step, 4-fluorobenzaldehyde (**6c**) (0.25 g, 0.22 mL, 2.00 mmol), semicarbazide hydrochloride (0.25 g, 2.23 mmol), sodium acetate (0.17 g, 2.05 mmol), methanol (2 mL), and water (2 mL) and, for the second step, semicarbazone, anhydrous K_2_CO_3_ (0.57 g, 4.16 mmol), iodine (0.43 g, 1.70 mmol), and anhydrous 1,4-dioxane (4 mL) at 80 °C for 18 h. Purification by silica gel column chromatography using a gradient of methanol (3 to 5%) in dichloromethane afforded compound **7c** that was collected as an off-white amorphous solid (110 mg, 44%). ^1^H NMR (300 MHz, DMSO-*d*_6_): 7.83 (1H, dd, *J* = 8.8, 5.4 Hz), 7.37 (1H, t, *J* = 8.9 Hz), 7.27 (1H, s); ^19^F NMR (376 MHz, DMSO-*d*_6_): δ −109.86; ^13^C NMR (125 MHz, DMSO-*d*_6_): 163.91, 163.12 (d, *J* = 248.2 Hz), 156.62, 127.52 (d *J* = 8.8 Hz), 121.10 (d *J* = 3.1 Hz), 116.43 (d *J* = 22.4 Hz).

##### 4.2.1.13 5-(4-(Pentafluorosulfanyl) phenyl)-1,3,4-oxadiazol-2-amine (**7d**)

The title compound was prepared according to general procedure 3 using, for the first step, 4-(pentafluorosulfanyl) benzaldehyde (**6d**) (1.00 g, 4.30 mmol), semicarbazide hydrochloride (0.48 g, 4.30 mmol), sodium acetate (0.35 g, 4.30 mmol), methanol (10 mL), and water (10 mL) and, for the second step, semicarbazone, anhydrous K_2_CO_3_ (2.70 g, 19.35 mmol), iodine (1.30 g, 5.16 mmol), and anhydrous 1,4 dioxane (50 mL) at 95 °C for 20 h. Purification by silica gel column chromatography using a gradient of methanol (3 to 5%) in dichloromethane afforded compound **7d** as a yellow amorphous solid (0.45 g, 36%); ^1^H NMR (400 MHz, DMSO-*d*_6_): 8.07 (2H, d, *J* = 8.9 Hz), 7.98 (2H, d, *J* = 8.6 Hz), 7.48 (2H, s); ^19^F NMR (376 MHz, DMSO-*d*_6_): 86.89 (quin, *J* = 150.4 Hz, 1F), 63.91 (d, *J* = 150.4 Hz, 4F): δ_C_ (126 MHz, DMSO): 164.52 (s), 155.99 (s), 153.42–153.25 (m), 127.85 (s), 127.09–126.97 (m), 125.80 (s).

##### 4.2.1.14 5-Phenyl-1,3,4-thiadiazol-2-amine (**7e**)

This compound is commercially available from Sigma-Aldrich (CAS number: 2002-03-1).

##### 4.2.1.15 2-Chloro-*N*-(5-phenyl-1,3,4-oxadiazol-2-yl)acetamide (**8a**)

**8a** was prepared according to general procedure 4 from **7a** (1.61 g, 10.00 mmol) and chloroacetyl chloride (1.24 g, 0.85 mL, 11.00 mmol) in anhydrous toluene (20 mL) and obtained as an off-white amorphous solid (2.25 g, 95%).TLC [10% v/v methanol in dichloromethane] *R*_*f*_: 0.58; ^1^H NMR (300 MHz, DMSO-*d*_6_): 12.26 (1H, s), 7.99–7.87 (3H, m), 7.68–7.54 (5H, m), 4.46 (3H, s); ^13^C NMR (75 MHz, DMSO-*d*_6_): 160.63, 157.02, 131.77, 129.48, 126.04, 123.29.

##### 4.2.1.16 2-Chloro-*N*-(5-(3-chlorophenyl)-1,3,4-oxadiazol-2-yl)acetamide (**8b**)

**8b** was prepared according to general procedure 4 from **7b** (0.50 g, 2.54 mmol) and chloroacetyl chloride (0.34 g, 0.24 mL, 3.01 mmol) in anhydrous toluene (10 mL) and obtained as a cream amorphous powder (0.54 g, 78%). ^1^H NMR (400 MHz, DMSO-*d*_6_): 12.35 (1H, s), 7.95–7.79 (2H, m), 7.71–7.66 (1H, m), 7.66–7.54 (1H, m), 4.46 (1H, s); ^13^C NMR (75 MHz, DMSO-*d*_6_): 164.45, 159.40, 157.27, 134.05, 131.55, 125.46, 125.21, 124.69, 43.15.

##### 4.2.1.17 2-Chloro-*N*-(5-(4-fluorophenyl)-1,3,4-oxadiazol-2-yl)-acetamide (**8c**)

**8c** was prepared according to general procedure 4 from **7c** (0.60 g, 3.35 mmol) and chloroacetyl chloride (0.79 g, 0.56 mL, 7.04 mmol) in anhydrous toluene (1 mL) and obtained as a yellow amorphous solid (0.82 g, 96%). ^1^H NMR (400 MHz, DMSO-*d*_6_): 12.25 (1H, s), 8.01–7.94 (2H, m), 7.49–7.40 (2H, m), 4.45 (2H, s); ^9^F NMR (376 MHz, DMSO-*d*_6_): −107.63; ^13^C NMR (100 MHz, DMSO-*d*_6_): 168.59, 164.40 (d *J* = 250.0 Hz), 156.99, 129.18 (d *J* = 9.1 Hz), 120.45 (d *J* = 3.1 Hz), 117.21 (d *J* = 22.5 Hz), 43.07.

##### 4.2.1.18 2-Chloro-*N*-(5-(4-(pentafluorosulfanyl) phenyl)-1,3,4-oxadiazol-2-yl)-acetamide (**8d**)

**8d** was prepared according to general procedure 4 from **7d** (0.45 g, 1.57 mmol) and chloroacetyl chloride (0.37 g, 0.26 mL, 3.30 mmol) in anhydrous toluene (7 mL) and obtained as a yellow amorphous solid (0.55 g, 96%). ^1^H NMR (400 MHz, DMSO-*d*_6_): 12.44 (1H, s), 8.17 (2H, d, *J* = 9.2 Hz), 8.13 (2H, d, *J* = 9.0 Hz), 4.48 (2H, s); ^19^F NMR (376 MHz, DMSO-*d*_6_): 86.33 (q, *J* = 150.4 Hz, 1F), 63.73 (d, *J* = 150.4 Hz, 4F): δ_C_ (75 MHz, DMSO) 164.86 (s), 159.50 (s), 158.08 (s), 155.16–154.44 (m), 127.88–127.14 (m), 43.59 (s).

##### 4.2.1.19 2-Chloro-*N*-(5-phenyl-1,3,4-thiadiazol-2-yl)acetamide (**8e**)

**8e** was prepared according to general procedure 4 from **7e** (0.10 g, 0.56 mmol) and chloroacetyl chloride (0.07 g, 0.06 mL, 0.62 mmol) in anhydrous toluene (5 mL) and obtained as a white amorphous powder (0.133 g, 93%); ^1^H NMR (300 MHz, DMSO-*d*_6_): 13.08 (1H, s), 7.99–7.91 (2H, m), 7.58–7.50 (3H, m), 4.49 (2H, s); ^13^C NMR (75 MHz, DMSO-*d*_6_): 165.51, 162.34, 158.23, 130.80, 130.01, 129.43, 127.03, 42.39.

##### 4.2.1.20 2-Oxo-2-((5-phenyl-1,3,4-oxadiazol-2-yl)amino)ethyl 2-(Naphtho[2,1-*b*]furan-1-yl)acetate (1771)

The title compound was prepared according to general procedure 5 from **8a** (0.30 g, 1.30 mmol), **5a** (0.34 g, 1.40 mmol), sodium iodide (0.02 g, 0.13 mmol), triethylamine (0.14 g, 0.20 mL, 1.40 mmol), and dimethylformamide (1.4 mL) at 90 °C for 3 h. Purification by silica gel column chromatography afforded compound **1771** (0.31 g, 50%). TLC (3% methanol in dichloromethane) *R*_*f*_: 0.23, ^1^H NMR (500 MHz, CDCl_3_): 8.19 (d, *J* = 8.2 Hz, 1H), 7.93–7.88 (m, 3H), 7.81 (s, 1H), 7.69–7.67 (m, 1H), 7.60–7.39 (m, 6H), 4.92 (s, 2H), 4.26 (s, 2H). ^13^C NMR (125 MHz, DMSO-*d*_6_): 176.15, 170.40, 160.70, 157.22, 152.67, 143.85, 131.70, 130.31, 129.46, 128.88, 127.76, 126.58, 125.97, 125.79, 124.42, 123.33, 120.87, 114.71, 112.64, 62.88, 30.35. Reverse-phase HPLC, eluting with H_2_O/ACN 40:60 for 25 min; to 0:100 in 5 min, flow = 1 mL/min, λ = 263 nm, *t*_R_ = 3.94 min (95%). HRMS *m*/*z*: calcd 428.1249 (M + H)^+^; found, 428.1246 (M + H)^+^.

##### 4.2.1.21 2-Oxo-2-((5-phenyl-1,3,4-oxadiazol-2-yl)amino)ethyl 2-(7-Methoxynaphtho[2,1-*b*]furan-1-yl)acetate (**9**)

The title compound was prepared according to general procedure 5 from **8a** (0.100 g, 0.395 mmol), **5b** (0.089 g, 0.395 mmol), sodium iodide (0.006 g, 0.040 mmol), triethylamine (0.044 g, 0.060 mL, 0.435 mmol), and dimethylformamide (2 mL) at 80 °C for 2 h. Purification by silica gel column chromatography using a gradient of methanol (2 to 10%) in dichloromethane afforded compound **9** (0.013 g, 6% yield). ^1^H NMR (300 MHz, CDCl_3_) 8.10 (1H, d, *J* = 9.1 Hz), 7.97 (2H, d, *J* = 7.0 Hz), 7.77 (1H, s), 7.60 (2H, s), 7.56–7.44 (3H, m), 7.22 (2H, d, *J* = 8.3 Hz), 4.91 (2H, s), 4.22 (2H, s), 3.85 (3H, s). ^13^C NMR (75 MHz, CDCl_3_): 170.20, 156.43, 152.42, 143.10, 132.11, 129.17, 126.74, 124.97, 124.34, 123.07, 121.13, 118.38, 116.65, 113.87, 113.10, 108.18, 55.34, 31.33. HPLC *t*_R_ = 16.6 min (51%). HRMS *m*/*z*: calcd 458.1352 (M + H)^+^; found, 458.1350 (M + H)^+^.

##### 4.2.1.22 2-((5-(3-Chlorophenyl)-1,3,4-oxadiazol-2-yl)amino)-2-oxoethyl 2-(Naphtho[2,1-*b*]furan-1-yl)acetate (**10**)

The title compound was prepared according to general procedure 5 from **5a** (0.081 g, 0.36 mmol), **8b** (0.100 g, 0.400 mmol), sodium iodide (0.005 g, 0.035 mmol), triethylamine (0.040 g, 0.050 mL, 0.400 mmol), and dimethylformamide (1 mL), at 90 °C for 3 h. Purification by silica gel column chromatography using a gradient of methanol (2 to 10%) in dichloromethane afforded compound **10** (6.0 mg, 5% yield). ^1^H NMR (400 MHz, acetone-*d*_6_) 8.36 (1H, d, *J* = 8.4 Hz), 8.07–7.99 (2H, m), 7.98–7.90 (2H, m), 7.84 (1H, d, *J* = 8.7 Hz), 7.71 (1H, d, *J* = 8.9 Hz), 7.68–7.59 (3H, m), 7.50 (1H, t, *J* 7.4), 5.07 (2H, s), 4.36 (2H, s). ^13^C NMR (100 MHz, acetone-*d*_6_) 170.98, 154.19, 144.43, 135.56, 132.29, 132.04, 131.78, 129.81, 129.32, 127.44, 126.79, 126.65, 125.51, 125.24, 124.39, 122.11, 115.93, 113.32. HPLC *t*_R_ = 21.1 min (79%). HRMS *m*/*z*: calcd 462.0857 (M + H)^+^; found, 462.0866 (M + H)^+^.

##### 4.2.1.23 2-((5-(3-Chlorophenyl)-1,3,4-oxadiazol-2-yl)amino)-2-oxoethyl 2-(7-Methoxynaphtho[2,1-*b*]furan-1-yl)acetate (**11**)

The title compound was prepared according to general procedure 5 from **8b** (0.090 g, 0.320 mmol), **5b** (0.080 g, 0.292 mmol), sodium iodide (0.004 g, 0.029 mmol), dimethyl formamide (1 mL), and triethylamine (0.0320 g, 0.040 mL, 0.320 mmol) at 90 °C for 3 h. Purification by silica gel column chromatography using a gradient of methanol (2 to 10%) in dichloromethane afforded compound **11** (0.011 g, 7% yield). ^1^H NMR (400 MHz, DMSO-*d*_6_): 8.14 (1H, d, *J* = 9.1 Hz), 8.06 (1H, s), 7.88 (1H, d, *J* = 1.4 Hz), 7.75 (2H, d, *J* = 2.6 Hz), 7.71–7.67 (1H, m), 7.64–7.60 (1H, m), 7.49 (1H, d, *J* = 2.6 Hz), 7.25 (1H, dd, *J* = 9.1, 2.6 Hz), 7.07–7.03 (1H, m), 4.92 (2H, s), 4.29 (2H, s), 3.87 (3H, s). LRMS *m*/*z*: calcd 492.09 (M + H)^+^; found, 492.10 (M + H)^+^, 514.08 (M + Na)^+^.

##### 4.2.1.24 2-((5-(4-Fluorophenyl)-1,3,4-oxadiazol-2-yl)amino)-2-oxoethyl 2-(Naphtho[2,1-*b*]furan-1-yl)acetate (**12**)

The title compound was prepared according to general procedure 5 from **8c** (0.270 g, 1.07 mmol), **5a** (0.022 g, 0.970 mmol), sodium iodide (0.014 g, 0.097 mmol), triethylamine (0.110 g, 0.150 mL, 1.07 mmol), and dimethylformamide (5 mL) at 90 °C for 3 h. Purification by silica gel column chromatography using a gradient of methanol (2 to 10%) in dichloromethane afforded compound **12** (0.11 g, 0.25 mmol, 25% yield). ^1^H NMR (400 MHz, acetone-*d*_6_) 8.36 (1H, d, *J* = 8.3 Hz), 8.08–7.99 (4H, m), 7.84 (1H, d, *J* = 9.0 Hz), 7.72 (1H, d, *J* = 9.0 Hz), 7.63 (1H, ddd, *J* = 8.2, 7.0, 1.0 Hz), 7.50 (1H, ddd, *J* = 7.9, 6.8, 0.8 Hz), 7.38 (2H, t, *J* = 8.8 Hz), 5.07 (2H, s), 4.36 (2H, s); ^19^F NMR (376 MHz, acetone-*d*_6_) −109.38; ^13^C NMR (101 MHz, acetone-*d*_6_) 170.97, 165.45 (d, *J* = 250.5 Hz), 154.19, 144.42, 131.78, 129.82, 129.63 (d, *J* = 9.0 Hz), 129.32, 127.45, 126.80, 125.24, 124.37, 122.10, 121.43 (d, *J* = 2.6 Hz), 117.30 (d, *J* = 22.6 Hz), 115.93, 113.33, 63.83. HPLC *t*_R_ = 20.0 min (96%). HRMS *m*/*z*: calcd 446.1152 (M + H)^+^; found, 446.1149 (M + H)^+^.

##### 4.2.1.25 2-Oxo-2-((5-(4-(pentafluoro-l6-sulfaneyl)phenyl)-1,3,4-oxadiazol-2-yl)amino)ethyl 2-(Naphtho[2,1-*b*]furan-1-yl)acetate (**13**)

The title compound was prepared according to general procedure 5 from **8d** (0.270 g, 0.729 mmol), **5a** (0.150 g, 0.664 mmol), sodium iodide (0.010 g, 0.067 mmol), triethylamine (0.072 g, 0.100 mL, 0.719 mmol), and dimethylformamide (5 mL) at 90 °C for 5 h. Purification by silica gel column chromatography using a gradient of methanol (2 to 10%) in dichloromethane afforded compound **13** (0.016 g, 4%). ^1^H NMR (400 MHz, CD_3_CN) 8.30–8.25 (1H, m), 8.12–8.04 (2H, m), 8.03–7.95 (3H, m), 7.94–7.89 (1H, m), 7.84–7.78 (1H, m), 7.73–7.67 (1H, m), 7.64–7.59 (1H, m), 7.53–7.47 (1H, m), 4.90 (2H, s), 4.30 (2H, s); ^19^F NMR (376 MHz, CD_3_CN) 82.88 (quin, *J* = 146.7 Hz, 1F) 61.80 (d, *J* = 146.7 Hz, 4F); ^13^C NMR (101 MHz, CD_3_CN) 171.37, 154.24, 144.54, 131.75, 129.95, 129.13, 128.03, 127.98, 127.94, 127.87, 127.61, 126.98, 125.52, 124.36, 121.99, 115.88, 113.56, 64.12. HPLC *t*_R_ = 22.5 min (77%). HRMS *m*/*z*: calcd. 554.0809 (M + H)^+^; found, 554.0810 (M + H)^+^.

##### 4.2.1.26 2-Oxo-2-((5-phenyl-1,3,4-thiadiazol-2-yl)amino)ethyl 2-(Naphtho[2,1-*b*]furan-1-yl)acetate (**14**)

The title compound was prepared according to general procedure 5 from **8e** (0.100 g, 0.395 mmol), **5a** (0.890 g, 0.395 mmol), sodium iodide (0.006 g, 0.040 mmol), triethylamine (0.044 g, 0.060 mL, 0.435 mmol), and dimethylformamide (2 mL) at 80 °C for 2 h. Purification by silica gel column chromatography using a gradient of methanol (2–10%) in dichloromethane afforded compound **13**.

An attempt to purify the mixture by column chromatography (silica, 1% methanol in ethyl acetate + 0.1% concentrated aqueous ammonia) resulted in mixed fractions. A second column (silica, 30% ethyl acetate in hexanes + 0.1% concentrated aqueous ammonia) gave pure fractions, which were combined and reduced in vacuo to give compound **14** as a white amorphous solid (0.016 g; 9%). ^1^H NMR (500 MHz, acetone-*d*_6_) 8.36 (1H, dt, *J* 9.9, 4.9), 8.08–7.97 (4H, m), 7.85 (1H, d, *J* 8.9), 7.72 (1H, d, *J* 8.9), 7.65 (1H, ddd, *J* 8.3, 6.9, 1.3), 7.57–7.48 (4H, m), 5.10 (2H, s), 4.40 (2H, d, *J* 1.0); ^13^C NMR (126 MHz, acetone-*d*_6_) 171.06, 154.19, 144.41, 131.79, 131.66, 131.45, 130.34, 130.13, 129.83, 129.32, 127.95, 127.92, 127.47, 126.81, 125.27, 124.37, 122.10, 115.91, 113.33, 63.46, 32.64, 31.48, 23.33, 14.36. HPLC *t*_R_ = 21.6 min (78%). HRMS *m*/*z*: calcd 444.1018 (M + H)^+^; found, 444.1017 (M + H)^+^.

### 4.3 Bacterial Strains and Culture Conditions

Bacterial strains are listed in Table S1. *S. aureus*, *S. epidermidis*, *E. faecalis,* and *E. faecium* strains were routinely cultivated on tryptic soy agar (TSA) and grown for 18 h at 37 °C. *S. aureus* single colonies were subsequently transferred into 2 mL of tryptic soy broth (TSB) and grown for a further 18 h at 37 °C while being shaken at 180 rpm. *S. pyogenes*, *S. agalactiae,* and *S. dysgalactiae* strains were routinely cultured on TSA supplemented with 5% sheep blood and grown for 18 h at 37 °C with 5% CO_2_. *E. coli* was grown in LB broth at 37 °C. For LtaS-deficient strains, TSB was supplemented with 0.5 M NaCl to provide an osmotically balanced environment and instead grown at 30 °C. For strains harboring the pRMC2 tetracycline inducible vector,^61^ the growth medium was supplemented with 10 μg/mL chloramphenicol and 50–100 ng/μL anhydrotetracycline hydrochloride (Sigma). Phenylalanine-arginine β-naphthylamide (PAβN) (Sigma) was dissolved in sterile water.

### 4.4 Determination of the Minimum Inhibitory Concentration

The MIC of 1771 and its derivatives **9–14** were determined using the microbutter dilution method according to the Clinical and Laboratory Standards Institute (CLSI) guidelines. *B. subtilis*, staphylococcal, and enterococcal strains were grown in Mueller Hinton broth (MHB), while streptococcal strains were grown in MHB supplemented with 5% laked horse blood. All strains were grown for 18 h at 37 °C. The overnight cultures were subsequently subcultured 1:200 and grown to the exponential phase (OD_600nm_ = 0.5–0.6) in fresh broth. 1771 and its derivatives **9–14** were reconstituted in DMSO and further diluted by performing a 1:2 serial dilution ranging between 256 and 0.125 μg/mL in a 96-well microtiter plate. One hundred microliters of 0.5 McFarland standardized inoculum (approximately equal to 5 × 10^5^ cfu) were then dispensed into each well in the 96-well microtiter plate. The plate was incubated for 18 h at 37 °C. The MIC was determined as the lowest concentration that completely inhibited the growth of the bacteria.

### 4.5 Determination of the Minimum Bactericidal Concentration

The MBC was considered the concentration that completely prevented growth and reduced the inoculum sized by ≥99.9%, equal to a >3 log reduction in cfu/mL. For an antibiotic with an MBC ≤ 4×, the MIC was considered to be bactericidal, whereas with an MBC > 4×, the MIC was considered to be bacteriostatic.^24^ Following the incubation of MRSA strain LAC in various concentrations of 1771 and **13** (described above for the determination of MIC), 50 μL of the resulting bacterial suspension was serially diluted 1:10 in 450 μL of PBS and plated out on TSA for enumeration. Percentage survival was calculated according to the starting inoculum of 5 × 10^5^ cfu at time 0 h.

### 4.6 Time-Kill Kinetic Analysis

Overnight cultures of MRSA strain LAC were subcultured 1:200 and grown to the exponential phase in fresh MHB. These cultures were subsequently diluted to give 500 μL of MHB containing 5 × 10^5^ cfu. This inoculum was then combined with 500 μL MHB containing either no drug or 0.25×, 1×, or 4× MIC of either 1771 or **13**. These suspensions were incubated for 24 h at 37 °C, with 50 μL of the suspension serially diluted 1:10 in 450 μL of phosphate buffered saline (PBS) and plated out on TSA at times 0, 1, 2, 4, 6, and 24 h for enumeration.

### 4.7 Biofilm Prevention Assay

An indication of the biofilm prevention capabilities of 1771 and its derivatives **9–14** was achieved through the application of the crystal violet assay. *S. aureus* strain SH1000 and *S. epidermidis* strain RP62A were used, as they are strong biofilm formers. Overnight cultures were diluted 1:200 in fresh TSB containing 0.5% glucose (TSB-G), and 100 μL was added to individual wells in a 96-well microtiter plate. One hundred microliters of 1771 and **9–14** were diluted in TSB-G and added to bacteria at a final concentration ranging from 256 to 0.25 μg/mL with subsequent incubation for 24 h at 37 °C in a static incubator. Following incubation, biofilms were washed four times with ddH_2_O before being stained with 150 μL of 0.1% crystal violet for 30 min at room temperature. Following a further four washes in ddH_2_O, wells were resuspended in 200 μL of 7% acetic acid. The absorbance intensity of the crystal violet was measured at abs595_nm_ in a Sunrise plate reader. Biomass formed was measured as a percentage of the no-compound control.

### 4.8 AsedaSciences SYSTEMETRIC Cell Health Screen

1771 and compounds **9–14** were analyzed over a concentration range from 100 μM to 5 nM using the AsedaSciences SYSTEMETRIC cell health screen. The cell health screen consisted of a multiparametric acute cell stress assay employing a HL-60 cell line, automated flow cytometry, a panel of fluorescent physiological reporting dyes, and machine learning and was previously described in detail.^25,26^ Briefly, the machine learning classifier was trained using a 300-compound training set including on-market and withdrawn drugs and research compounds. Training covered the complete range of possible phenotypes in the cell heath screen from no response to acute toxicity. For an unknown compound, the machine learning classifier combineed all the cell stress parameters describing compound response simultaneously, generating a cell health index (CHI). This is a probability value (between 0 and 1), indicating the maximum likelihood that the unknown compound is grouped within the high-toxicity risk outcome category.

### 4.9 HepG2 Liver Cell Toxicity Assay

HepG2 human liver cancer cells (ECACC: Cat no. 85011430) were grown in Dulbecco’s Modified Eagle’s Medium (DMEM; with 4.5 g/L glucose) supplemented with 10% (v/v) fetal bovine serum, 1% (v/v) minimal essential media, and 0.2% (v/v) penicillin/streptomycin/l-glutamine solution. Cells were then seeded at a concentration of 10,000 cells per well and allowed to attach and grow for 18 h. Following this, the medium was aspirated away and replaced with a medium containing a range of concentrations of 1771 and compound **13,** and the cells were grown for a further 24 h. After this, cell viability was assessed using the MTT (3-[4,5-dimethylthiazol-2-yl]-2,5-diphenyltetrazolium bromide) assay. 1771 or **13** containing growth media was aspirated away and replaced with 100 μL of 1 mg/mL MTT (in growth media) and incubated for 50 min at 37 °C. Subsequently, this solution was removed, and 150 μL of isopropanol was added to the cells and allowed to incubate at RT with gentle rocking on an orbital shaker. Absorbance at abs595_nm_ was recorded using a Sunrise plate reader, and the % cell viability was calculated compared to a no-compound control.

### 4.10 Erythrocyte Toxicity Assay

Consent was collected from three healthy volunteers prior to blood collection and in accordance with the recommendations of the University of Bath, Research Ethics Approval Committee for Health Blood (EP 18/19108). Blood was collected by venepuncture and drawn directly into K_2_-EDTA-coated Vacutainer tubes (BD Biosciences) to prevent coagulation. The blood was then pelleted at 500*g* for 10 min at 4 °C, and the plasma layer was removed. The remaining hematocrit was resuspended to the original volume in a sterile saline solution. This procedure was repeated three times and finally resuspended in sterile PBS and diluted to 2% (v/v). One hundred microlitres of the resulting blood suspension were aliquoted in a 96-well microtire plate. An equal volume of either 1771 or **13** (diluted in PBS) was then added at a concentration range of 256–2 μg/mL and incubated for 1 h at 37 °C. Blood incubated with PBS served as a negative control, and total hemolysis (positive control) was provided by incubating the cells in 2% (v/v) Triton X-100. Following incubation, the 96-well microtire plate was pelleted at 500*g* for 5 min, and 100 μL of supernatant was transferred into a new plate. Absorbance at abs404_nm_ was measured using a Sunrise plate reader, and percentage hemolysis was calculated according to controls.

### 4.11 LTA Quantification by Western Blot

Overnight cultures of *S. aureus* strains were diluted 1:200 in fresh TSB and grown to an optical density of OD_600nm_ 0.3. At this point, either 1× or 4× the MIC of 1771 or compound **13** was added and incubated for a further 2 h at 37 °C. Vancomycin and daptomycin at 1× and 4× MIC were also included as control antibiotics. Cells were then normalized to have 10 mL of OD600_nm_ 0.6, pelleted at 4000 rpm, and resuspended in 300 μL of PBS containing 200 μg/mL lysostaphin. This suspension was then incubated at 37 °C for 45 min to allow digestion of the cell wall to occur. One hundred microlitres of 4× SDS sample buffer were then added, and the mixture was boiled at 95 °C for 20 min. The insoluble material was then removed by pelleting at 16,000 rpm for 10 min. The supernatant containing LTA was then subjected to SDS-PAGE on a 4–20% Tris-glycine gel (Bio-Rad). The gel was transferred onto a PVDF membrane using a Trans-Blot Turbo Transfer System (Bio-Rad). The membrane was blocked overnight in TBST containing 5% semi-skimmed milk before being incubated with a mouse anti-LTA antibody (Hycult; HM2048; 1:2000 dilution) followed by a goat antimouse-HRP secondary antibody (Proteintech; SA00001; 1:2000 dilution) before being visualized using an Amersham-ECL kit (Cytiva).

### 4.12 Genetic Manipulation

For overexpression studies, full-length *ltaS* was amplified by PCR using MRSA strian LAC genomic DNA, primer pair LtaS-FW: 5′-atatggtaccacgcacttattaattaactacataatg-3′ and LtaS-RV: 5′-atatgagctccaatccgagttcgtgtttag-3′, and Phusion High-Fidelity DNA Polymerase (Thermo Fisher). The resulting PCR product was cloned into the tetracycline inducible plasmid pRMC2 using SacI and *Kpn*I restriction sites and T4 ligase (NEB). The cloned vector was subsequently transformed into RN4220 followed by LAC through electroporation.

### 4.13 Docking Method

Docking studies were carried out by means of the software GOLD 2020.1^62^ using the crystal structure of the extracellular domain of LtaS in complex with glycerol-phosphate (PDB ID 2w5s) as 3D coordinates. The protocol used to set up the docking calculation is reported in our previous paper.^21^ Briefly, ligand structures were constructed by the VEGA ZZ program,^63^ and their conformational behavior was explored by a Monte Carlo procedure as implemented in VEGA ZZ. The binding site was defined to include the residues within 10 Å from the native ligand. Each ligand was submitted to 100 genetic algorithm runs using the default settings. ChemPLP was chosen as the scoring function. The protocol was validated by redocking the cocrystallized ligand into the LtaS active site, which leads to the successful reproduction of the X-ray pose with a RMSD value of 0.6493 Å.

### 4.14 Statistical Analysis

All experiments were repeated a minimum of three times using independently grown bacterial cultures. An indication of statistical significance was provided by performing paired two-tailed Student’s *t*-test or one-way analysis of variance (ANOVA) with Dunnett’s multiple comparisons test and single pooled variance. A *p*-value < 0.05 was considered statistically significant.

## Supporting Information Available

List of strains used in the study; cell health index of diverse compounds including clinically relevant antibiotics; checkerboard analysis of 1771 and compound **13** in combination with clinically relevant antibiotics; resistance profile of 1771; molecular modeling of LtaS; LTA expression following induction of LtaS; inhibition of LTA production following treatment with fatty acid inhibitors; permeabilization of the outer membrane, which renders Gram-negative bacteria susceptible to 1771; and NMR spectra and HPLC traces of compounds 9**–**14 and intermediates (PDF)

## References

[ref1] Tong S. Y.; Davis J. S.; Eichenberger E.; Holland T. L.; Fowler V. G. Jr. Staphylococcus aureus infections: epidemiology, pathophysiology, clinical manifestations, and management. Clin. Microbiol. Rev. 2015, 28 (3), 603–661. 10.1128/CMR.00134-14. 26016486 PMC4451395

[ref2] Murray C. J. L.; Ikuta K. S.; Sharara F.; Swetschinski L.; Robles Aguilar G.; Gray A.; Han C.; Bisignano C.; Rao P.; Wool E.; et al. Global burden of bacterial antimicrobial resistance in 2019: a systematic analysis. Lancet 2022, 399 (10325), 629–655. 10.1016/S0140-6736(21)02724-0. 35065702 PMC8841637

[ref3] Howden B. P.; Davies J. K.; Johnson P. D.; Stinear T. P.; Grayson M. L. Reduced vancomycin susceptibility in Staphylococcus aureus, including vancomycin-intermediate and heterogeneous vancomycin-intermediate strains: resistance mechanisms, laboratory detection, and clinical implications. Clin. Microbiol. Rev. 2010, 23 (1), 99–139. 10.1128/CMR.00042-09. 20065327 PMC2806658

[ref4] Unni S.; Siddiqui T. J.; Bidaisee S. Reduced Susceptibility and Resistance to Vancomycin of Staphylococcus aureus: A Review of Global Incidence Patterns and Related Genetic Mechanisms. Cureus 2021, 13 (10), e18925 10.7759/cureus.18925. 34812309 PMC8603868

[ref5] Miller W. R.; Bayer A. S.; Arias C. A. Mechanism of Action and Resistance to Daptomycin in Staphylococcus aureus and Enterococci. Cold Spring Harbor Perspect. Med. 2016, 6 (11), a026997 10.1101/cshperspect.a026997. PMC5088507 27580748

[ref6] Long K. S.; Vester B. Resistance to linezolid caused by modifications at its binding site on the ribosome. Antimicrob. Agents Chemother. 2012, 56 (2), 603–612. 10.1128/AAC.05702-11. 22143525 PMC3264260

[ref7] Morales G.; Picazo J. J.; Baos E.; Candel F. J.; Arribi A.; Pelaez B.; Andrade R.; de la Torre M. A.; Fereres J.; Sanchez-Garcia M. Resistance to linezolid is mediated by the cfr gene in the first report of an outbreak of linezolid-resistant Staphylococcus aureus. Clin. Infect. Dis. 2010, 50 (6), 821–825. 10.1086/650574. 20144045

[ref8] Douglas E. J. A.; Wulandari S. W.; Lovell S. D.; Laabei M. Novel antimicrobial strategies to treat multi-drug resistant Staphylococcus aureus infections. Microb. Biotechnol. 2023, 16 (7), 1456–1474. 10.1111/1751-7915.14268. 37178319 PMC10281381

[ref9] Percy M. G.; Grundling A. Lipoteichoic acid synthesis and function in gram-positive bacteria. Annu. Rev. Microbiol. 2014, 68, 81–100. 10.1146/annurev-micro-091213-112949. 24819367

[ref10] Weidenmaier C.; Peschel A. Teichoic acids and related cell-wall glycopolymers in Gram-positive physiology and host interactions. Nat. Rev. Microbiol. 2008, 6 (4), 276–287. 10.1038/nrmicro1861. 18327271

[ref11] Vickery C. R.; Wood B. M.; Morris H. G.; Losick R.; Walker S. Reconstitution of Staphylococcus aureus Lipoteichoic Acid Synthase Activity Identifies Congo Red as a Selective Inhibitor. J. Am. Chem. Soc. 2018, 140 (3), 876–879. 10.1021/jacs.7b11704. 29300473 PMC5856125

[ref12] Richter S. G.; Elli D.; Kim H. K.; Hendrickx A. P.; Sorg J. A.; Schneewind O.; Missiakas D. Small molecule inhibitor of lipoteichoic acid synthesis is an antibiotic for Gram-positive bacteria. Proc. Natl. Acad. Sci. U.S.A. 2013, 110 (9), 3531–3536. 10.1073/pnas.1217337110. 23401520 PMC3587227

[ref13] Naclerio G. A.; Abutaleb N. S.; Onyedibe K. I.; Karanja C.; Eldesouky H. E.; Liang H. W.; Dieterly A.; Aryal U. K.; Lyle T.; Seleem M. N.; Sintim H. O. Mechanistic Studies and In Vivo Efficacy of an Oxadiazole-Containing Antibiotic. J. Med. Chem. 2022, 65 (9), 6612–6630. 10.1021/acs.jmedchem.1c02034. 35482444 PMC9124606

[ref14] Chee Wezen X.; Chandran A.; Eapen R. S.; Waters E.; Bricio-Moreno L.; Tosi T.; Dolan S.; Millership C.; Kadioglu A.; Grundling A.; Itzhaki L. S.; Welch M.; Rahman T. Structure-Based Discovery of Lipoteichoic Acid Synthase Inhibitors. J. Chem. Inf. Model. 2022, 62 (10), 2586–2599. 10.1021/acs.jcim.2c00300. 35533315 PMC9131456

[ref15] Lu D.; Wormann M. E.; Zhang X.; Schneewind O.; Grundling A.; Freemont P. S. Structure-based mechanism of lipoteichoic acid synthesis by Staphylococcus aureus LtaS. Proc. Natl. Acad. Sci. U.S.A. 2009, 106 (5), 1584–1589. 10.1073/pnas.0809020106. 19168632 PMC2635763

[ref16] Chaudhuri R. R.; Allen A. G.; Owen P. J.; Shalom G.; Stone K.; Harrison M.; Burgis T. A.; Lockyer M.; Garcia-Lara J.; Foster S. J.; Pleasance S. J.; Peters S. E.; Maskell D. J.; Charles I. G. Comprehensive identification of essential Staphylococcus aureus genes using Transposon-Mediated Differential Hybridisation (TMDH). BMC Genomics 2009, 10, 291 10.1186/1471-2164-10-291. 19570206 PMC2721850

[ref17] Fey P. D.; Endres J. L.; Yajjala V. K.; Widhelm T. J.; Boissy R. J.; Bose J. L.; Bayles K. W. A genetic resource for rapid and comprehensive phenotype screening of nonessential Staphylococcus aureus genes. mBio 2013, 4 (1), e00537 10.1128/mBio.00537-12. 23404398 PMC3573662

[ref18] Coe K. A.; Lee W.; Stone M. C.; Komazin-Meredith G.; Meredith T. C.; Grad Y. H.; Walker S. Multi-strain Tn-Seq reveals common daptomycin resistance determinants in Staphylococcus aureus. PLoS Pathog. 2019, 15 (11), e1007862 10.1371/journal.ppat.1007862. 31738809 PMC6934316

[ref19] Oku Y.; Kurokawa K.; Matsuo M.; Yamada S.; Lee B. L.; Sekimizu K. Pleiotropic Roles of Polyglycerolphosphate Synthase of Lipoteichoic Acid in Growth of Staphylococcus aureus Cells. J. Bacteriol. 2009, 191 (1), 141–151. 10.1128/JB.01221-08. 18952789 PMC2612411

[ref20] Naclerio G. A.; Onyedibe K. I.; Sintim H. O. Lipoteichoic Acid Biosynthesis Inhibitors as Potent Inhibitors of S. aureus and E. faecalis Growth and Biofilm Formation. Molecules 2020, 25 (10), 2277 10.3390/molecules25102277. 32408616 PMC7287929

[ref21] Serpi M.; Pertusati F.; Morozzi C.; Novelli G.; Giannantonio D.; Duggan K.; Vittorio S.; Fallis I. A.; De Luca L.; Williams D. Synthesis, molecular docking and antibacterial activity of an oxadiazole-based lipoteichoic acid inhibitor and its metabolites. J. Mol. Struct. 2023, 1278, 134977 10.1016/j.molstruc.2023.134977. PMC10836577 38312219

[ref22] Douglas E. J. A.; Alkhzem A. H.; Wonfor T.; Li S.; Woodman T. J.; Blagbrough I. S.; Laabei M. Antibacterial activity of novel linear polyamines against Staphylococcus aureus. Front. Microbiol. 2022, 13, 948343 10.3389/fmicb.2022.948343. 36071957 PMC9441809

[ref23] Gill S. R.; Fouts D. E.; Archer G. L.; Mongodin E. F.; Deboy R. T.; Ravel J.; Paulsen I. T.; Kolonay J. F.; Brinkac L.; Beanan M.; Dodson R. J.; Daugherty S. C.; Madupu R.; Angiuoli S. V.; Durkin A. S.; Haft D. H.; Vamathevan J.; Khouri H.; Utterback T.; Lee C.; Dimitrov G.; Jiang L.; Qin H.; Weidman J.; Tran K.; Kang K.; Hance I. R.; Nelson K. E.; Fraser C. M. Insights on evolution of virulence and resistance from the complete genome analysis of an early methicillin-resistant Staphylococcus aureus strain and a biofilm-producing methicillin-resistant Staphylococcus epidermidis strain. J. Bacteriol. 2005, 187 (7), 2426–2438. 10.1128/JB.187.7.2426-2438.2005. 15774886 PMC1065214

[ref24] Pankey G. A.; Sabath L. D. Clinical relevance of bacteriostatic versus bactericidal mechanisms of action in the treatment of Gram-positive bacterial infections. Clin. Infect. Dis. 2004, 38 (6), 864–870. 10.1086/381972. 14999632

[ref25] Bieberich A. A.; Rajwa B.; Irvine A.; Fatig R. O. 3rd; Fekete A.; Jin H.; Kutlina E.; Urban L. Acute cell stress screen with supervised machine learning predicts cytotoxicity of excipients. J. Pharmacol. Toxicol. Methods 2021, 111, 107088 10.1016/j.vascn.2021.107088. 34144174

[ref26] Bieberich A. A.; Asquith C. R. M. Utilization of Supervised Machine Learning to Understand Kinase Inhibitor Toxophore Profiles. Int. J. Mol. Sci. 2023, 24 (6), 5088 10.3390/ijms24065088. 36982163 PMC10049021

[ref27] Doß S.; Blessing C.; Haller K.; Richter G.; Sauer M. Influence of Antibiotics on Functionality and Viability of Liver Cells In Vitro. Curr. Issues Mol. Biol. 2022, 44 (10), 4639–4657. 10.3390/cimb44100317. 36286032 PMC9600611

[ref28] Hesser A. R.; Schaefer K.; Lee W.; Walker S. Lipoteichoic acid polymer length is determined by competition between free starter units. Proc. Natl. Acad. Sci. U.S.A. 2020, 117 (47), 29669–29676. 10.1073/pnas.2008929117. 33172991 PMC7703640

[ref29] Jumper J.; Evans R.; Pritzel A.; Green T.; Figurnov M.; Ronneberger O.; Tunyasuvunakool K.; Bates R.; Zidek A.; Potapenko A.; Bridgland A.; Meyer C.; Kohl S. A. A.; Ballard A. J.; Cowie A.; Romera-Paredes B.; Nikolov S.; Jain R.; Adler J.; Back T.; Petersen S.; Reiman D.; Clancy E.; Zielinski M.; Steinegger M.; Pacholska M.; Berghammer T.; Bodenstein S.; Silver D.; Vinyals O.; Senior A. W.; Kavukcuoglu K.; Kohli P.; Hassabis D. Highly accurate protein structure prediction with AlphaFold. Nature 2021, 596 (7873), 583–589. 10.1038/s41586-021-03819-2. 34265844 PMC8371605

[ref30] Baek K. T.; Bowman L.; Millership C.; Dupont Sogaard M.; Kaever V.; Siljamaki P.; Savijoki K.; Varmanen P.; Nyman T. A.; Grundling A.; Frees D. The Cell Wall Polymer Lipoteichoic Acid Becomes Nonessential in Staphylococcus aureus Cells Lacking the ClpX Chaperone. mBio 2016, 7 (4), e01228 10.1128/mBio.01228-16. 27507828 PMC4981727

[ref31] Karinou E.; Schuster C. F.; Pazos M.; Vollmer W.; Grundling A. Inactivation of the Monofunctional Peptidoglycan Glycosyltransferase SgtB Allows Staphylococcus aureus To Survive in the Absence of Lipoteichoic Acid. J. Bacteriol. 2019, 201 (1), e00574 10.1128/JB.00574-18. 30322854 PMC6287468

[ref32] Corrigan R. M.; Abbott J. C.; Burhenne H.; Kaever V.; Grundling A. c-di-AMP is a new second messenger in Staphylococcus aureus with a role in controlling cell size and envelope stress. PLoS Pathog. 2011, 7 (9), e1002217 10.1371/journal.ppat.1002217. 21909268 PMC3164647

[ref33] Santa Maria J. P. Jr.; Sadaka A.; Moussa S. H.; Brown S.; Zhang Y. J.; Rubin E. J.; Gilmore M. S.; Walker S. Compound-gene interaction mapping reveals distinct roles for Staphylococcus aureus teichoic acids. Proc. Natl. Acad. Sci. U.S.A. 2014, 111 (34), 12510–12515. 10.1073/pnas.1404099111. 25104751 PMC4151746

[ref34] Kuhn S.; Slavetinsky C. J.; Peschel A. Synthesis and function of phospholipids in Staphylococcus aureus. Int. J. Med. Microbiol. 2015, 305 (2), 196–202. 10.1016/j.ijmm.2014.12.016. 25595024

[ref35] Heath R. J.; Rubin J. R.; Holland D. R.; Zhang E. L.; Snow M. E.; Rock C. O. Mechanism of triclosan inhibition of bacterial fatty acid synthesis. J. Biol. Chem. 1999, 274 (16), 11110–11114. 10.1074/jbc.274.16.11110. 10196195

[ref36] Park H. S.; Yoon Y. M.; Jung S. J.; Kim C. M.; Kim J. M.; Kwak J. H. Antistaphylococcal activities of CG400549, a new bacterial enoyl-acyl carrier protein reductase (FabI) inhibitor. J. Antimicrob. Chemother. 2007, 60 (3), 568–574. 10.1093/jac/dkm236. 17606482

[ref37] Wesseling C. M. J.; Martin N. I. Synergy by Perturbing the Gram-Negative Outer Membrane: Opening the Door for Gram-Positive Specific Antibiotics. ACS Infect. Dis. 2022, 8, 1731–1757. 10.1021/acsinfecdis.2c00193. 35946799 PMC9469101

[ref38] Li X. Z.; Zhang L.; Poole K. Interplay between the MexA-MexB-OprM multidrug efflux system and the outer membrane barrier in the multiple antibiotic resistance of Pseudomonas aeruginosa. J. Antimicrob. Chemother. 2000, 45 (4), 433–436. 10.1093/jac/45.4.433. 10747818

[ref39] Parker E. N.; Drown B. S.; Geddes E. J.; Lee H. Y.; Ismail N.; Lau G. W.; Hergenrother P. J. Implementation of permeation rules leads to a FabI inhibitor with activity against Gram-negative pathogens. Int. Microbiol. 2019, 5 (1), 67–75. 10.1038/s41564-019-0604-5. PMC6953607 31740764

[ref40] Lamers R. P.; Cavallari J. F.; Burrows L. L. The Efflux Inhibitor Phenylalanine-Arginine Beta-Naphthylamide (PAβN) Permeabilizes the Outer Membrane of Gram-Negative Bacteria. PLoS One 2013, 8 (3), e60666 10.1371/journal.pone.0060666. 23544160 PMC3609863

[ref41] Gillis E. P.; Eastman K. J.; Hill M. D.; Donnelly D. J.; Meanwell N. A. Applications of Fluorine in Medicinal Chemistry. J. Med. Chem. 2015, 58 (21), 8315–8359. 10.1021/acs.jmedchem.5b00258. 26200936

[ref42] Pertusati F.; Serpi M.; Pileggi E. 3—Polyfluorinated scaffolds in drug discovery. In Fluorine in Life Sciences: Pharmaceuticals, Medicinal Diagnostics, and Agrochemicals; Haufe G., Leroux F. R., Eds.; Academic Press, 2019; pp 141–180.

[ref43] Naclerio G. A.; Abutaleb N. S.; Li D.; Seleem M. N.; Sintim H. O. Ultrapotent Inhibitor of Clostridioides difficile Growth, Which Suppresses Recurrence In Vivo. J. Med. Chem. 2020, 63 (20), 11934–11944. 10.1021/acs.jmedchem.0c01198. 32960605 PMC9064041

[ref44] Naclerio G. A.; Abutaleb N. S.; Onyedibe K. I.; Seleem M. N.; Sintim H. O. Potent trifluoromethoxy, trifluoromethylsulfonyl, trifluoromethylthio and pentafluorosulfanyl containing (1,3,4-oxadiazol-2-yl)benzamides against drug-resistant Gram-positive bacteria. RSC Med. Chem. 2020, 11 (1), 102–110. 10.1039/C9MD00391F. 33479609 PMC7536820

[ref45] Sowaileh M. F.; Hazlitt R. A.; Colby D. A. Application of the Pentafluorosulfanyl Group as a Bioisosteric Replacement. Chemmedchem 2017, 12 (18), 1481–1490. 10.1002/cmdc.201700356. 28782186

[ref46] Sun A. W.; Bulterys P. L.; Bartberger M. D.; Jorth P. A.; O’Boyle B. M.; Virgil S. C.; Miller J. F.; Stoltz B. M. Incorporation of a chiral gem-disubstituted nitrogen heterocycle yields an oxazolidinone antibiotic with reduced mitochondrial toxicity. Bioorg. Med. Chem. Lett. 2019, 29 (18), 2686–2689. 10.1016/j.bmcl.2019.07.024. 31383589 PMC6711789

[ref47] Paganelli F. L.; van de Kamer T.; Brouwer E. C.; Leavis H. L.; Woodford N.; Bonten M. J.; Willems R. J.; Hendrickx A. P. Lipoteichoic acid synthesis inhibition in combination with antibiotics abrogates growth of multidrug-resistant Enterococcus faecium. Int. J. Antimicrob. Agents 2017, 49 (3), 355–363. 10.1016/j.ijantimicag.2016.12.002. 28188831

[ref48] Delcour A. H. Outer membrane permeability and antibiotic resistance. BBA, Biochim. Biophys. Acta, Proteins Proteomics 2009, 1794 (5), 808–816. 10.1016/j.bbapap.2008.11.005. PMC2696358 19100346

[ref49] Randall C. P.; Mariner K. R.; Chopra I.; O’Neill A. J. The Target of Daptomycin Is Absent from Escherichia coli and Other Gram-Negative Pathogens. Antimicrob. Agents Chemother. 2013, 57 (1), 637–639. 10.1128/AAC.02005-12. 23114759 PMC3535891

[ref50] Keiler K. C. Mechanisms of ribosome rescue in bacteria. Nat. Rev. Microbiol. 2015, 13 (5), 285–297. 10.1038/nrmicro3438. 25874843

[ref51] Huang Y.; Alumasa J. N.; Callaghan L. T.; Baugh R. S.; Rae C. D.; Keiler K. C.; McGillivray S. M. A Small-Molecule Inhibitor of trans-Translation Synergistically Interacts with Cathelicidin Antimicrobial Peptides To Impair Survival of Staphylococcus aureus. Antimicrob. Agents Chemother. 2019, 63 (4), e02362 10.1128/AAC.02362-18. 30917982 PMC6437501

[ref52] Ramadoss N. S.; Alumasa J. N.; Cheng L.; Wang Y.; Li S.; Chambers B. S.; Chang H.; Chatterjee A. K.; Brinker A.; Engels I. H.; Keiler K. C. Small molecule inhibitors of trans-translation have broad-spectrum antibiotic activity. Proc. Natl. Acad. Sci. U.S.A. 2013, 110 (25), 10282–10287. 10.1073/pnas.1302816110. 23733947 PMC3690859

[ref53] Alumasa J. N.; Manzanillo P. S.; Peterson N. D.; Lundrigan T.; Baughn A. D.; Cox J. S.; Keiler K. C. Ribosome Rescue Inhibitors Kill Actively Growing and Nonreplicating Persister Mycobacterium tuberculosis Cells. ACS Infect. Dis. 2017, 3 (9), 634–644. 10.1021/acsinfecdis.7b00028. 28762275 PMC5594445

[ref54] Grundling A.; Schneewind O. Synthesis of glycerol phosphate lipoteichoic acid in Staphylococcus aureus. Proc. Natl. Acad. Sci. U.S.A. 2007, 104 (20), 8478–8483. 10.1073/pnas.0701821104. 17483484 PMC1895975

[ref55] Melander R. J.; Zurawski D. V.; Melander C. Narrow-Spectrum Antibacterial Agents. Medchemcomm 2018, 9 (1), 12–21. 10.1039/C7MD00528H. 29527285 PMC5839511

[ref56] Schirner K.; Marles-Wright J.; Lewis R. J.; Errington J. Distinct and essential morphogenic functions for wall- and lipo-teichoic acids in Bacillus subtilis. EMBO J. 2009, 28 (7), 830–842. 10.1038/emboj.2009.25. 19229300 PMC2670855

[ref57] Fedtke I.; Mader D.; Kohler T.; Moll H.; Nicholson G.; Biswas R.; Henseler K.; Gotz F.; Zahringer U.; Peschel A. A Staphylococcus aureus ypfP mutant with strongly reduced lipoteichoic acid (LTA) content: LTA governs bacterial surface properties and autolysin activity. Mol. Microbiol. 2007, 65 (4), 1078–1091. 10.1111/j.1365-2958.2007.05854.x. 17640274 PMC2169524

[ref58] Neuhaus F. C.; Baddiley J. A continuum of anionic charge: structures and functions of D-alanyl-teichoic acids in gram-positive bacteria. Microbiol. Mol. Biol. Rev. 2003, 67 (4), 686–723. 10.1128/MMBR.67.4.686-723.2003. 14665680 PMC309049

[ref59] Frees D.; Qazi S. N.; Hill P. J.; Ingmer H. Alternative roles of ClpX and ClpP in Staphylococcus aureus stress tolerance and virulence. Mol. Microbiol. 2003, 48 (6), 1565–1578. 10.1046/j.1365-2958.2003.03524.x. 12791139

[ref60] Blanco P.; Sanz-Garcia F.; Hernando-Amado S.; Martinez J. L.; Alcalde-Rico M. The development of efflux pump inhibitors to treat Gram-negative infections. Expert Opin. Drug Discovery 2018, 13 (10), 919–931. 10.1080/17460441.2018.1514386. 30198793

[ref61] Corrigan R. M.; Foster T. J. An improved tetracycline-inducible expression vector for Staphylococcus aureus. Plasmid 2009, 61 (2), 126–129. 10.1016/j.plasmid.2008.10.001. 18996145

[ref62] Jones G.; Willett P.; Glen R. C.; Leach A. R.; Taylor R. Development and validation of a genetic algorithm for flexible docking. J. Mol. Biol. 1997, 267 (3), 727–748. 10.1006/jmbi.1996.0897. 9126849

[ref63] Pedretti A.; Mazzolari A.; Gervasoni S.; Fumagalli L.; Vistoli G. The VEGA suite of programs: an versatile platform for cheminformatics and drug design projects. Bioinformatics 2021, 37 (8), 1174–1175. 10.1093/bioinformatics/btaa774. 33289523

