# Supplementary material for: Improved Antibacterial Activity of 1,3,4-Oxadiazole-Based Compounds That Restrict Staphylococcus aureus Growth Independent of LtaS Function
Source: ACS Infect Dis. 2023 Oct 13;9(11):2141–59. doi: 10.1021/acsinfecdis.3c00250 (PMC10644342; doi:10.1021/acsinfecdis.3c00250)
Supplement: Supplementary file 1 — id3c00250_si_001.pdf [file id3c00250_si_001.pdf]

Supporting Information

Improved antibacterial activity of 1,3,4 oxadiazole-based compounds that restrict *Staphylococcus aureus* growth independent of LtaS function

Edward J.A. Douglas[1], Brandon Marshall[2], Arwa Alghamadi[2], Erin A. Joseph,[2] Seána Duggan[3], Serena di Vittorio[4], Laura De Luca[4], Michaela Serpi[2] and Maisem Laabei[1*]*

[1] Department of Life Sciences, University of Bath, Bath BA2 7AY, UK

[2] School of Chemistry, Cardiff University, Cardiff CF10 3AT, Wales, UK

[3] Medical Research Council Centre for Medical Mycology at the University of Exeter, University of Exeter, Exeter EX4 4DQ, UK

[4] Department of Chemical, Biological, Pharmaceutical and Environmental Sciences, University of Messina, Messina I-98125, Italy

* For correspondence, please email ml418@bath.ac.uk or serpim5@cardiff.ac.uk

Keywords: *Staphylococcus aureus*; lipoteichoic acid inhibitors; antimicrobial resistance; drug discovery

Supplementary Table 1: List of strains used in this study.

Strain	Description	Reference
S. aureus		
LAC	CA-MRSA; CC 8 (USA300); Type IV SCC*mec*; North American pandemic clone	[1]
LAC pRMC2	LAC transformed with empty pRMC2	This study
LAC p*ltaS*	LAC transformed with pRMC2 complemented with *ltaS*	This study
Newman (ANG 112)	MSSA; CC8; Extensively used laboratory strain, isolated from human infection	[2]
Newman Δ*tarO* (ANG 260)	*tarO* knockout mutant in the Newman background	[3]
Newman Δ*tarO* pRMC2	Newman Δ*tarO* transformed with empty pRMC2	This study
Newman Δ*tarO* p*ltaS*	Newman Δ*tarO* transformed with pRMC2 complemented with *ltaS*	This study
MW2	CA-MRSA; CC1 (USA100); Type IV SCC*mec*; dominant in America	[4]
SH1000	MSSA; CC8; Extensively used laboratory strain, *rsbU*$^+$ version of 8325-4	[5]
MRSA252	HA-MRSA; CC30 (EMRSA-16 clone/USA200); Type II SCC*mec*; historically globally disseminated, high prevalence in the UK	[6]
TW20	HA-MRSA; CC239; Type III SCC*mec*; pandemic MRSA strain	[7]
Mu50	VISA; CC5; Type II SCC*mec*; first clinical vancomycin resistant/intermediate *S aureus* strain	[8]
Mu3	hVISA: CC5; Type II SCC*mec;* hetero-VISA clinical isolate	[8]
EMRSA-15	HA/CA-MRSA; CC22 Type IV SCC*mec*; globally disseminated, high prevalence in Europe	[9]
8325-4	8325-4 wild type strain (KB1079)	[10]

8325-4 *clpX*	*clpX* knockout mutant in 8325-4 (HI2300)	11
8325-4 *clpX-ltaSTOP*	*clpX* knockout and *ltaS*382STOP mutant in 8325-4 (KB1133)	12
SA564	SA564 (KB1048)	13
SA564 *clpX*	*clpX* knockout mutant in SA564 (KB1049)	14
SA564 *clpX-ltaSTOP*	*clpX* knockout and *ltaS*H476Q mutant in SA564	12
JE2	JE2	15
JE2 *clpX*I265E	*clpX*I265E mutant in JE2	15
JE2 *ltaSTOP*	*clpX*I265E and *ltaS*339STOP mutant in JE2	D. Frees (Unpublished)
8325-4	8325-4	16
M0674N	8325-4 Δ*ltaS*::phleo^R	16, 17
<u>*Staphylococcus epidermidis*</u>		
RP62A	*S. epidermidis* reference strain	18
311	Clinical isolate 311	19
771	Clinical isolate 771	19
780	Clinical isolate 780	19
319	Clinical isolate 319	19
322	Clinical isolate 322	19
305	Clinical isolate 305	19
<u>*Other Gram-positive organisms*</u>		
W168	*Bacillus subtilis* laboratory strain	20
JH2-2	*Enterococcus faecalis* human stool isolate	21
C68	*Enterococcus faecium* Clade A1 isolate	22
NCTC 8198	*Streptococcus pyogenes* reference strain	NCTC
NCTC 12048	*S. pyogenes* reference strain	NCTC
NCTC8181	*Streptococcus agalactiae* reference strain	NCTC
COH1	*S. agalactiae* reference strain	23

NCTC 13762	*Streptococcus dysgalactiae* reference strain	NCTC
NCTC 10238	*S. dysgalactiae* reference strain	NCTC
<u>Gram-negative organisms</u>		
K12	*Escherichia coli* reference strain	[24]
PAO1	*Pseudomonas aeruginosa* reference strain	[25]
699	*Klebsiella pneumoniae* clinical isolate	[26]
DF1000	*Acinetobacter baumannii* clinical isolate	[27]

Healthcare-acquired MRSA, HA-MRSA; community acquired MRSA, CA-MRSA;

Supplementary Table 2: Cell health index of diverse compounds including clinically relevant antibiotics

AsedaSciences Library Compound	Cell Morphology	Cytoplasmic Membrane Integrity	ROS (mt superoxide)	Glutathione	Nuclear Membrane Integrity 1	Cell cycle	Nuclear membrane Integrity 2	Mitochondrial Membrane Polarization	Cell Health Index
Sucralose	0.00	0.00	0.00	0.01	0.16	0.02	0.07	0.00	0.10
Acebutolol	0.00	0.01	0.07	0.02	0.19	0.14	0.04	0.00	0.10
Metformin	0.00	0.04	0.13	0.04	0.03	0.11	0.08	0.01	0.11
Abacavir	0.00	0.04	0.02	0.02	0.03	0.03	0.00	0.00	0.11
Isopropamide	0.03	0.01	0.02	0.01	0.05	0.09	0.00	0.00	0.12
Ribavirin	0.07	0.02	0.04	0.08	0.09	0.01	0.09	0.01	0.14
Atenolol	0.01	0.01	0.01	0.03	0.07	0.10	0.00	0.00	0.14
Levamisole	0.01	0.01	0.02	0.06	0.01	0.07	0.02	0.01	0.15
Dexamethasone	0.02	0.03	0.18	0.06	0.08	0.02	0.13	0.01	0.15
Rosuvastatin	0.00	0.00	0.02	0.02	0.11	0.39	0.01	0.00	0.15
Isoxicam	0.01	0.15	0.04	0.05	0.22	0.07	0.04	0.01	0.15
Iproniazid	0.00	0.01	0.04	0.03	0.16	0.40	0.10	0.00	0.15
Vancomycin	0.01	0.01	0.06	0.01	0.03	0.29	0.29	0.02	0.16
Ranitidine	0.00	0.03	0.02	0.03	0.09	0.03	0.22	0.00	0.16
Penicillamine	0.02	0.02	0.09	0.04	0.04	0.12	0.01	0.01	0.17
Indomethacin	0.00	0.02	0.18	0.01	0.04	0.09	0.00	0.02	0.17
Lidocaine	0.01	0.00	0.26	0.01	0.11	0.26	0.25	0.01	0.17
Aspartame	0.02	0.03	0.08	0.01	0.07	0.06	0.06	0.02	0.17
Chloroquine	0.01	0.23	0.02	0.03	0.07	0.08	0.10	0.00	0.17
Hydrochlorothiazide	0.02	0.37	0.05	0.02	0.05	0.09	0.33	0.01	0.17
Caffeine	0.01	0.03	0.18	0.05	0.12	0.17	0.07	0.04	0.18
Fexofenadine	0.01	0.00	0.06	0.01	0.05	0.02	0.03	0.04	0.18
Acetaminophen	0.00	0.01	0.42	0.02	0.30	0.03	0.02	0.02	0.18
Atorvastatin	0.00	0.05	0.03	0.04	0.03	0.06	0.09	0.17	0.18
Niacin	0.00	0.04	0.01	0.02	0.09	0.12	0.05	0.12	0.19
Streptomycin	0.02	0.05	0.08	0.01	0.02	0.16	0.09	0.02	0.19
Nicotine	0.00	0.04	0.04	0.05	0.32	0.08	0.01	0.02	0.20
Lovastatin	0.00	0.00	0.02	0.01	0.06	0.04	0.18	0.15	0.20
Indoprofen	0.05	0.07	0.03	0.11	0.11	0.24	0.05	0.04	0.20
Aspirin	0.01	0.21	0.08	0.03	0.11	0.01	0.11	0.02	0.20
Famotidine	0.05	0.05	0.03	0.02	0.13	0.06	0.05	0.09	0.21
Ibuprofen lysine	0.28	0.08	0.30	0.03	0.16	0.18	0.35	0.02	0.21
Ascorbic acid	0.00	0.03	0.09	0.15	0.23	0.02	0.07	0.02	0.21
Fluvastatin	0.01	0.04	0.07	0.07	0.46	0.07	0.40	0.10	0.21
Diphenhydramine	0.03	0.21	0.11	0.02	0.02	0.05	0.09	0.01	0.22
Erythromycin	0.00	0.02	0.01	0.09	0.07	0.08	0.07	0.02	0.22
Melatonin	0.00	0.12	0.13	0.01	0.22	0.06	0.04	0.02	0.22
Salicylic acid	0.05	0.32	0.13	0.10	0.06	0.14	0.00	0.01	0.22
Buspirone	0.01	0.04	0.01	0.04	0.04	0.34	0.05	0.01	0.23
Disopyramide	0.04	0.12	0.15	0.30	0.23	0.06	0.04	0.02	0.23
Saccharin	0.00	0.21	0.07	0.02	0.25	0.27	0.05	0.01	0.23
Zolpidem	0.01	0.03	0.05	0.34	0.05	0.46	0.02	0.02	0.24
Methotrexate	0.02	0.10	0.38	0.11	0.30	0.13	0.21	0.10	0.24
Pioglitazone	0.50	0.57	0.03	0.66	0.39	0.32	0.08	0.06	0.30
Folate	0.07	0.21	0.04	0.07	0.19	0.27	0.01	0.11	0.31
Trazodone	0.00	0.44	0.02	0.01	0.37	0.13	0.05	0.01	0.32
Alpidem	0.94	0.18	0.96	0.69	0.78	0.69	0.12	0.16	0.39
Nelfinavir	0.74	0.61	0.87	0.21	0.06	0.82	0.73	0.85	0.47
Cisapride	0.95	0.96	0.06	0.20	0.19	0.48	0.67	0.55	0.52
Valinomycin	0.98	0.95	0.90	0.83	0.34	0.50	0.54	0.85	0.55
Tolcapone	1.00	0.76	1.00	0.71	0.36	0.96	0.40	0.36	0.56
TAK-715	0.74	0.76	0.97	0.19	0.97	0.96	0.74	0.51	0.57
Celecoxib	0.58	0.95	0.95	0.29	0.43	0.74	0.54	0.58	0.61
Rotenone	0.99	0.81	0.99	0.90	0.70	0.55	0.66	0.99	0.67
Amiodarone	0.99	0.47	0.40	0.28	0.73	0.92	0.44	0.87	0.68
ABT-737	1.00	0.98	0.98	0.99	0.87	0.89	0.55	0.97	0.70
Niclosamide	0.99	0.97	0.99	0.73	1.00	0.81	0.93	0.98	0.75
Nefazodone	0.99	1.00	1.00	1.00	0.90	0.96	0.96	1.00	0.77
Chlorpromazine	1.00	1.00	1.00	1.00	1.00	0.82	1.00	0.99	0.84
Doxorubicin	0.98	0.82	1.00	0.92	0.95	1.00	0.97	0.99	0.85
Fluphenazine	1.00	1.00	1.00	1.00	1.00	0.96	1.00	0.98	0.86
Daunorubicin	1.00	0.26	1.00	0.97	0.42	1.00	1.00	1.00	0.87
RWJ-67657	1.00	1.00	0.99	0.99	0.74	1.00	0.92	1.00	0.88
Saquinavir	1.00	1.00	1.00	1.00	0.99	0.97	0.93	1.00	0.88
Lapatinib	1.00	1.00	0.92	0.89	0.96	0.95	0.98	1.00	0.88
Mefloquine	1.00	1.00	1.00	1.00	1.00	0.99	1.00	1.00	0.93
AMG-548	1.00	1.00	1.00	1.00	1.00	0.96	1.00	1.00	0.93
Tamoxifen	1.00	1.00	1.00	1.00	1.00	0.99	1.00	1.00	0.94

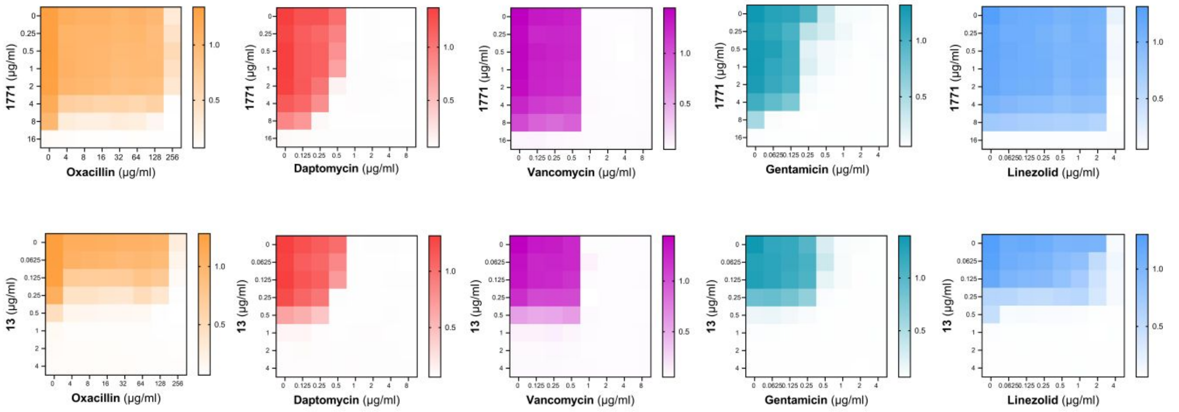

	FIC indexes	
**Antibiotic combination**	1771	Compound 13
Oxacillin	0.75	0.75
Daptomycin	0.625	0.75
Vancomycin	1	1
Gentamicin	0.375	0.75
Linezolid	1	0.515

Figure S1. Checkerboard analysis of 1771 and compound 13 in combination with clinically relevant antibiotics. *Checkerboard analysis was repeated twice for each antibiotic combination, and the mean bacterial growth (OD_{600nm}) plotted as a heat map with 1771/compound* ***13*** *concentration of the y-axis and test antibiotic concentration on the x axis. FIC indexes were calculated for each combination and the lowest possible value presented. The interpretation of the FIC index was designated synergistic (≤0.5), additive (>0.5, <1) or indifferent (≥1, <4).*

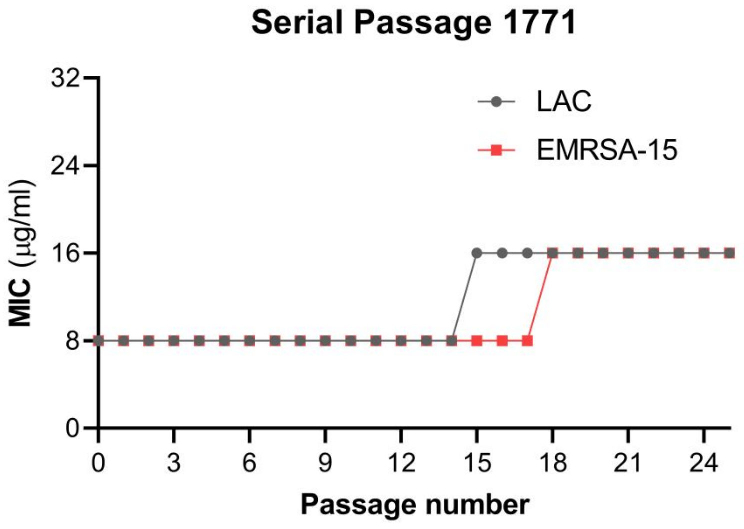

Figure S2: Resistance profile of 1771. *Serial passage of 1771 against MRSA strains LAC and EMRSA-15. MIC was evaluated for a total of 25 days serial passage in 1771.*

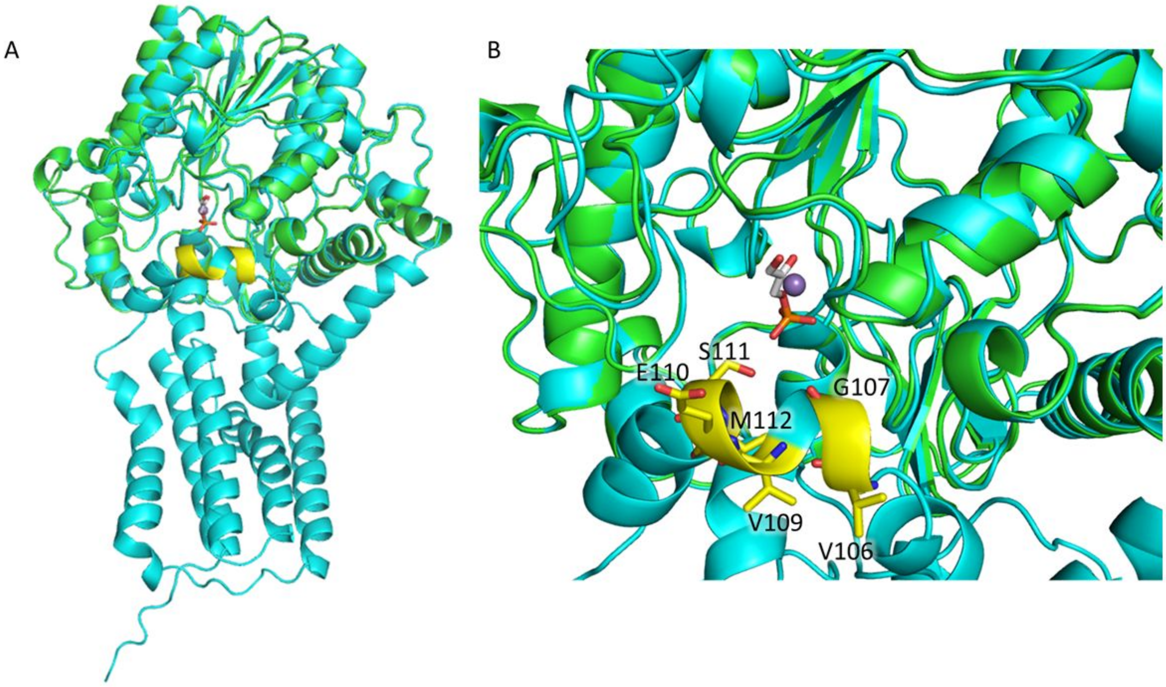

Figure S3: Molecular modelling of LtaS. A) *AlphaFold LtaS model (retrieved from AlphaFold repository AF-Q7A1I3-F1) aligned to the x-ray structure of LtaS extracellular domain in complex with glycerol phosphate (PDB ID 2W5S).* ***B)*** *Close view of LtaS active site. AlphaFold model and the x-ray extracellular domain of LtaS are depicted as cyan and green cartoon, respectively. The residues of AlphaFold structure located close to the active site and predicted with a low confidence score are represented in yellow. The substrate glycerol phosphate is displayed as grey sticks.*

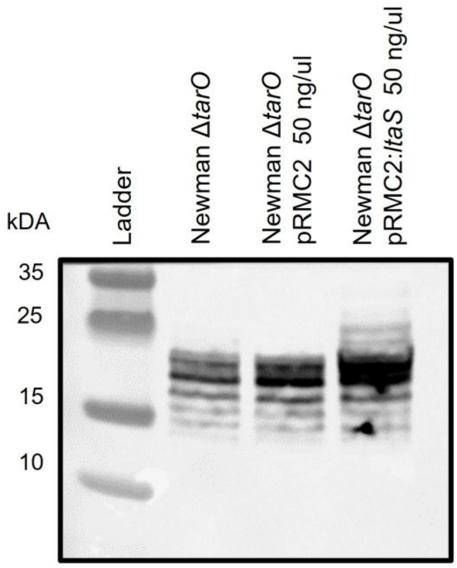

Figure S4. LTA expression following induction of *LtaS.* *LTA western blot was performed on strain Newman ΔtarO, Newman ΔtarO containing the empty pRMC2 vector or Newman ΔtarO housing the pRMC2:ltaS vector. Strains harbouring the pRMC2 vector were grown in the presence of the chemical inducer anhydrotetracycline (50 ng/ml).*

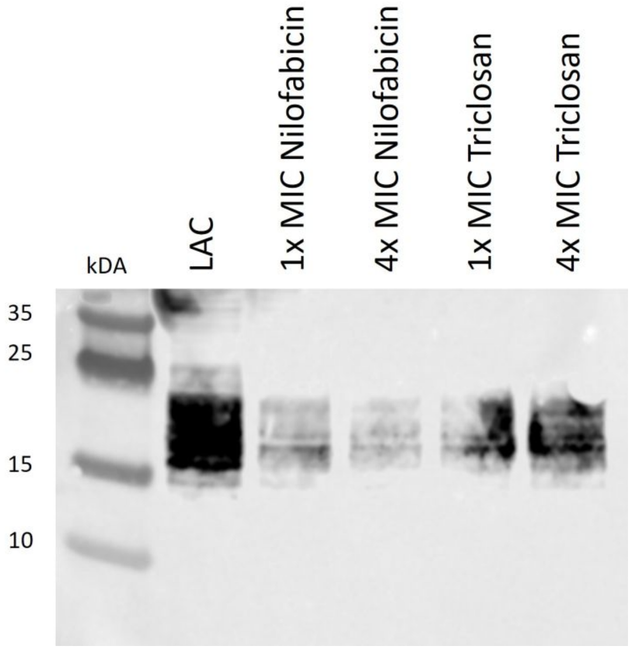

Figure S5. LTA production is inhibited following treatment with fatty acid inhibitors. *LTA western blot was performed on strain LAC in the presence of either 1X or 4X the MIC of nilofabicin or triclosan as described in Materials and Methods.*

A)

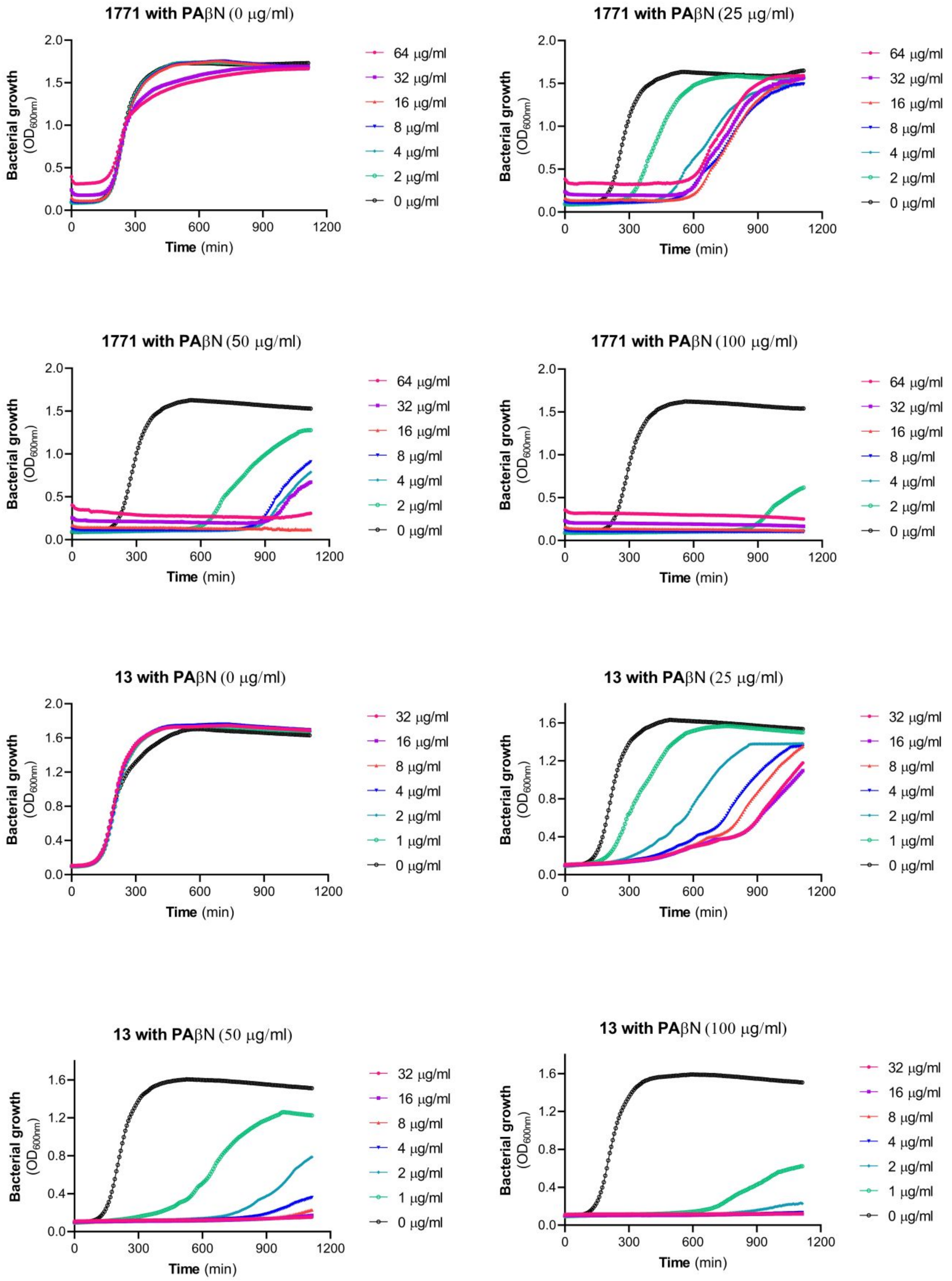
1771 with PAβN (0 μg/ml)
1771 with PAβN (25 μg/ml)
1771 with PAβN (50 μg/ml)
1771 with PAβN (100 μg/ml)
13 with PAβN (0 μg/ml)
13 with PAβN (25 μg/ml)
13 with PAβN (50 μg/ml)
13 with PAβN (100 μg/ml)
Bacterial growth (OD600nm)
Time (min)
64 μg/ml
32 μg/ml
16 μg/ml
8 μg/ml
4 μg/ml
2 μg/ml
1 μg/ml
0 μg/ml

B)

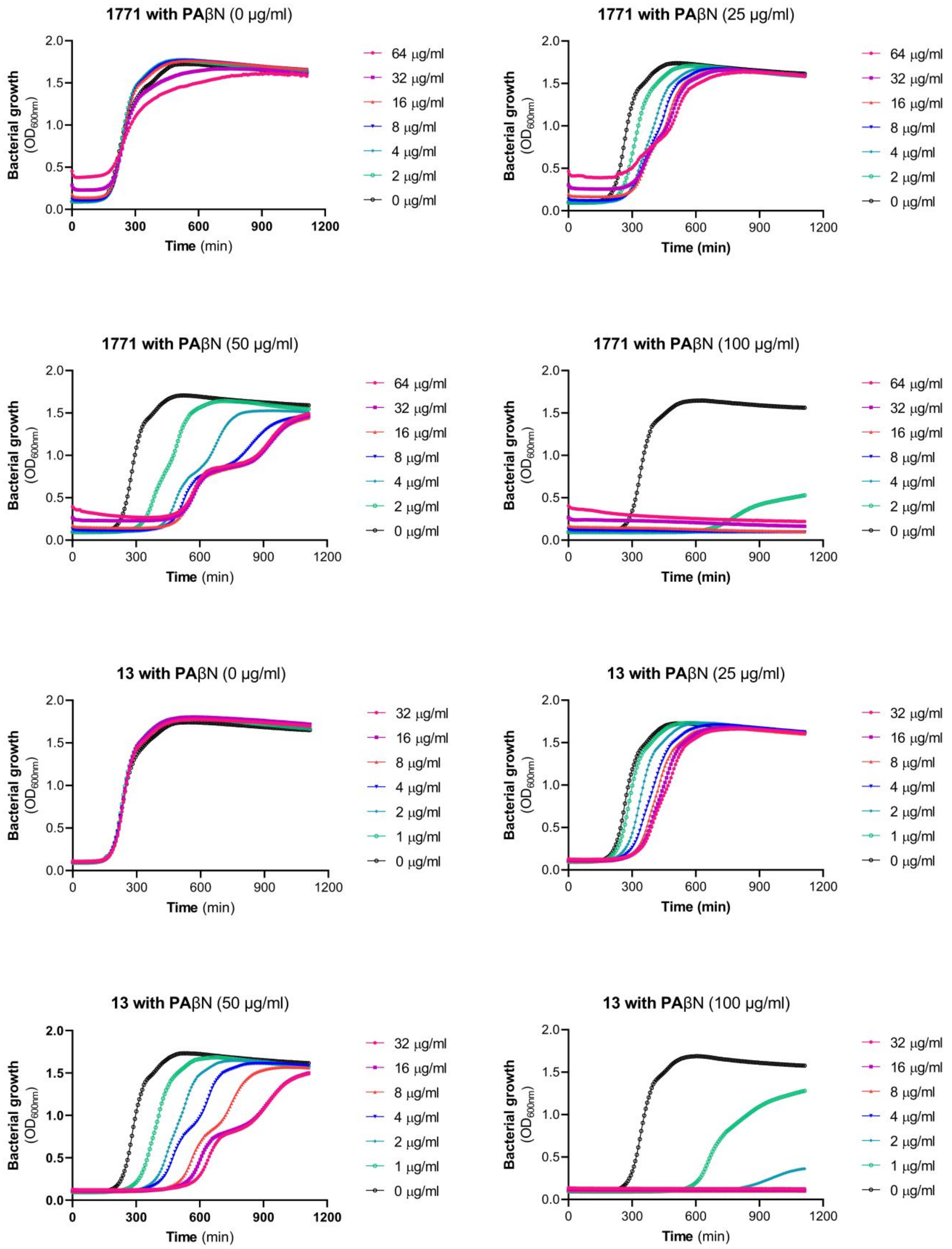
1771 with PAβN (0 µg/ml)
1771 with PAβN (25 µg/ml)
1771 with PAβN (50 µg/ml)
1771 with PAβN (100 µg/ml)
64 µg/ml
32 µg/ml
16 µg/ml
8 µg/ml
4 µg/ml
2 µg/ml
0 µg/ml
13 with PAβN (0 µg/ml)
13 with PAβN (25 µg/ml)
13 with PAβN (50 µg/ml)
13 with PAβN (100 µg/ml)
32 µg/ml
16 µg/ml
8 µg/ml
4 µg/ml
2 µg/ml
1 µg/ml
0 µg/ml
Bacterial growth (OD_{600nm})
Time (min)
0.0
0.5
1.0
1.5
2.0
0
300
600
900
1200

C)

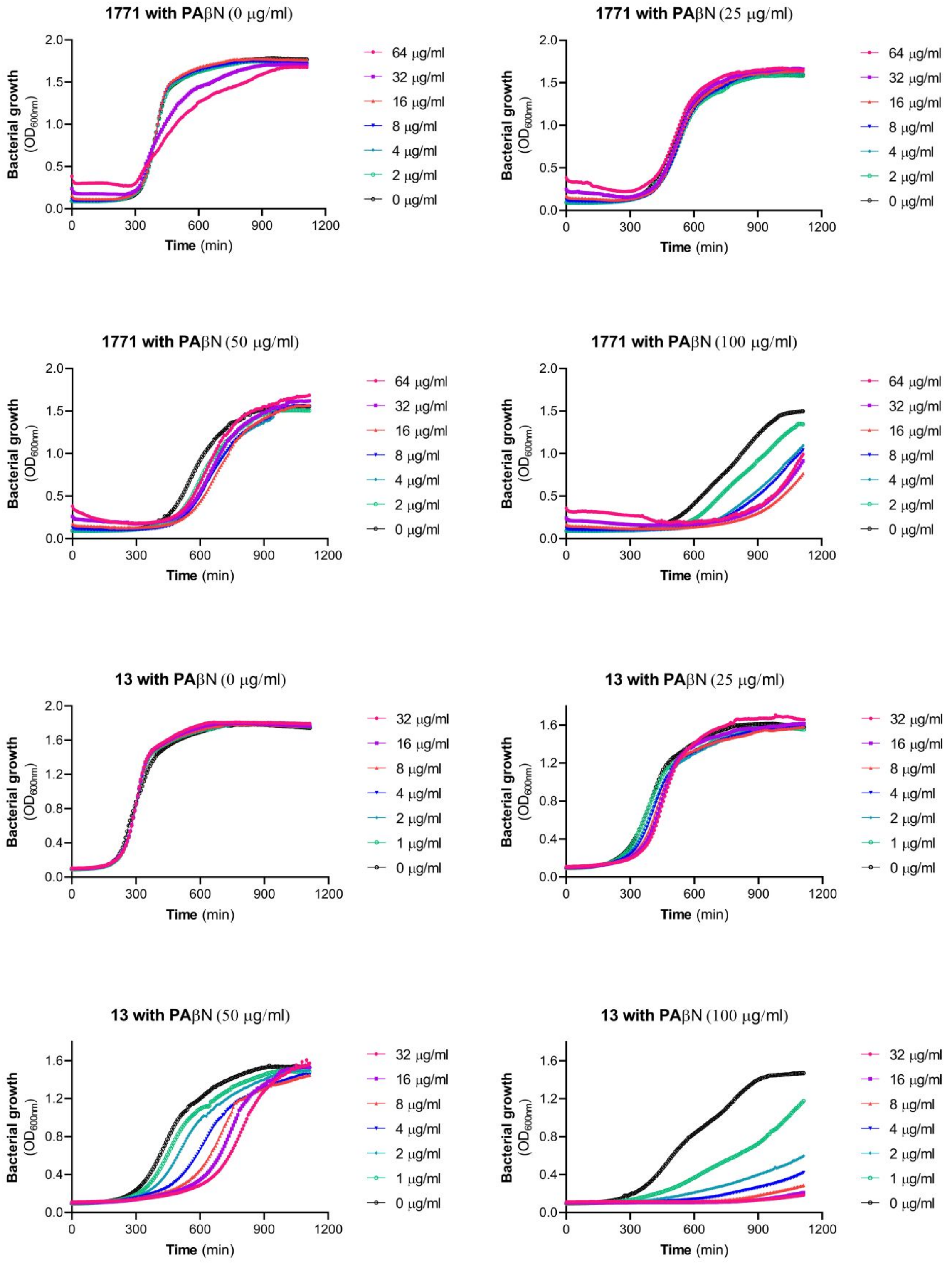

Figure S6. ***Permeabilization of the outer membrane renders Gram-negative bacteria susceptible to 1771 and compound 13.*** *Bacterial growth curves of A) Klebsiella pneumoniae, B) Acinetobacter baumannii and C) Pseudomonas aeruginosa against 1771 and* ***13*** *(0 – 64 µg/ml) in combination with 0, 25, 50, 100 µg/ml of PAβN. The bacterial growth (OD_{600nm}) was*

plotted against time following 18 hours of growth at 37°C. The icons represent the mean of three biological replicates.

Supplementary Materials and Methods

Synergistic activity with clinically relevant antibiotics

The synergistic ability of 1771 and **13** was achieved using the chequerboard micro-broth dilution method. Antibiotic partners tested included: oxacillin, daptomycin, vancomycin, gentamicin, and linezolid with starting concentrations of 256 µg/ml, 8 µg/ml, 8 µg/ml, 4 µg/ml, and 8 µg/ml respectively. Overnight cultures were sub-cultured 1:200 in fresh MHB and grown to optical density of OD600nm of 0.5-0.6 and prepared according to the MIC analysis. Following an 18 h incubation at 37ºC the sum of the fractional inhibitory concentration (∑FIC) was calculated, for each well for a combination that resulted in an MIC, using the equation ΣFIC = FICA +FICB = (MICA in combination with B/MICA) + (MICB in combination with A/MICB). The resulting value was interpreted according to the following rules: synergistic (FIC ≤ 0.5), additive (FIC >0.5, <1) or indifferent (FIC ≥ 1 <4).

Serial passage resistance study

A serial passage in 1771 was performed using MRSA strains, LAC and EMRSA-15. For this assay, 15 ml tubes were filled with 3 ml of MHB and varying concentrations of 1771. The tubes containing either LAC or EMRSA-15 were incubated at 37°C for 24 h before each passage. The passage was performed by transferring 30 µl aliquot of overnight growth from the tube nearest the MIC with the same turbidity as the negative control into a new 3 ml solution containing increased concentrations of 1771. This procedure was followed for a total of 25 days (25 serial passages) in an effort to induce spontaneous resistance against 1771.

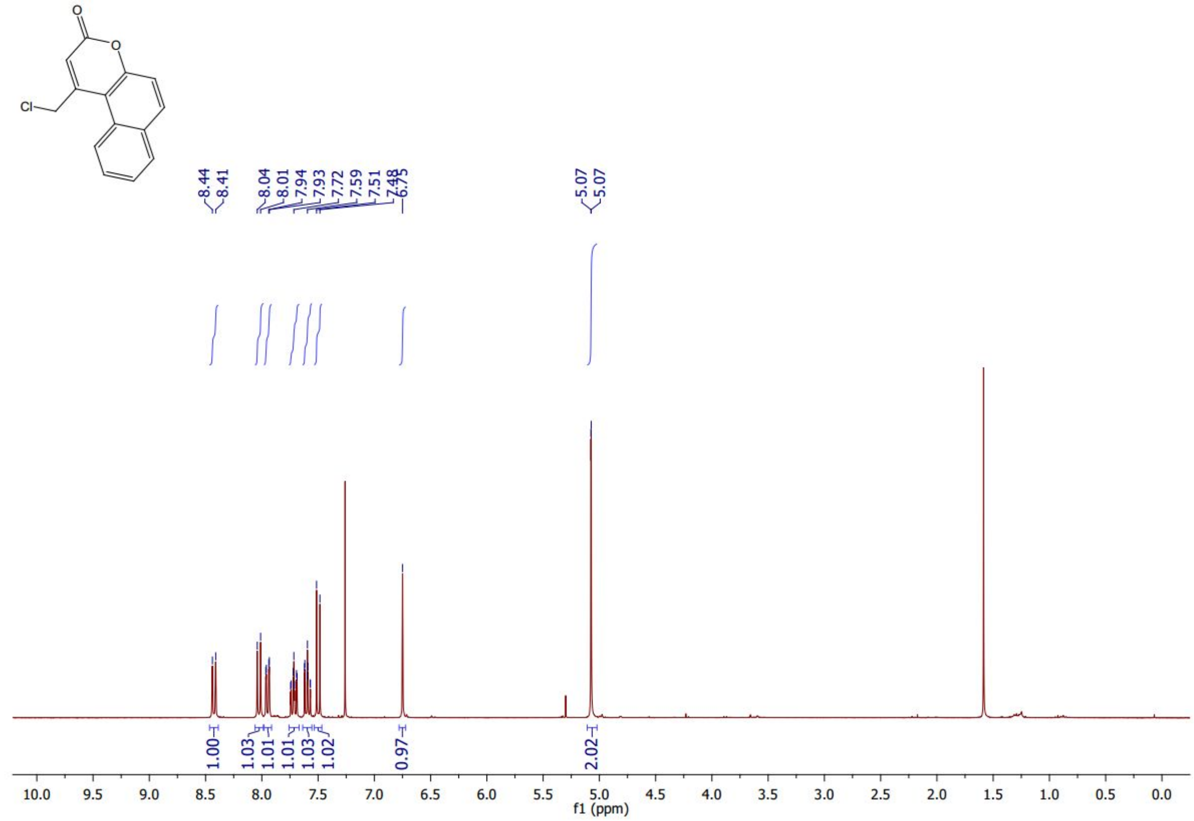

Figure S7: ^{1}H-NMR (300 MHz, $CDCl_3$) of compound **4a**

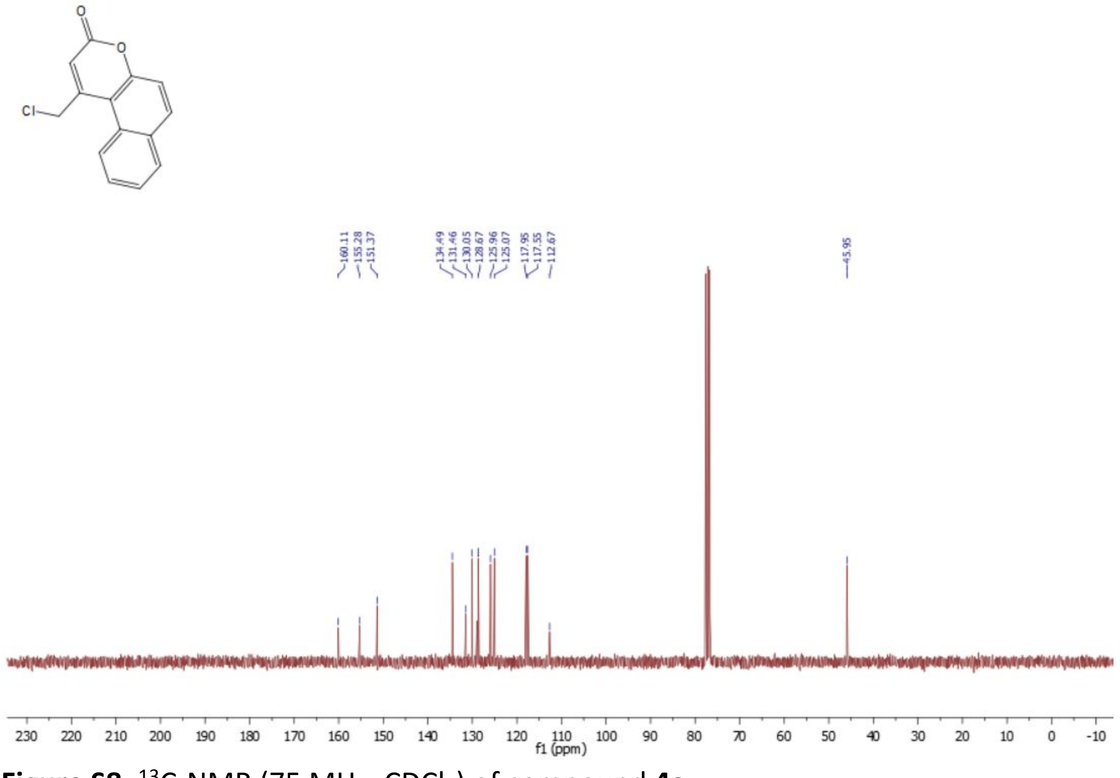

Figure S8: ^{13}C-NMR (75 MHz, $CDCl_3$) of compound **4a**

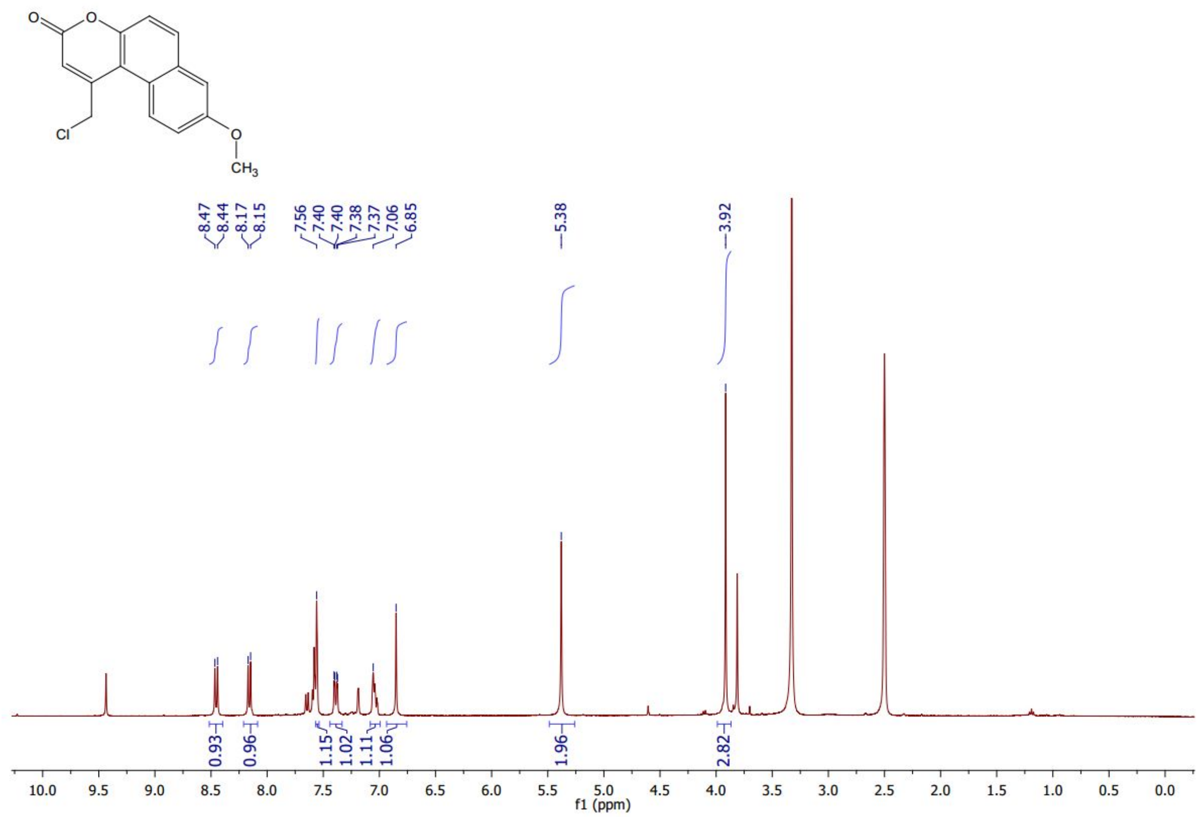

Figure S9: ^{1}H-NMR (400 MHz, DMSO-d_6) of compound **4b**

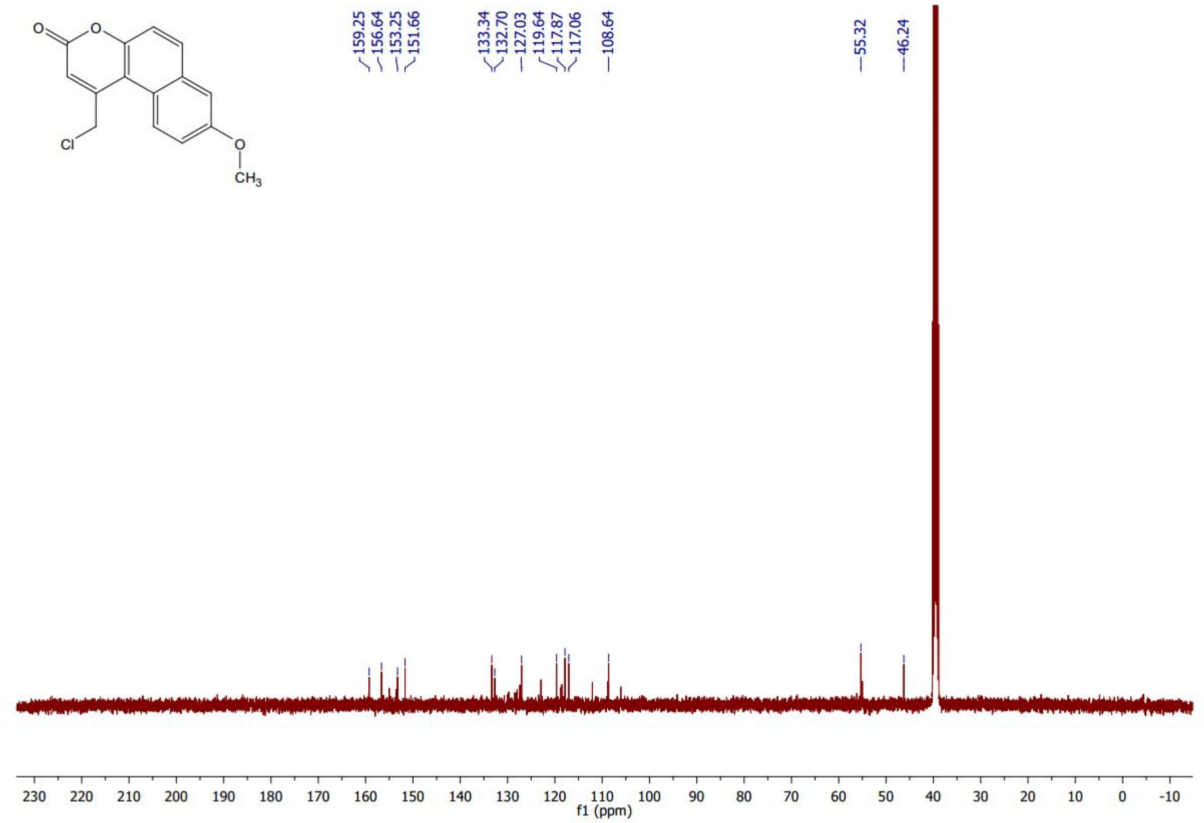

Figure S10: ^{13}C-NMR (100 MHz, DMSO-d_6) of compound **4b**

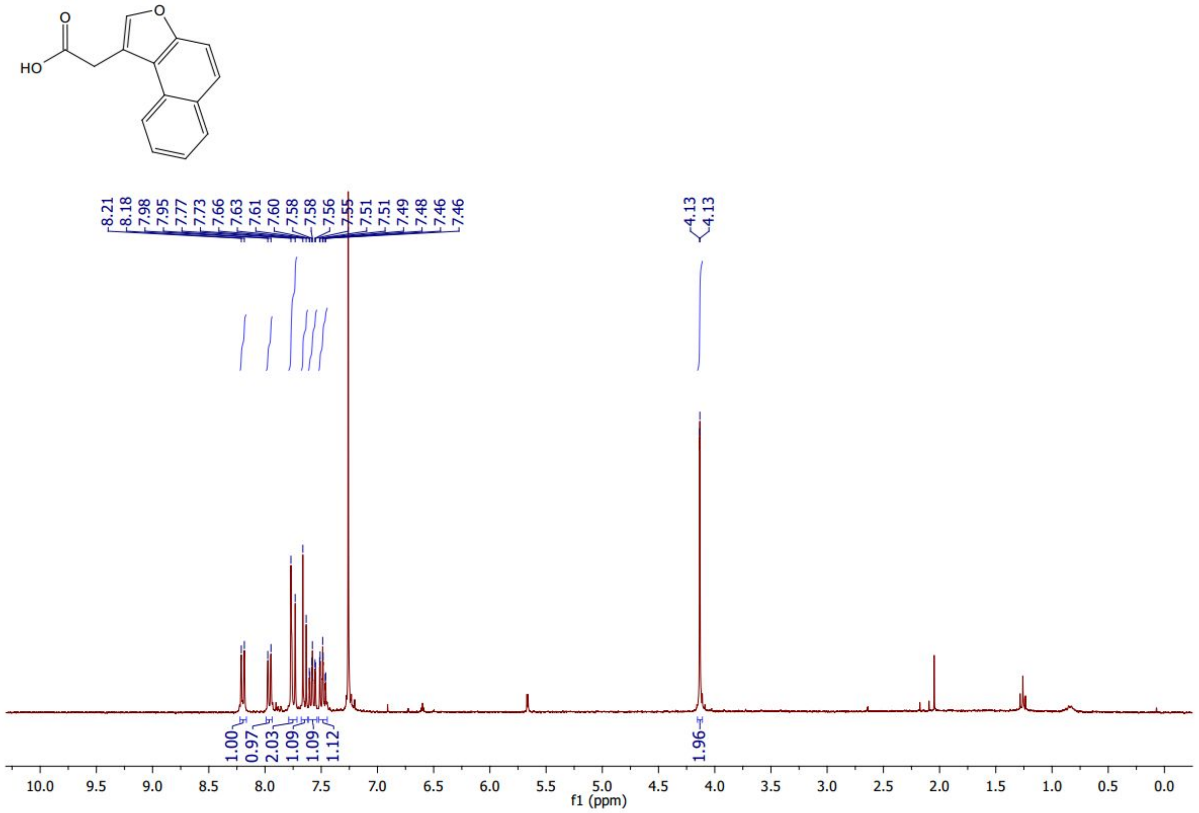

Figure S11: ^{1}H-NMR (300 MHz, $CDCl_3$) of compound **5a**

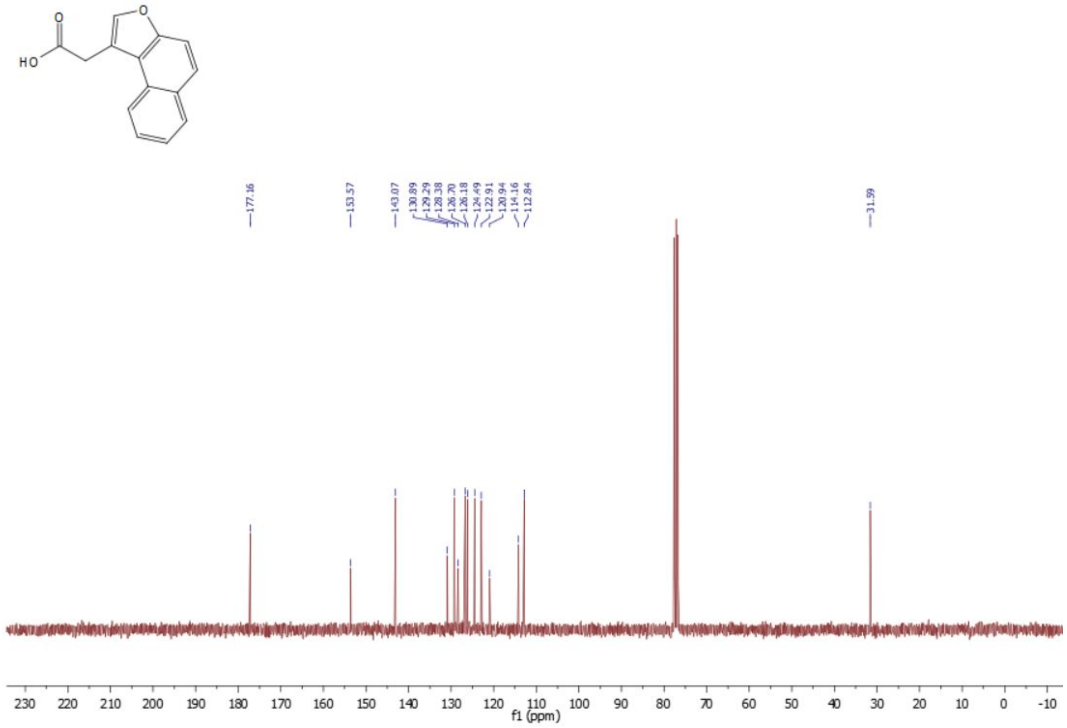

Figure S12: ^{13}C-NMR (75 MHz, $CDCl_3$) of compound **5a**

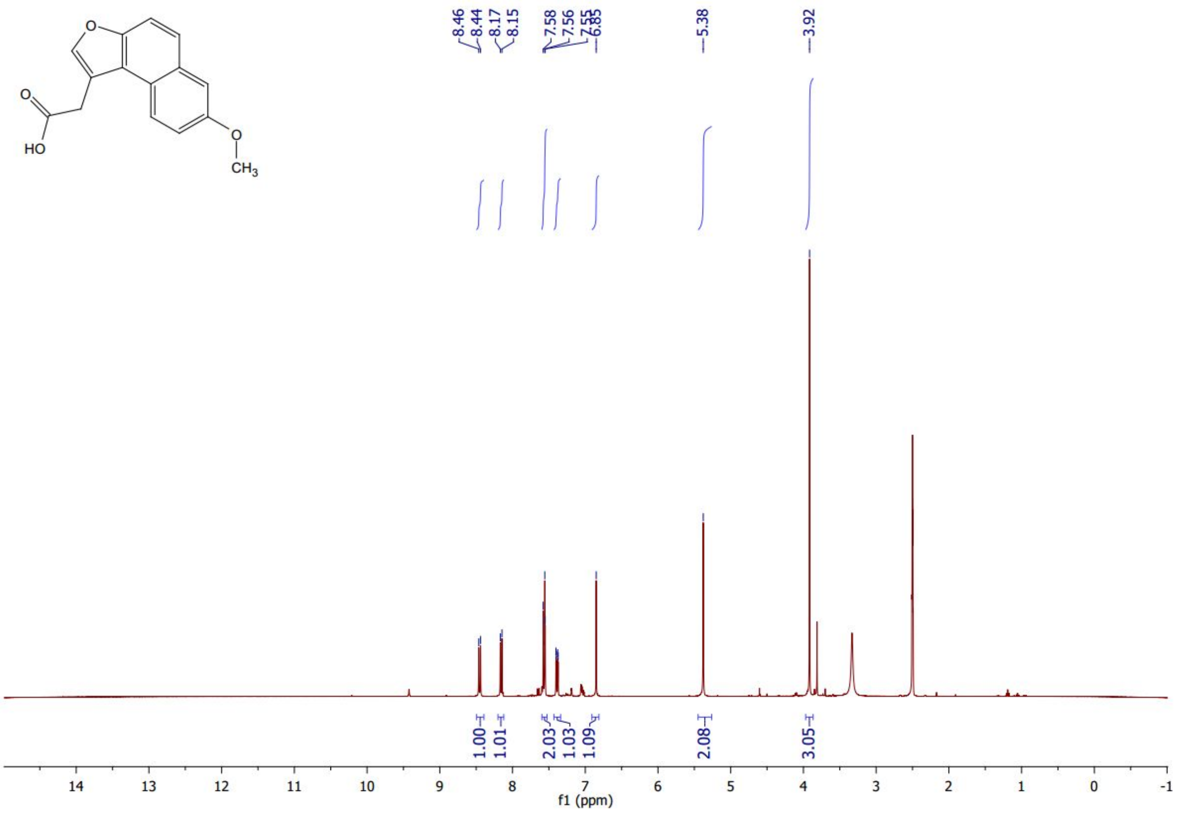

Figure S13: ^{1}H-NMR (300 MHz, DMSO-d$_6$) of compound **5b**

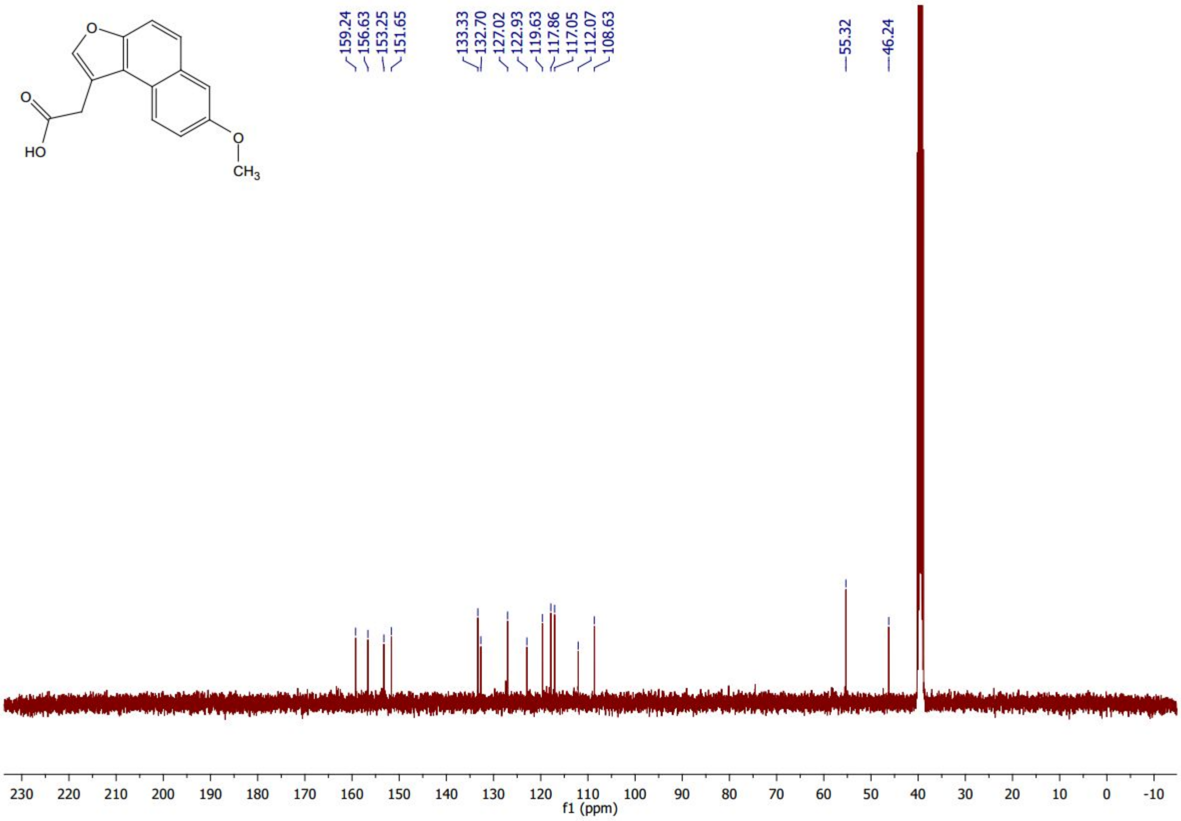

Figure S14: ^{13}C-NMR (75 MHz, DMSO-d$_6$) of compound **5b**

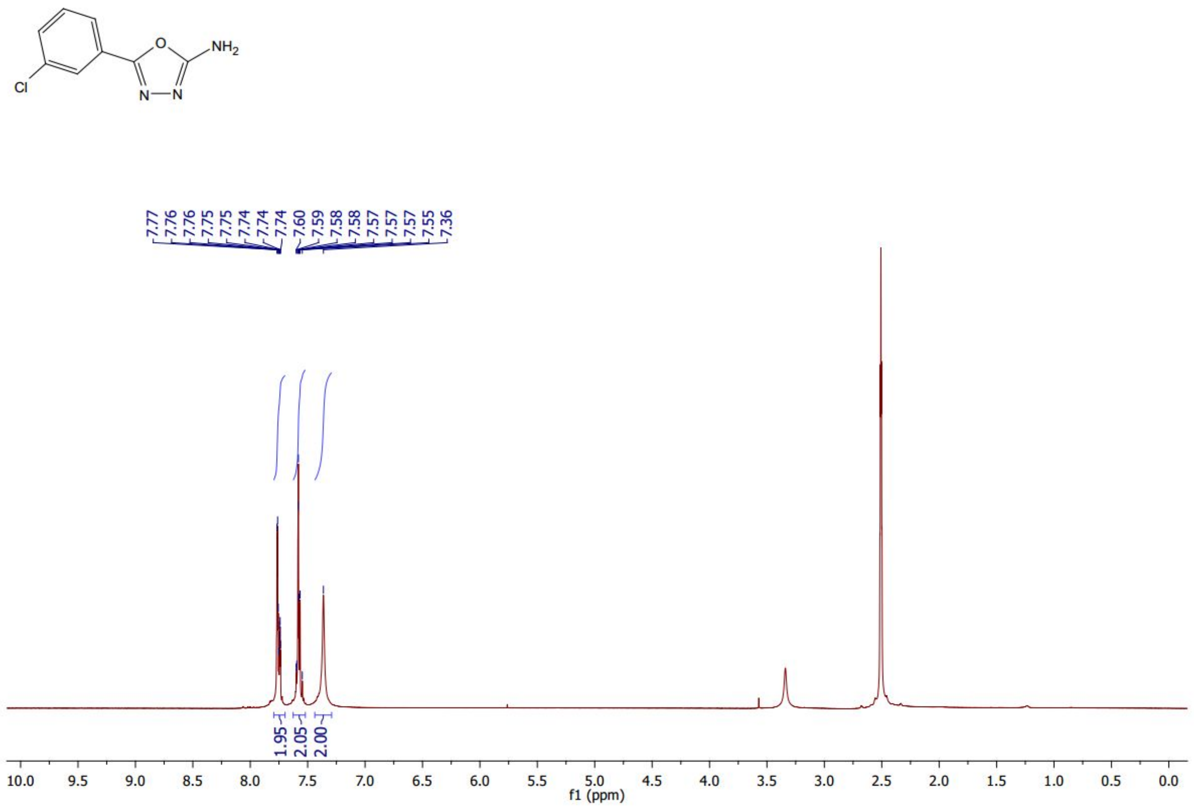

Figure S15: ^{1}H-NMR (400 MHz, DMSO-d$_6$) of compound **7b**

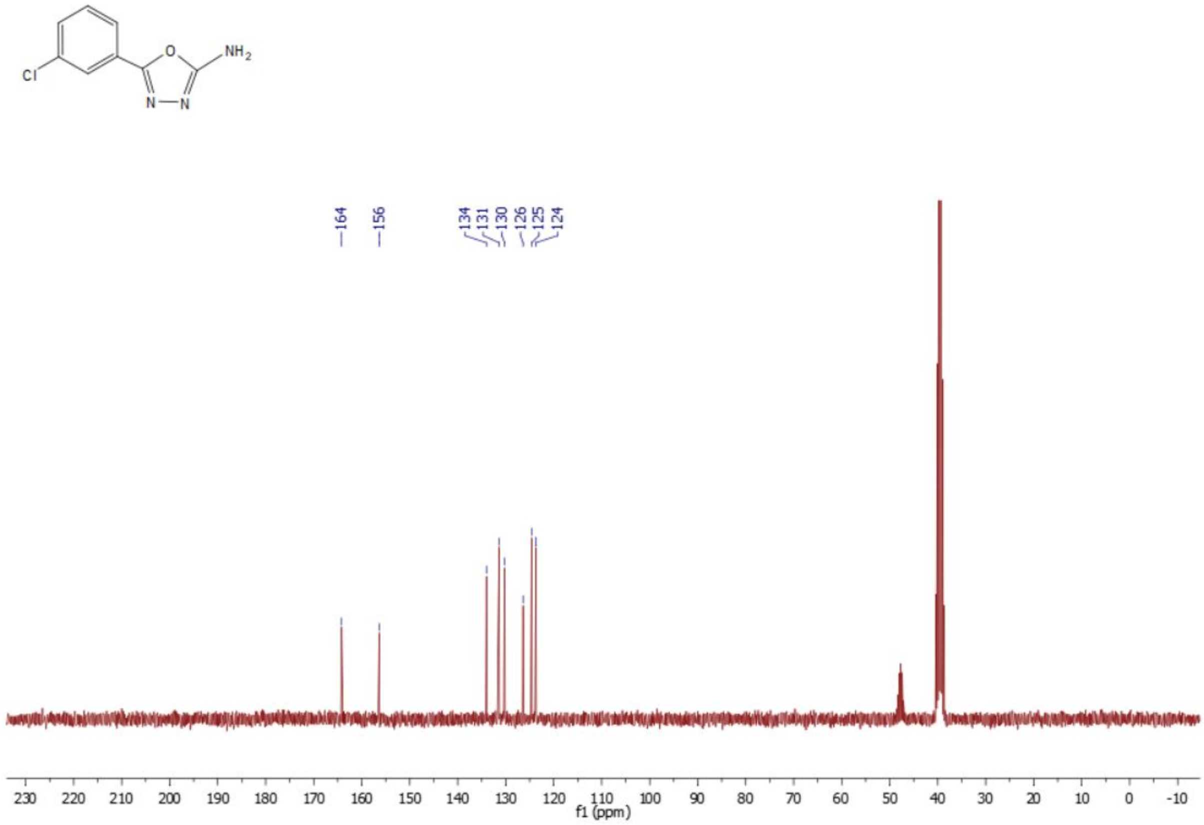

Figure S16: ^{13}C-NMR (75 MHz, DMSO-d$_6$) of compound **7b**

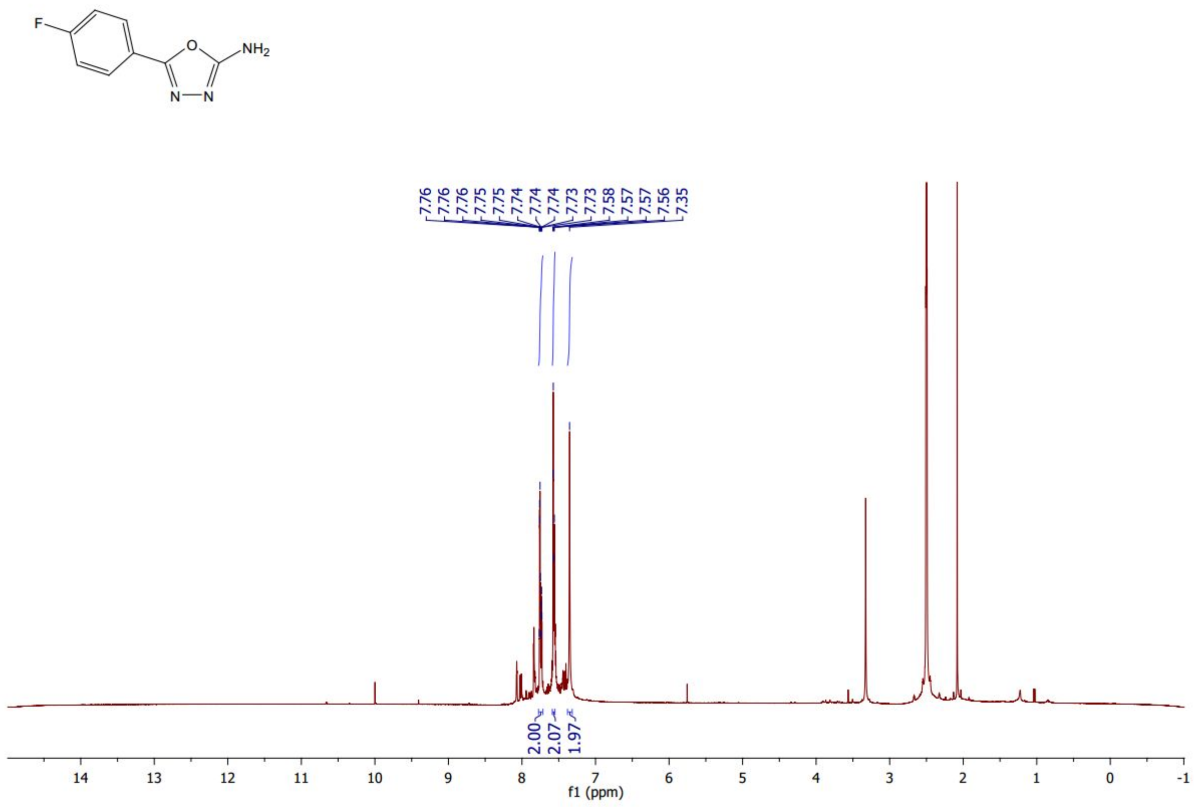

Figure S17: ^{1}H-NMR (300 MHz, DMSO-d$_6$) of compound **7c**

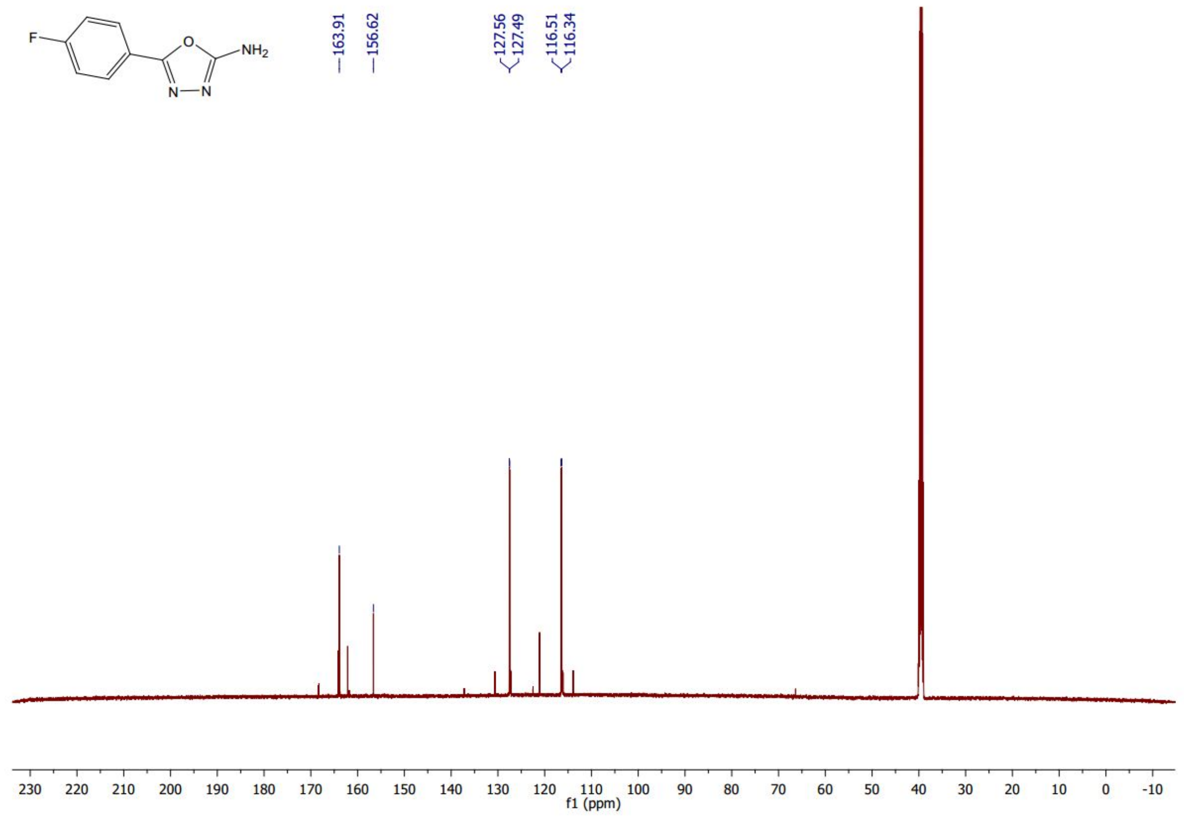

Figure S18: ^{13}C-NMR (125 MHz, DMSO-d$_6$) of compound **7c**

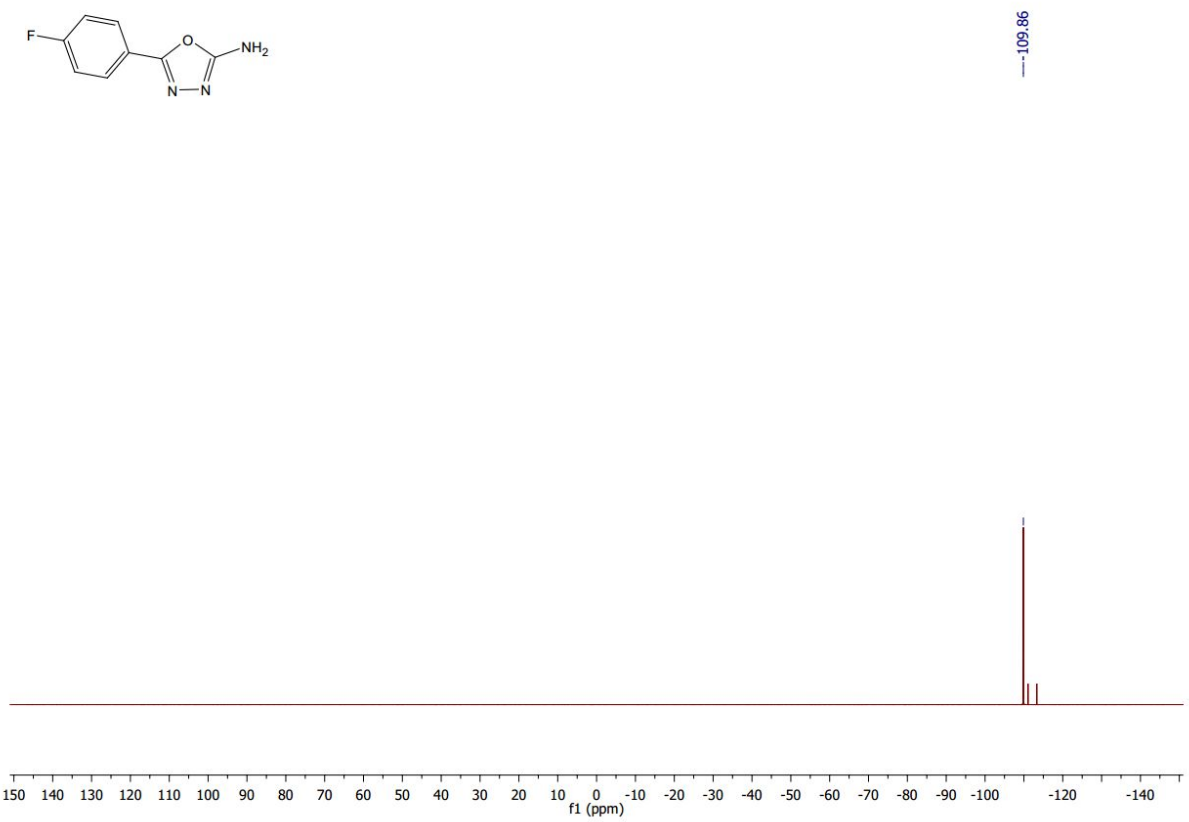

Figure S19: ^{19}F-NMR (470 MHz, DMSO-d_6) of compound **7c**

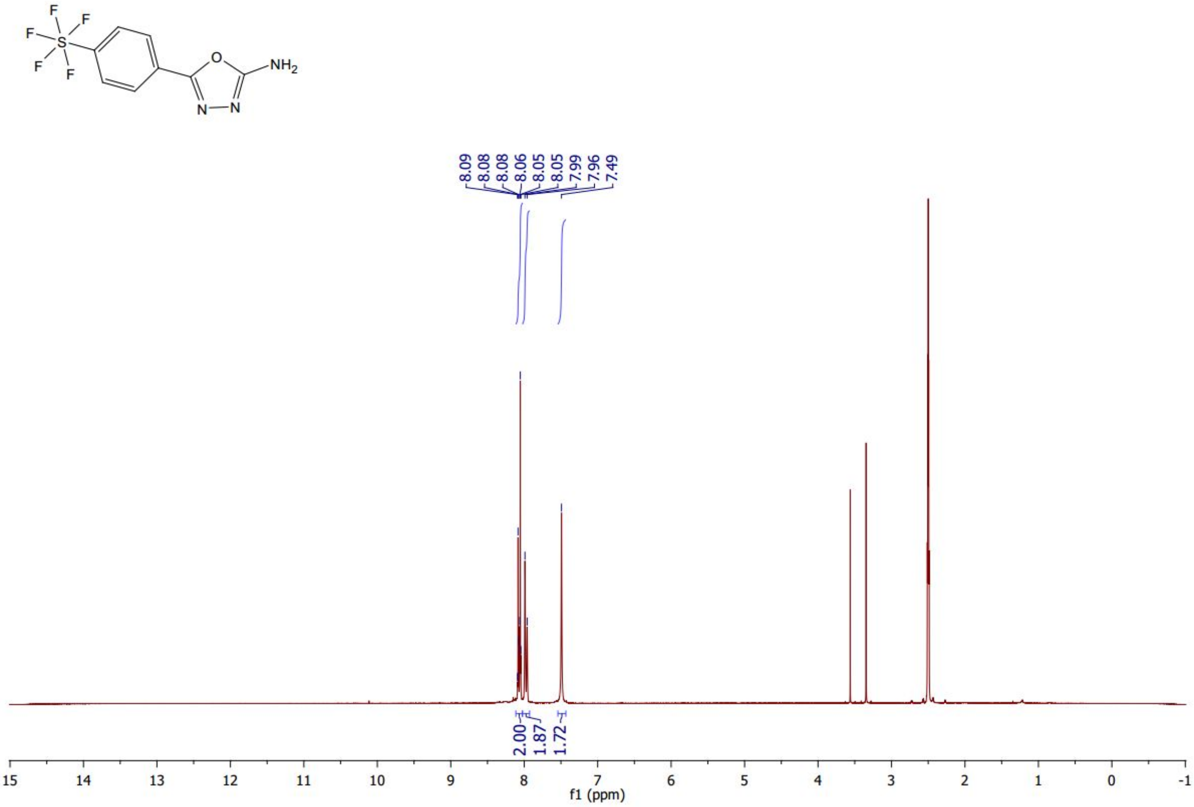

Figure S20: ^{1}H-NMR (300 MHz, DMSO-d_6) of compound **7d**

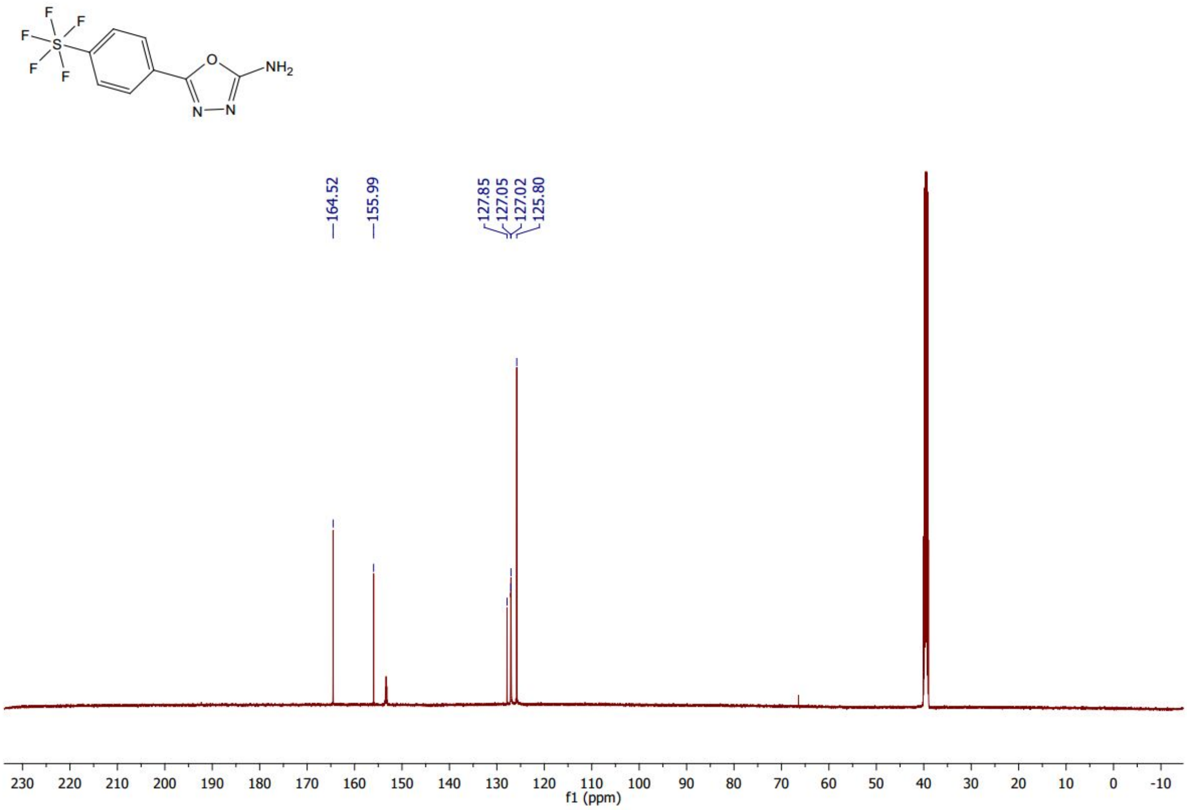

Figure S21: ^{13}C-NMR (125 MHz, DMSO-d$_6$) of compound **7d**

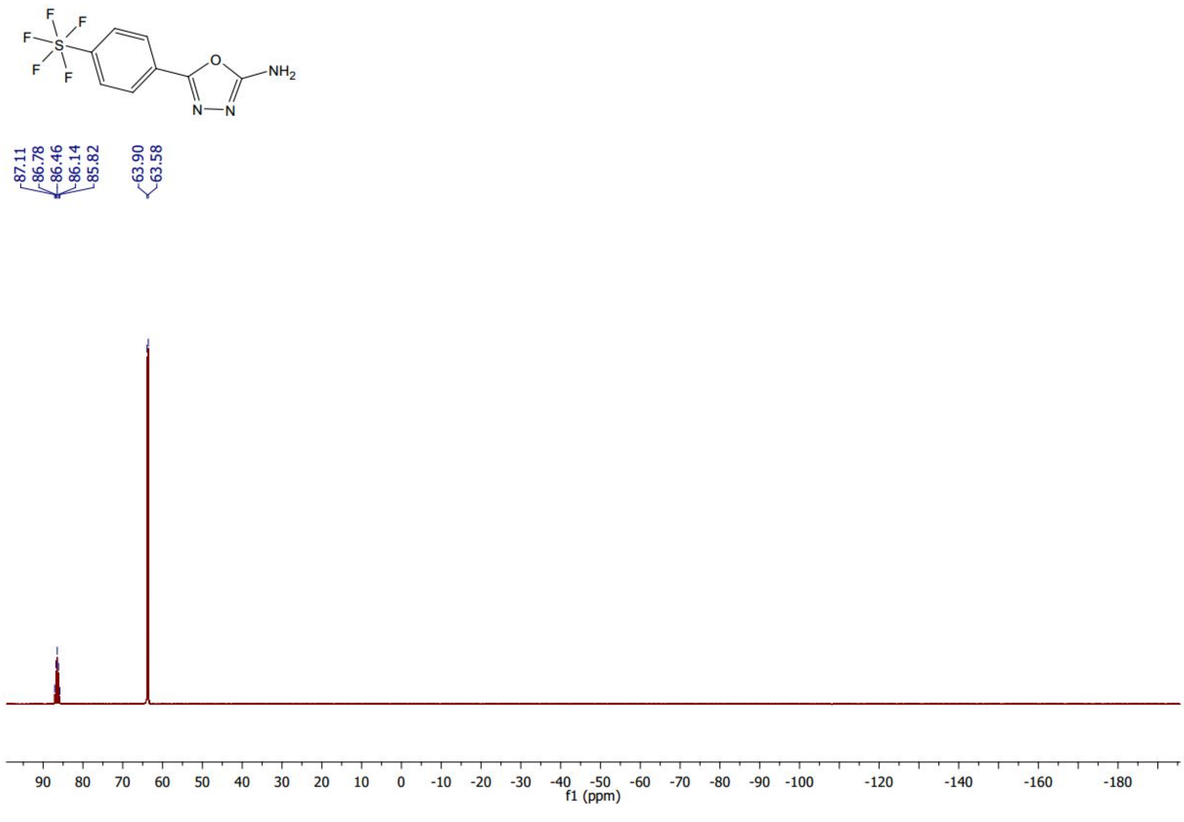

Figure S22: ^{19}F-NMR (470 MHz, DMSO-d$_6$) of compound **7d**

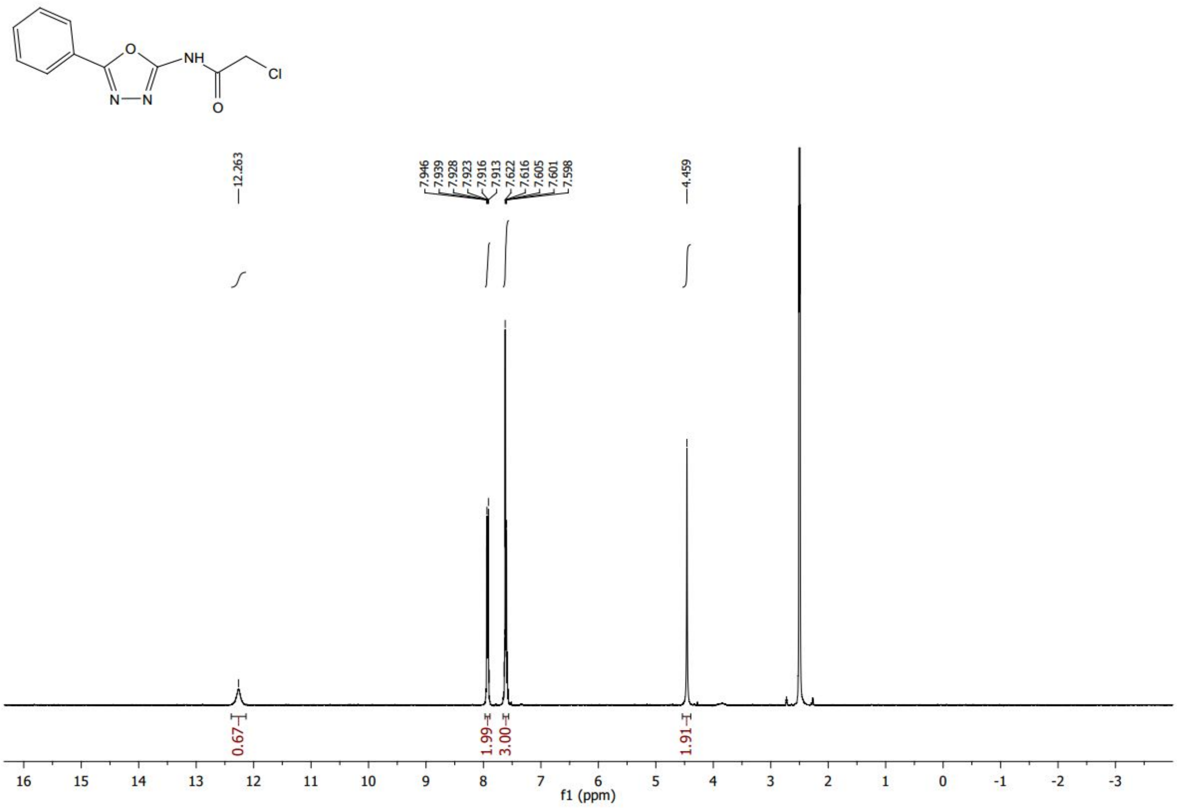

Figure S23: ^{1}H-NMR (300 MHz, DMSO-d_6) of compound **8a**

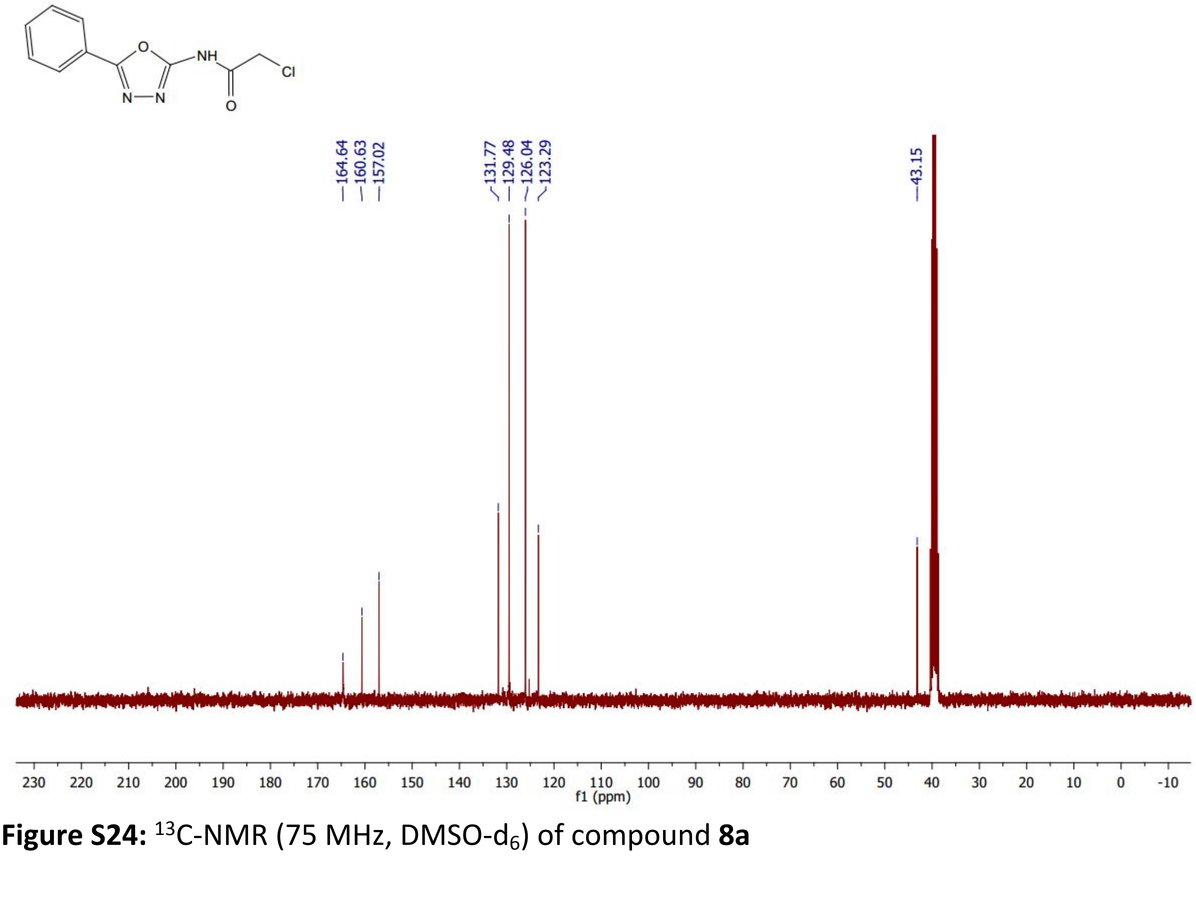

Figure S24: ^{13}C-NMR (75 MHz, DMSO-d_6) of compound **8a**

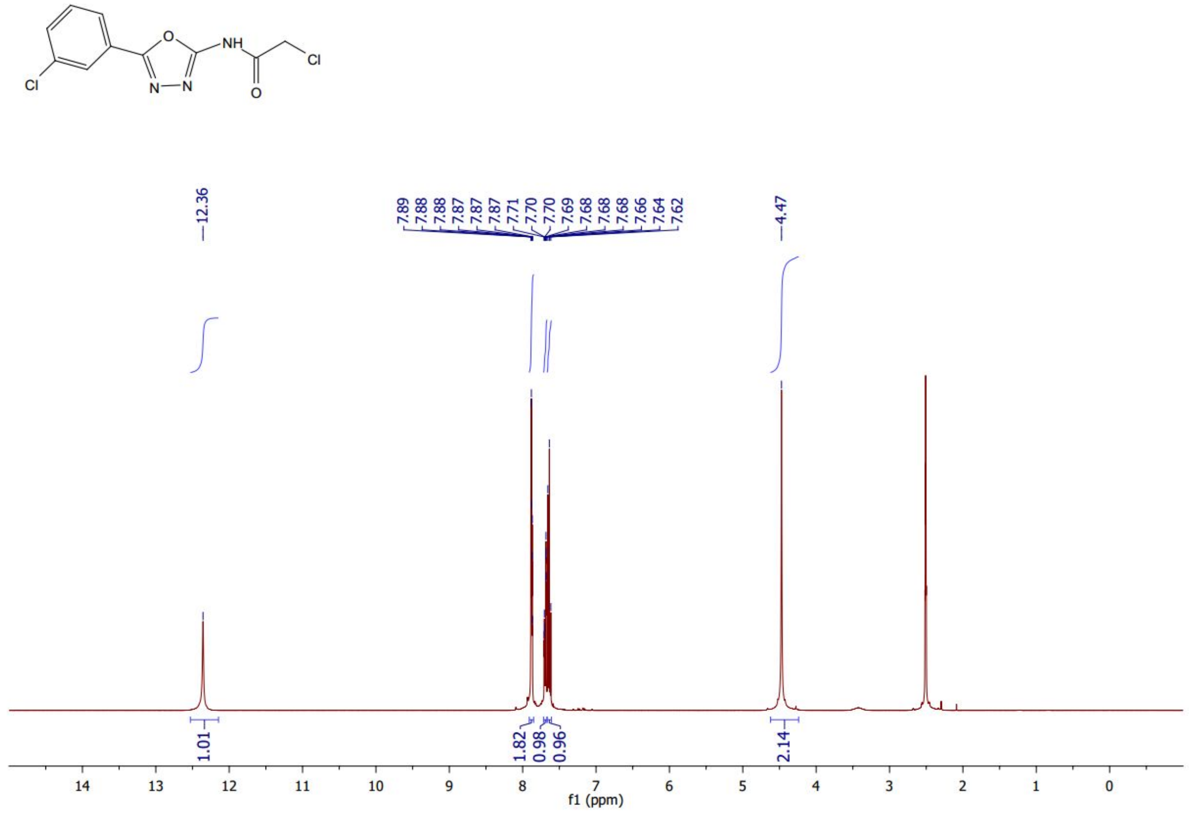

Figure S25: ^{1}H-NMR (400 MHz, DMSO-d_6) of compound **8b**

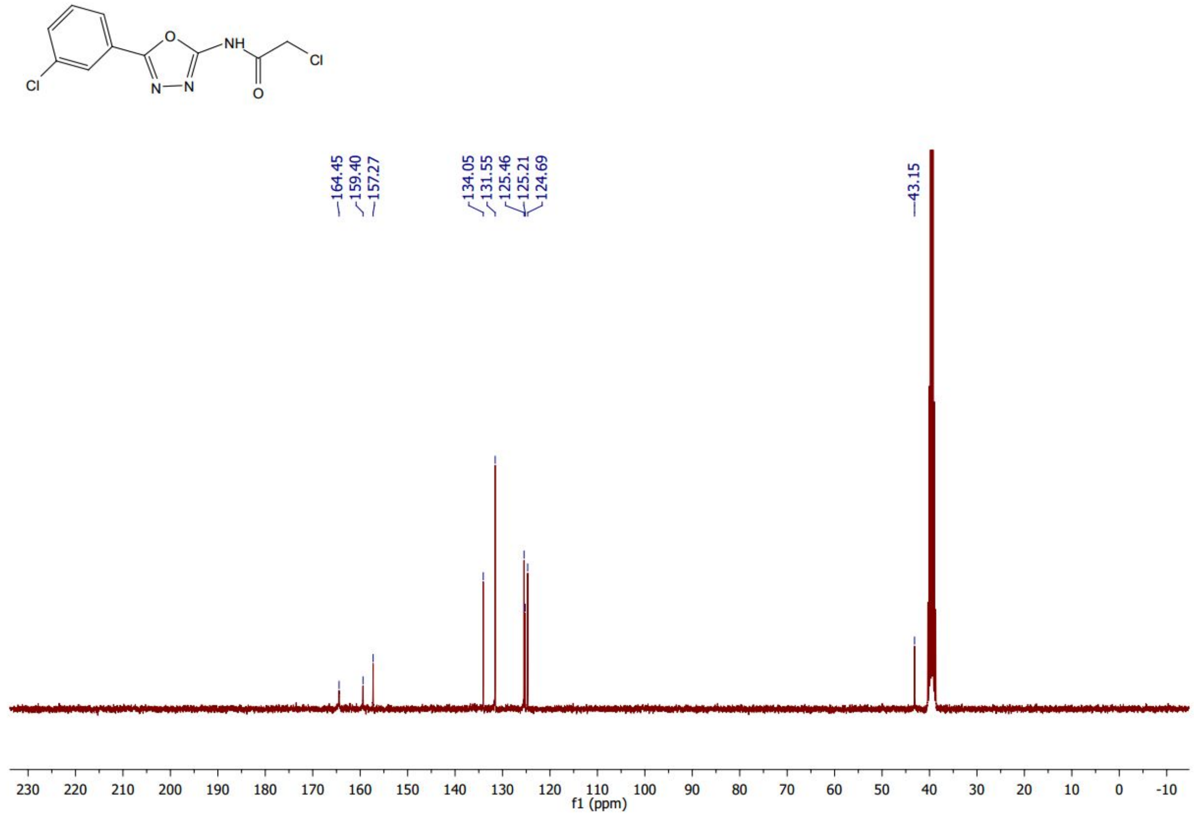

Figure S26: ^{13}C-NMR (75 MHz, DMSO-d_6) of compound **8b**

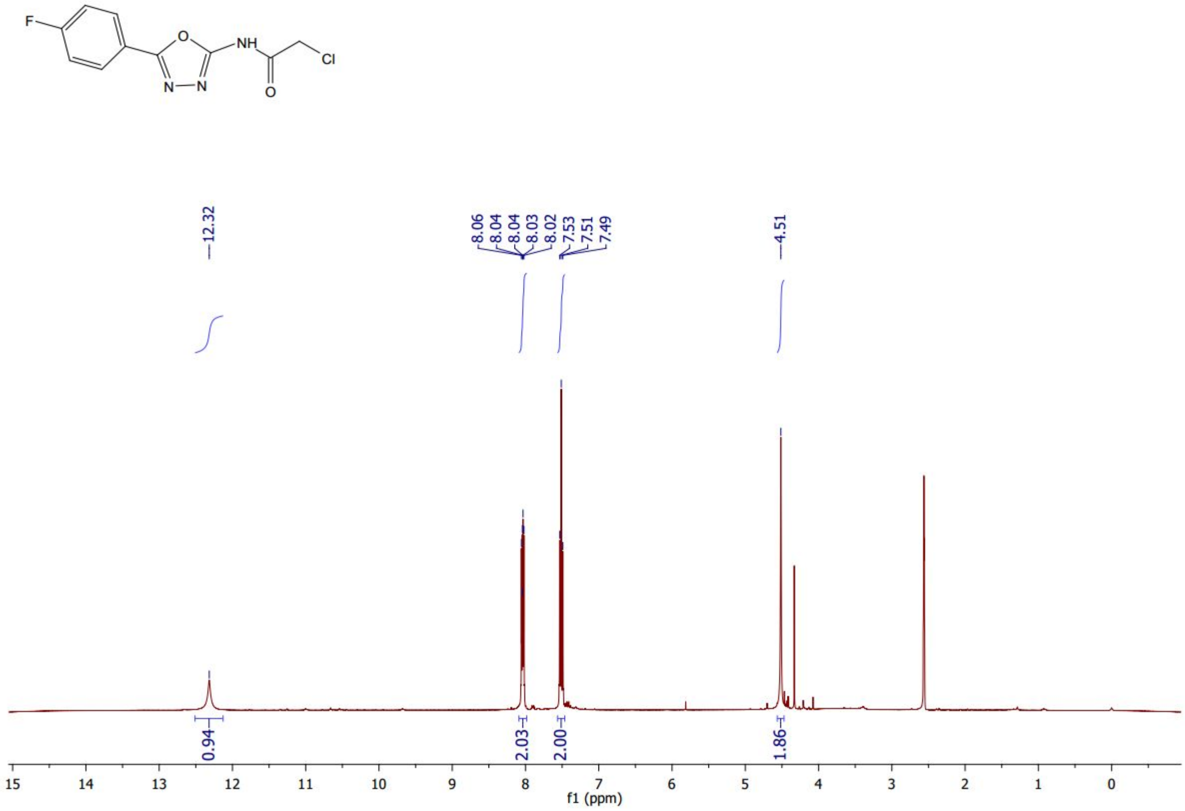

Figure S27: ^{1}H-NMR (400 MHz, DMSO-d_6) of compound **8c**

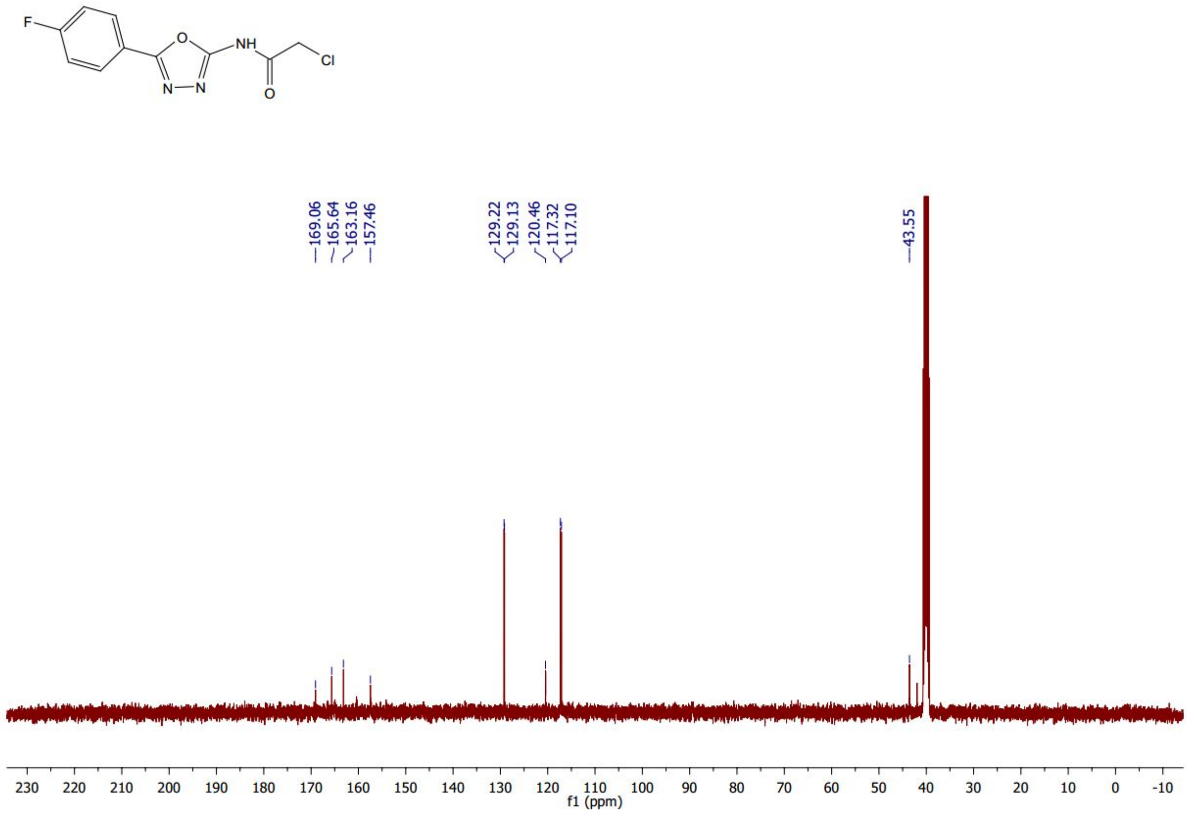

Figure S28: ^{13}C-NMR (100 MHz, DMSO-d_6) of compound **8c**

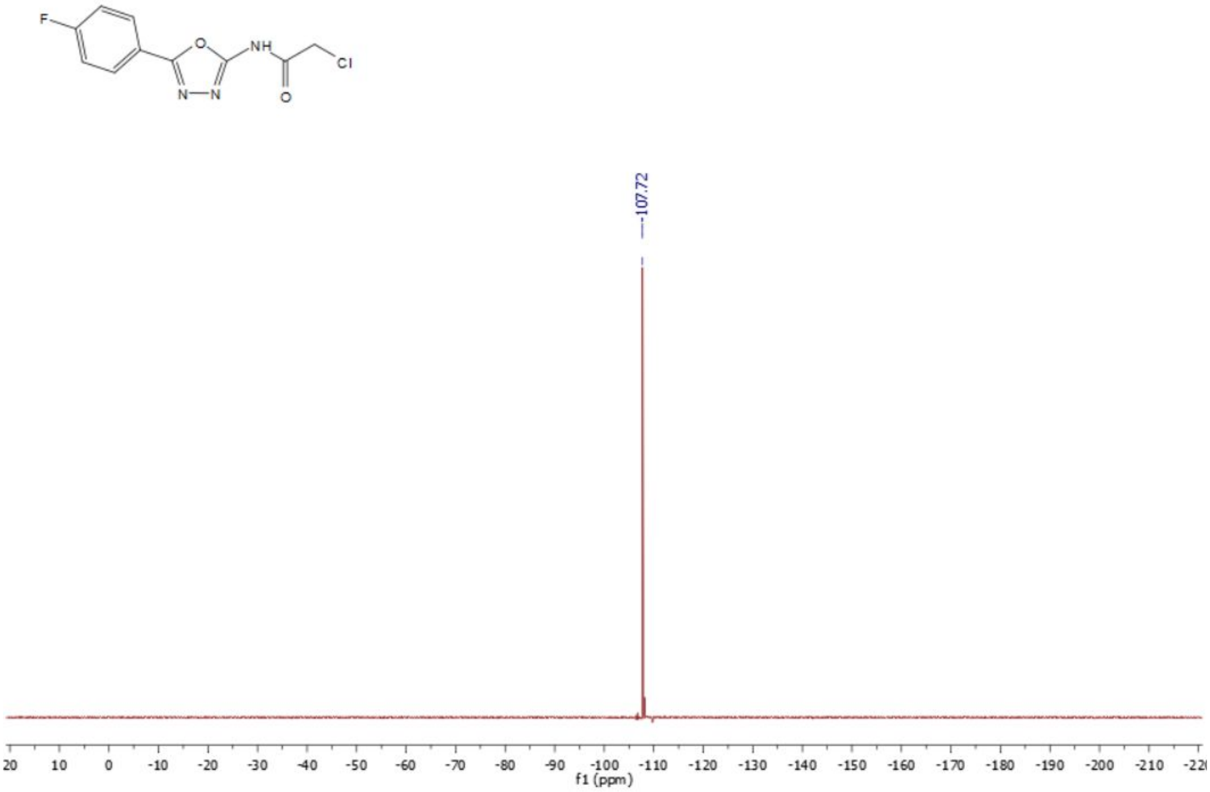

Figure S29: ^{19}F-NMR (376 MHz, DMSO-d_6) of compound **8c**

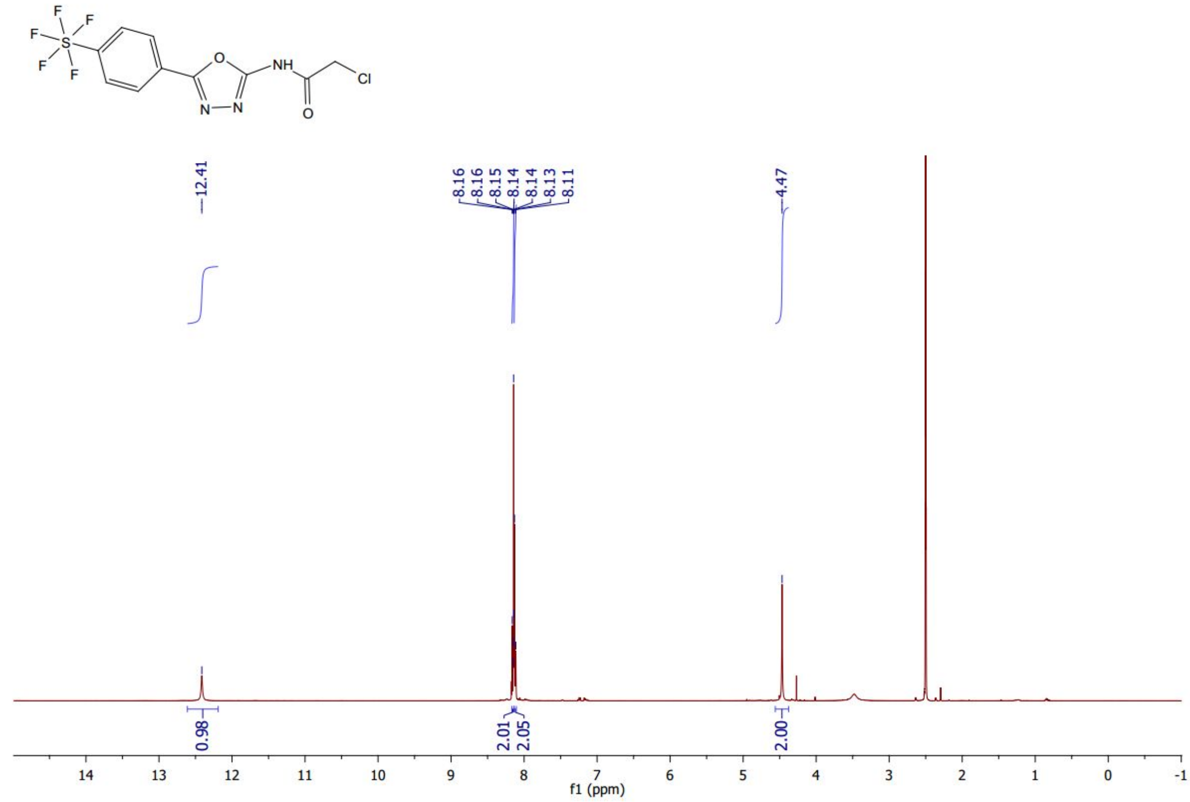

Figure S30: ^{1}H-NMR (500 MHz, DMSO-d$_6$) of compound **8d**

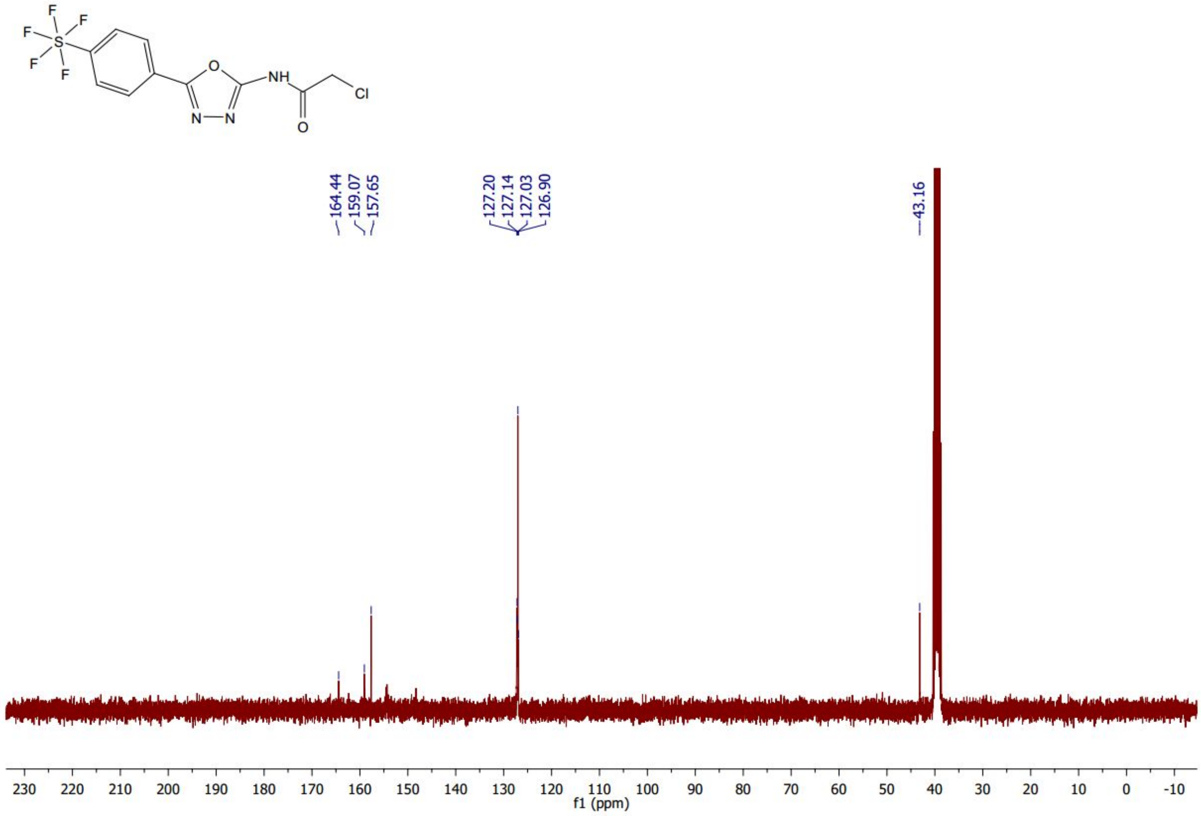

Figure S31: ^{13}C-NMR (75 MHz, DMSO-d$_6$) of compound **8d**

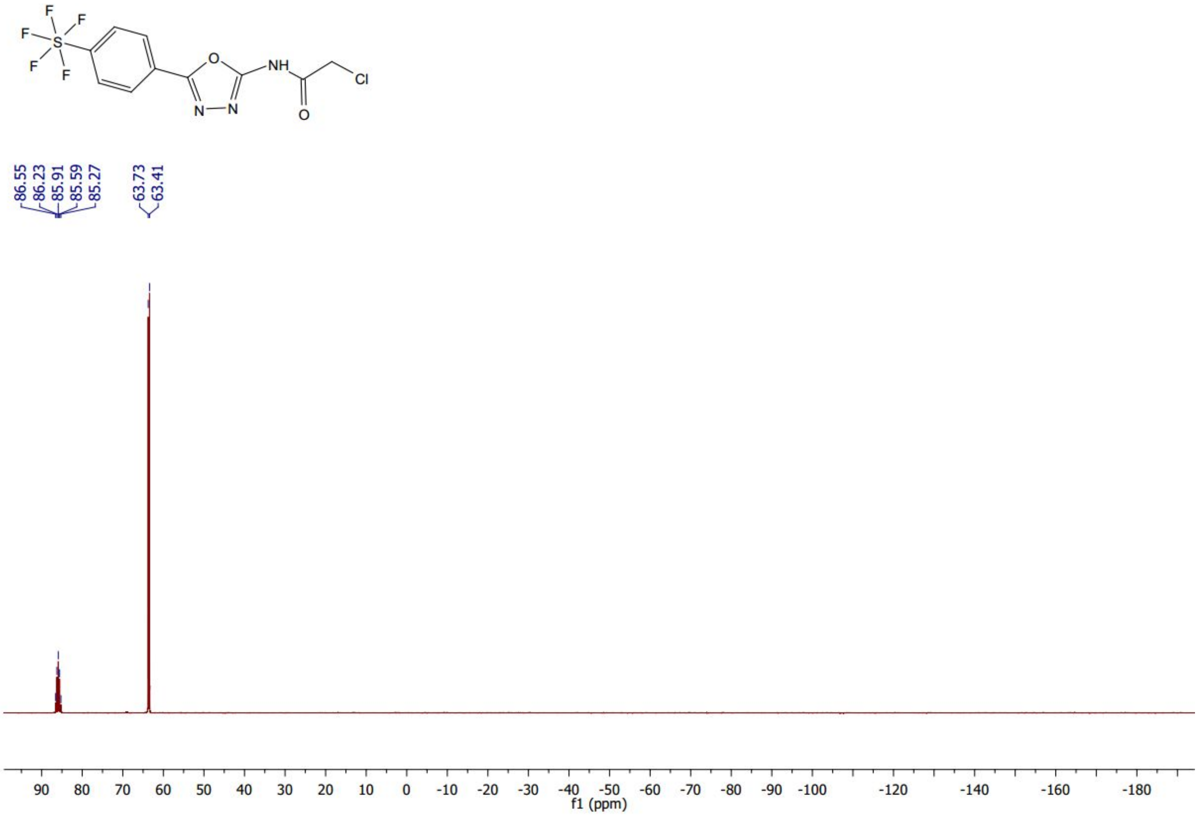

Figure S32: ^{19}F-NMR (470 MHz, DMSO-d_6) of compound **8d**

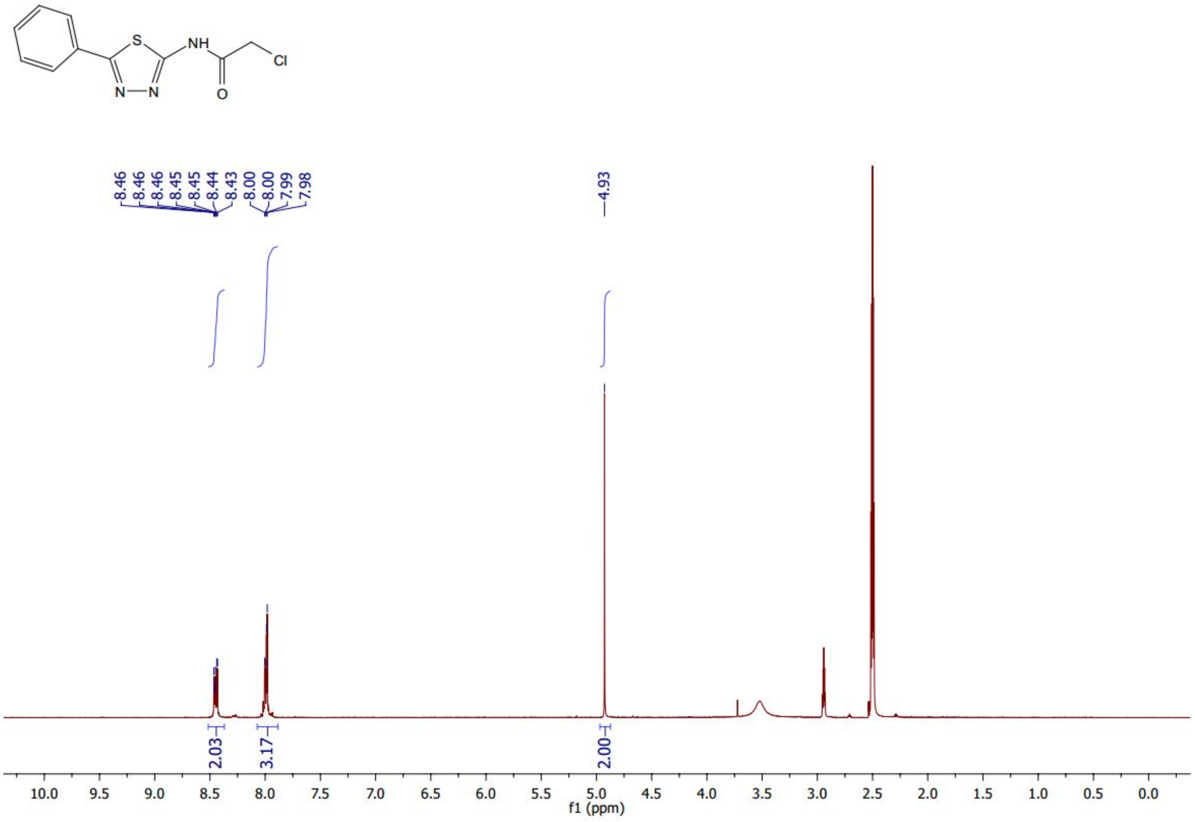

Figure S33: ^{1}H-NMR (300 MHz, DMSO-d_6) of compound **8e**

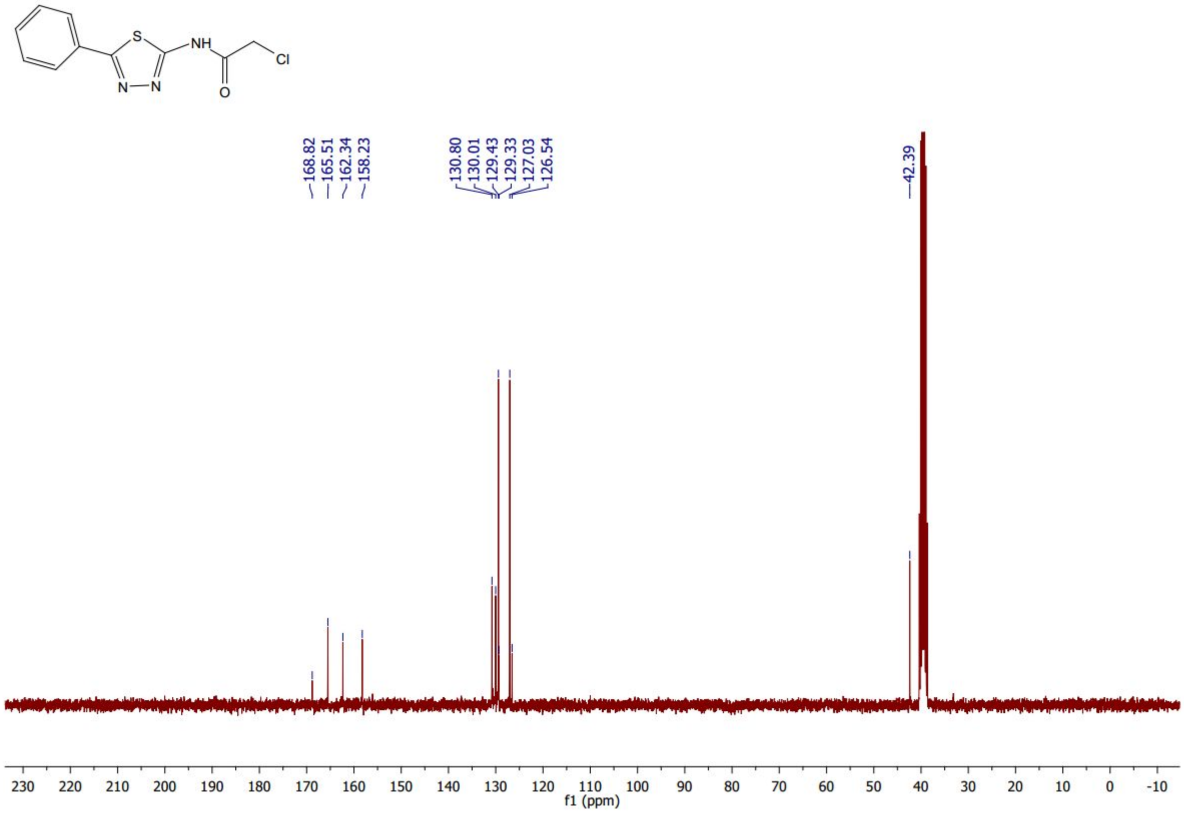

Figure S34: ^{13}C-NMR (75 MHz, DMSO-d_6) of compound **8e**

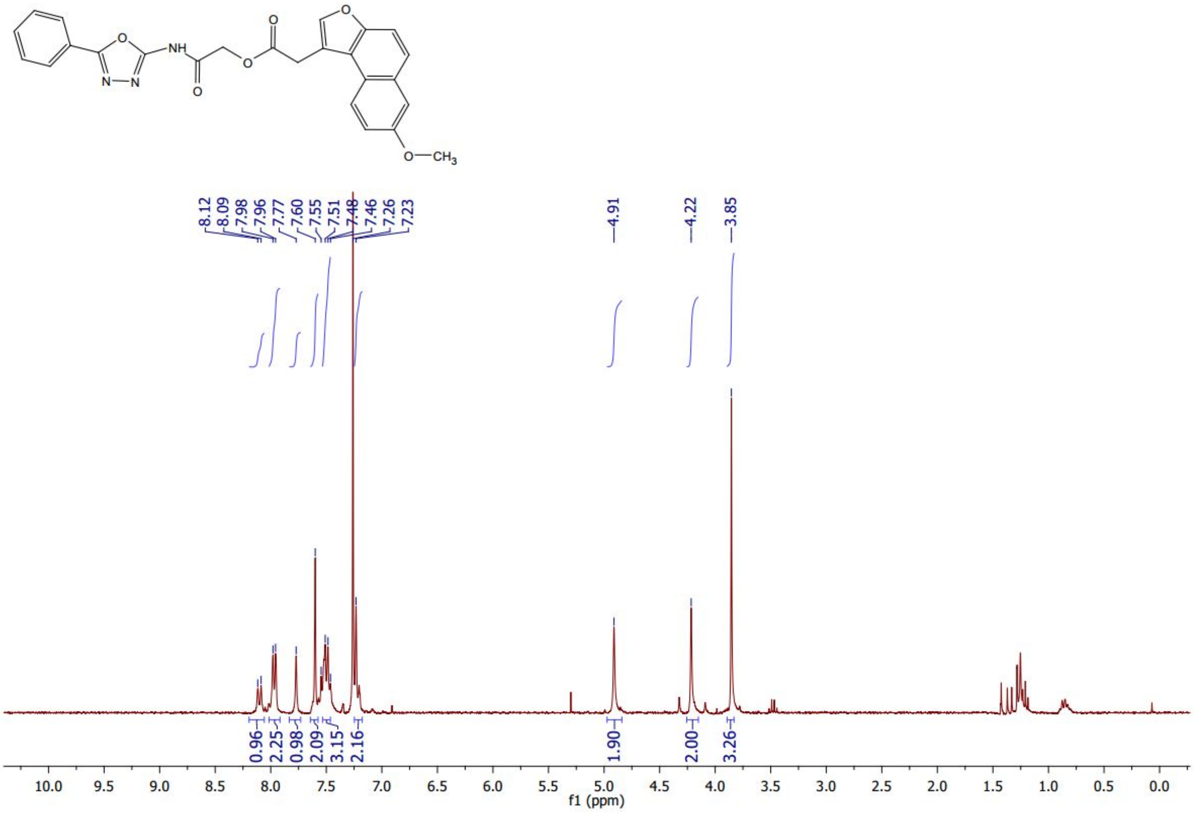

Figure S35: ^{1}H-NMR (300 MHz, $CDCl_3$) of compound **9**

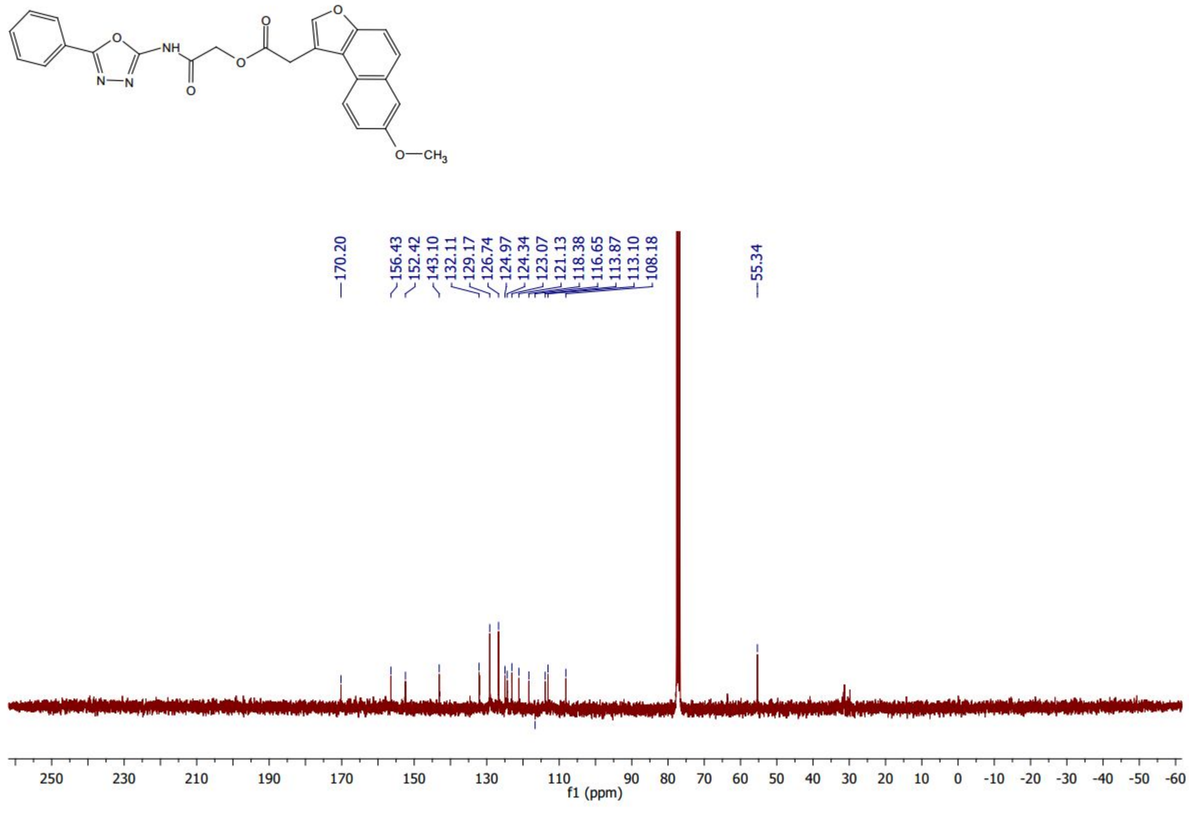

Figure S36: ^{13}C-NMR (75 MHz, $CDCl_3$) of compound **9**

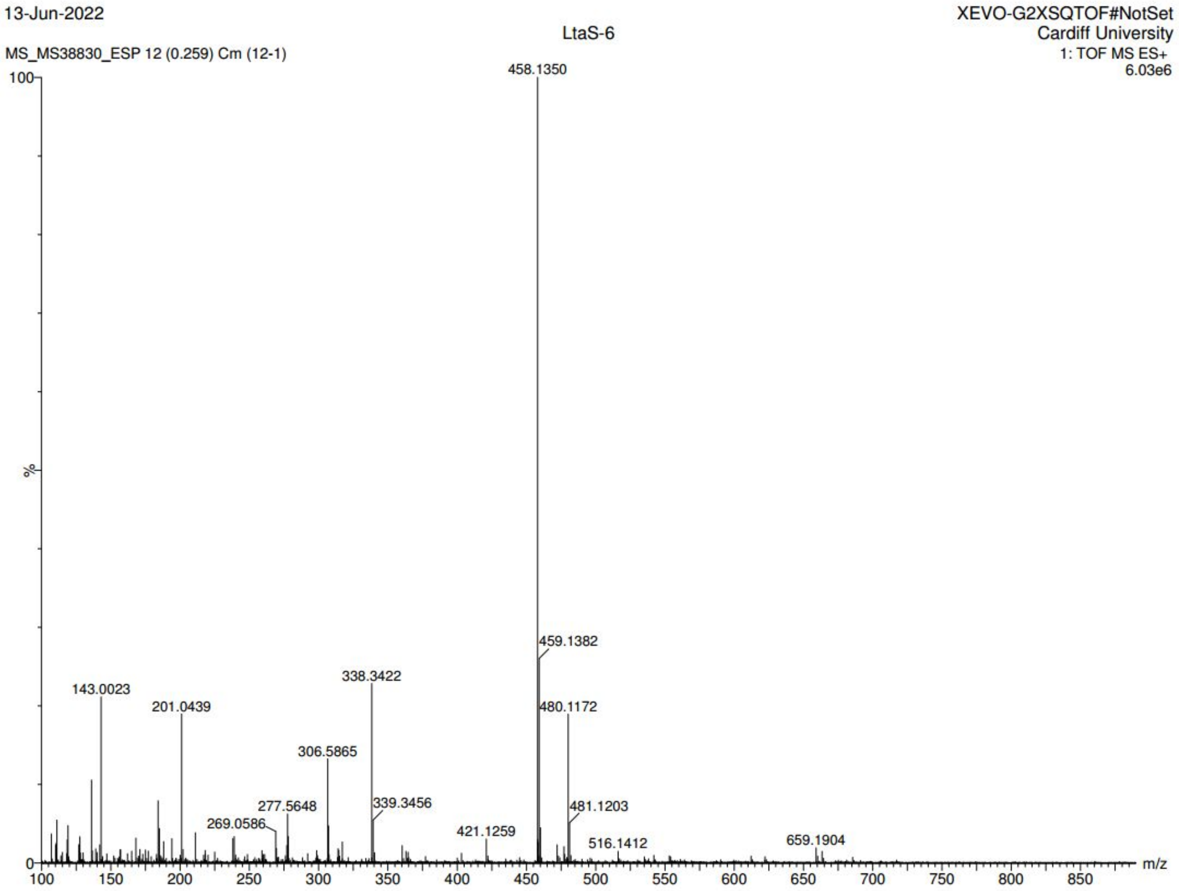

Figure S37: HR-MS of compound **9**

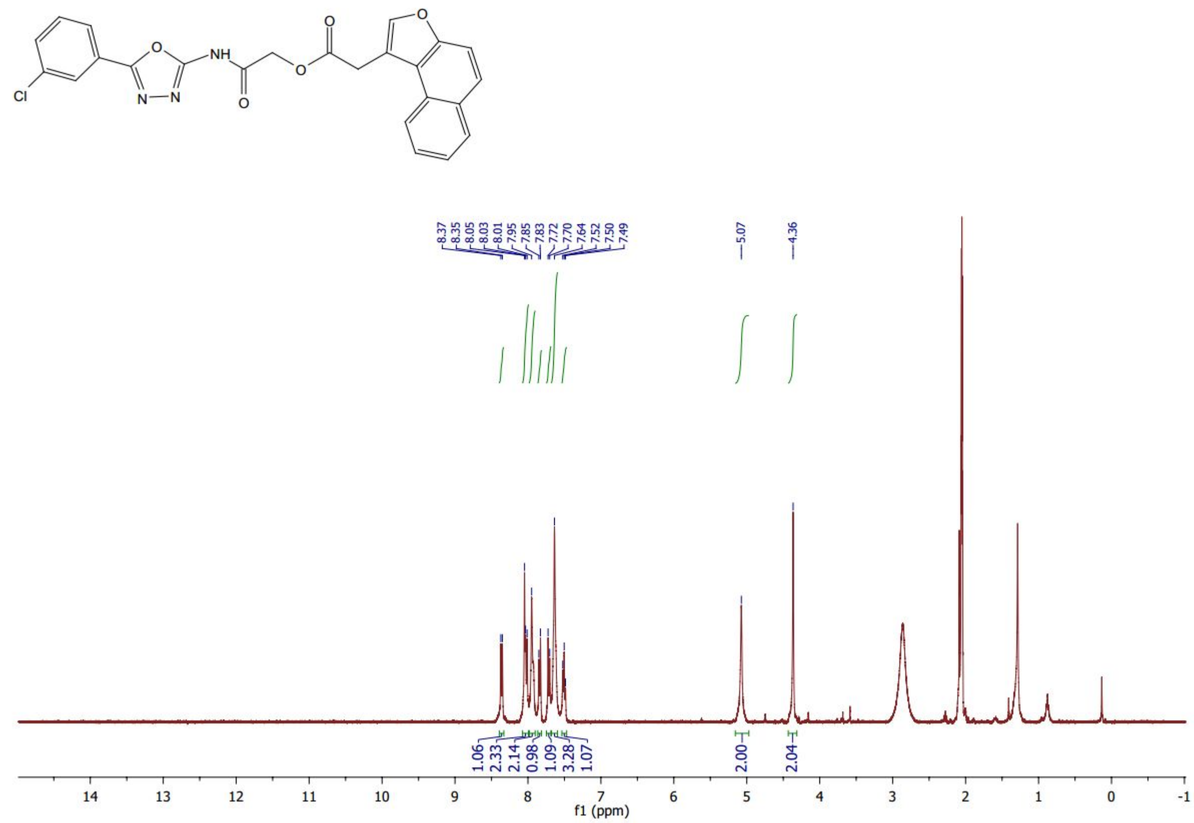

Figure S38: ^{1}H-NMR (400 MHz, acetone-d$_6$) of compound **10**

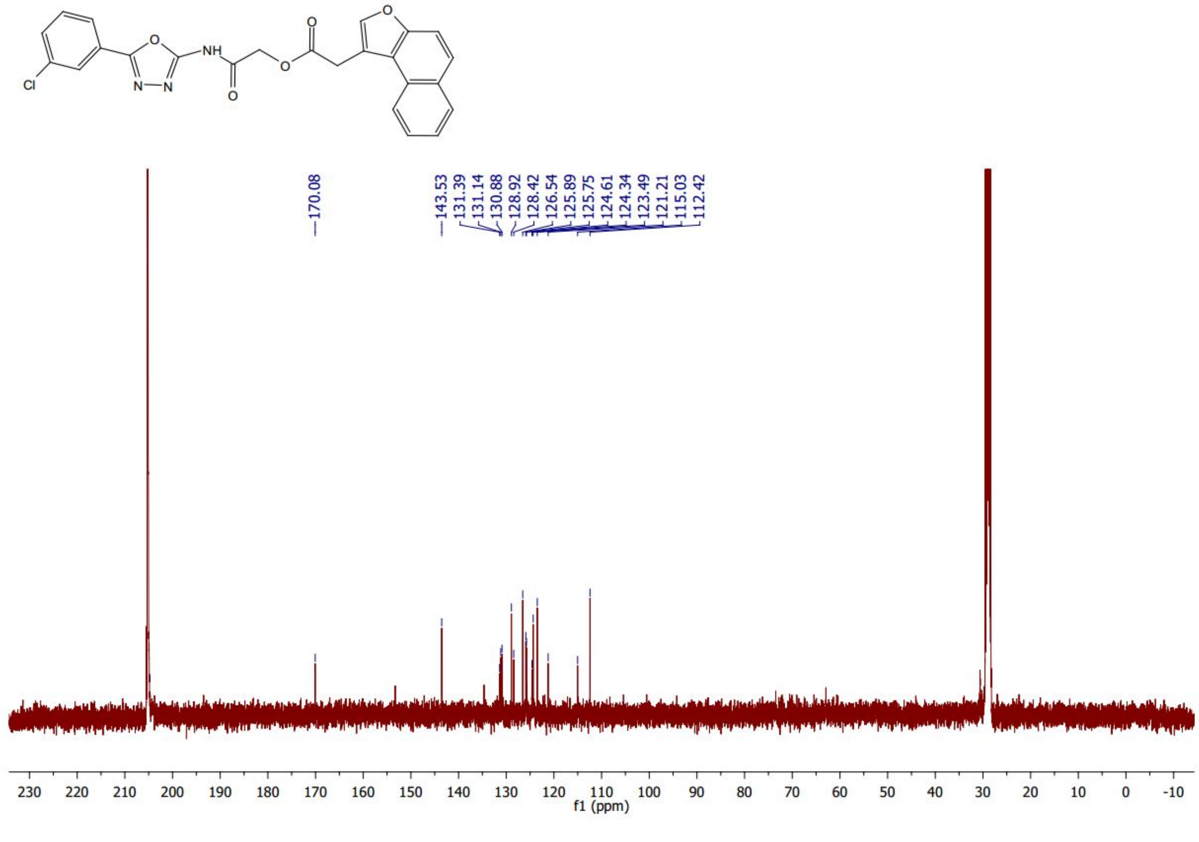

Figure S39: ^{13}C-NMR (100 MHz, acetone-d$_6$) of compound **10**

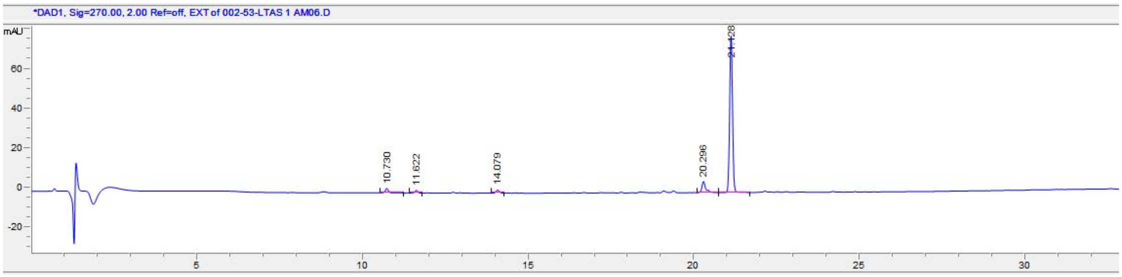

#	Time	Area	Height	Width	Area%	Symmetry
1	10.73	14.5	2.3	0.0984	2.592	0.829
2	11.622	7.6	1.3	0.0899	1.360	1.03
3	14.079	11.1	1.6	0.1112	1.981	0.954
4	20.296	44.8	5.7	0.1159	8.028	0.627
5	21.128	480.4	79.4	0.0946	86.039	0.934

Figure S40: HPLC trace of compound **10**

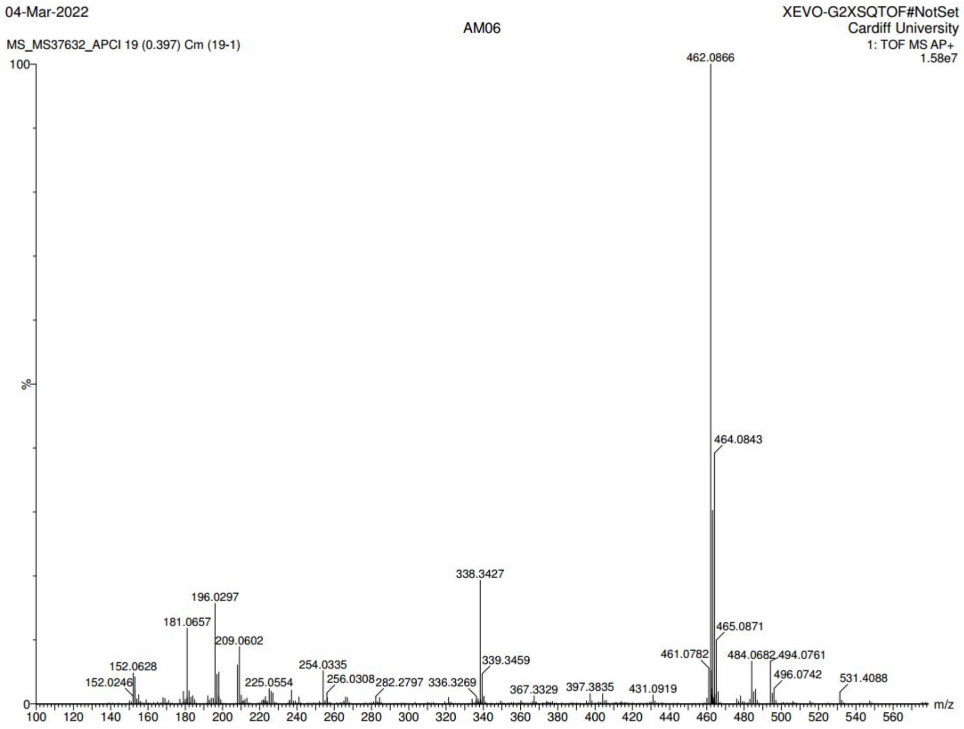

Figure S41: HR-MS of compound **10**

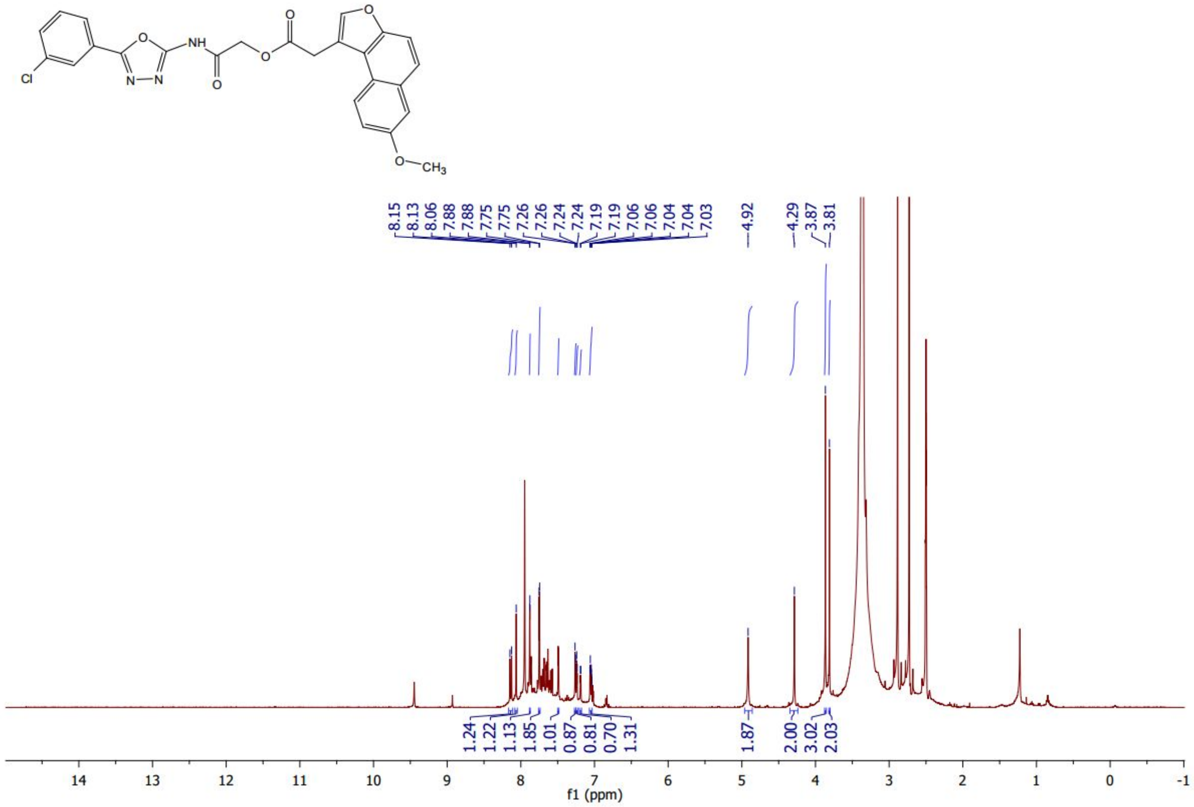

Figure S42: ^{1}H-NMR (400 MHz, DMSO-d_6) of compound **11**

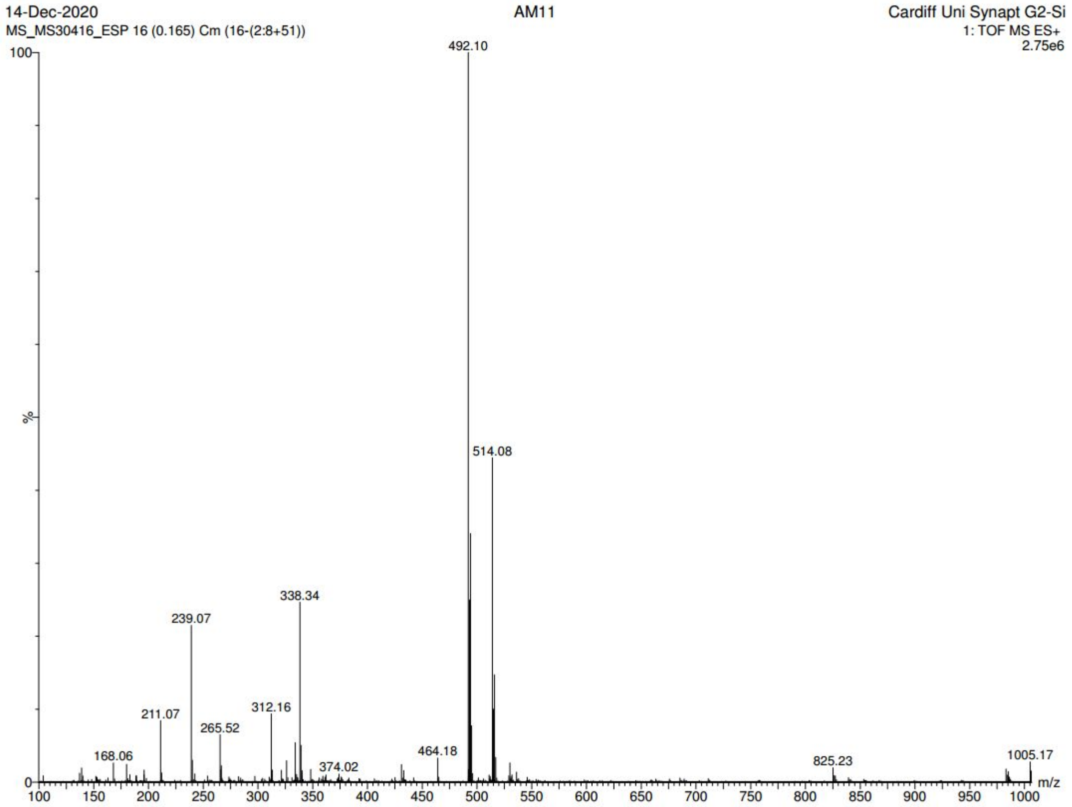

Figure S43: LR-MS of compound **11**

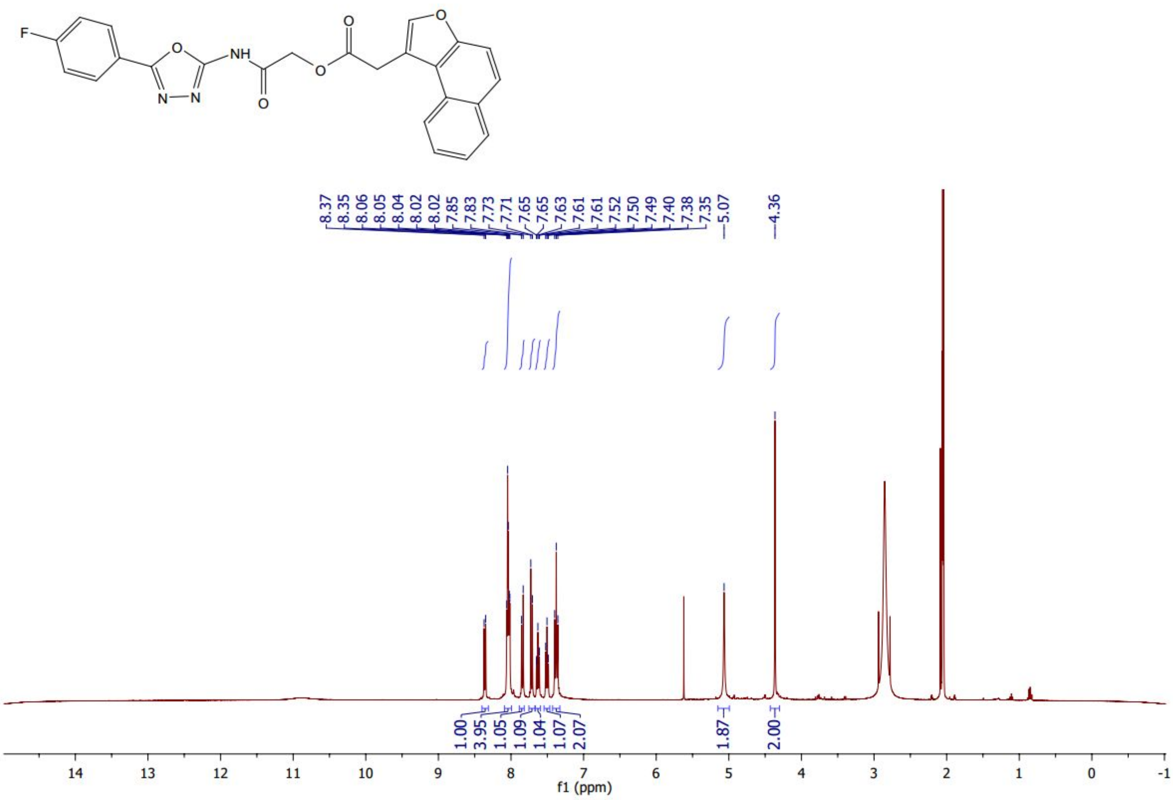

Figure S44: ^{1}H-NMR (400 MHz, acetone-d_6) of compound **12**

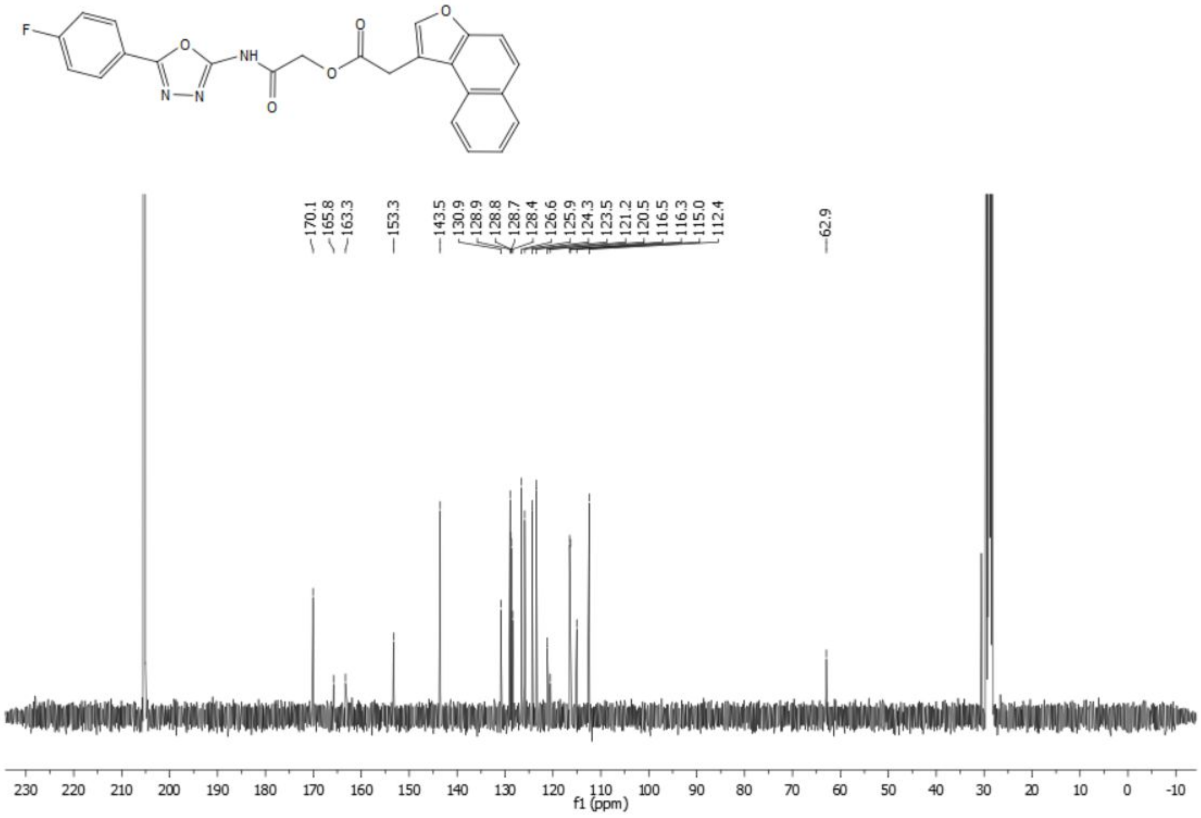

Figure S45: ^{13}C-NMR (100 MHz, acetone-d_6) of compound **12**

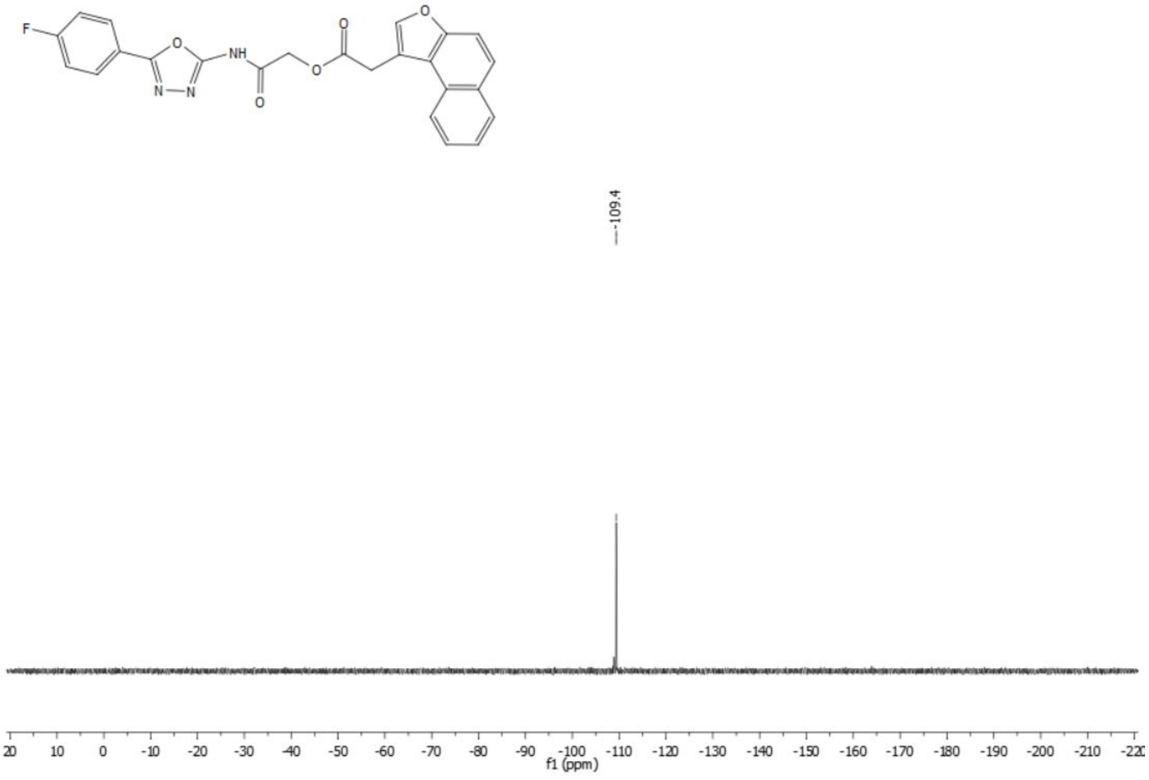

Figure S46: ^{19}F-NMR (376 MHz, acetone-d_6) of compound **12**

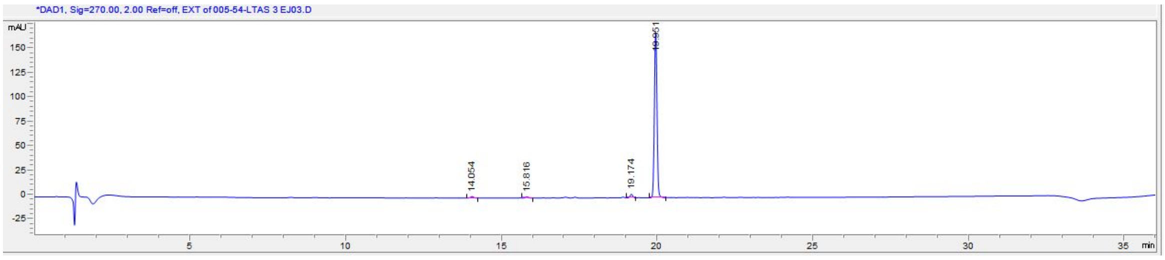

#	Time	Area	Height	Width	Area%	Symmetry
1	14.054	10.9	1.5	0.1108	1.061	0.935
2	15.816	7.6	1.3	0.0879	0.734	0.939
3	19.174	21.8	3.7	0.0889	2.116	1.006
4	19.951	988.9	169.6	0.088	96.089	0.939

Figure S47: HPLC trace of compound **12**

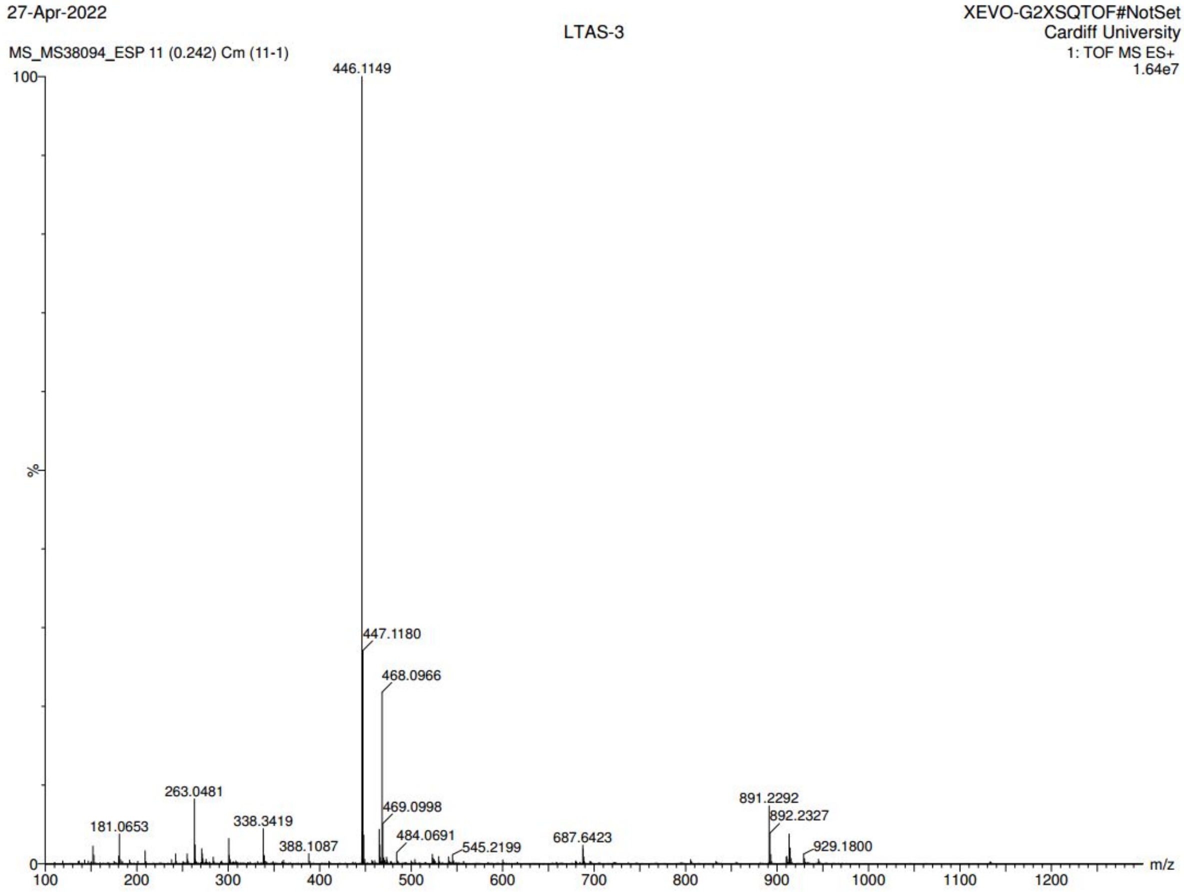

Figure S48: HR-MS of compound **12**

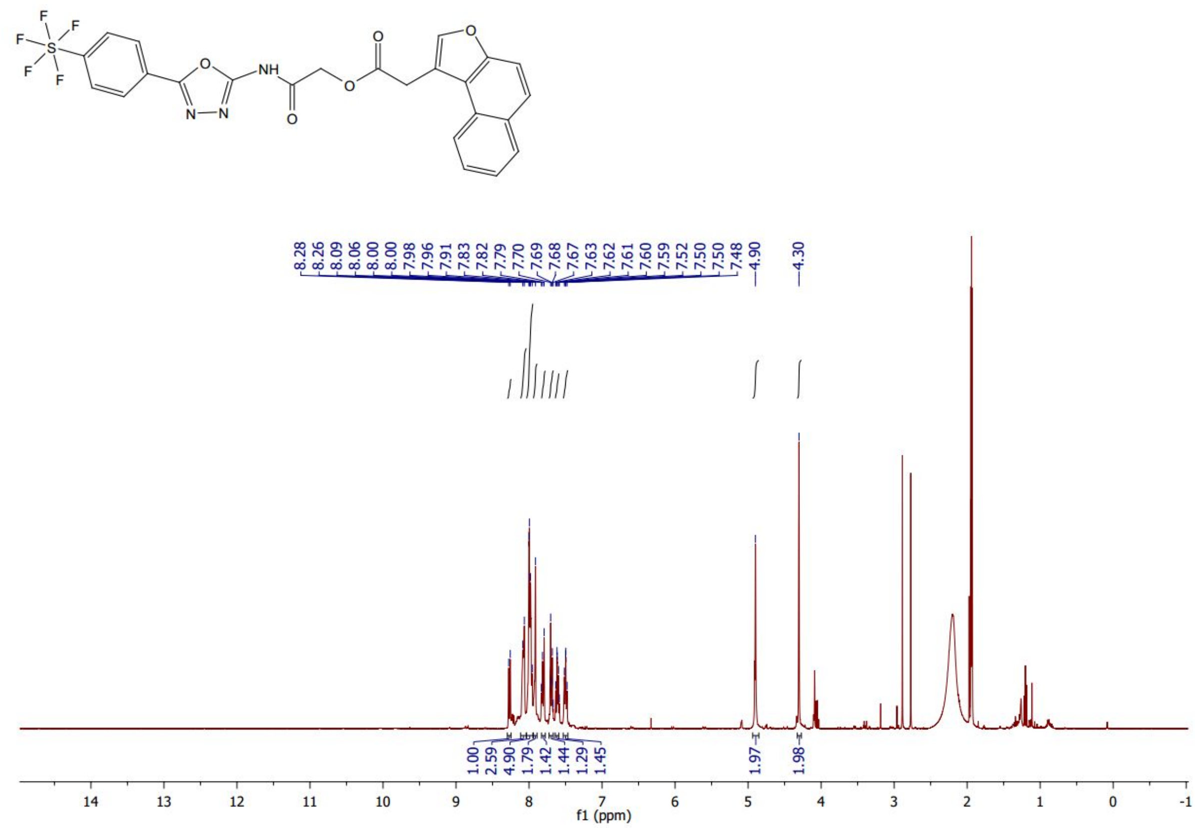

Figure S49: ^{1}H-NMR (400 MHz, acetone-d_6) of compound **13**

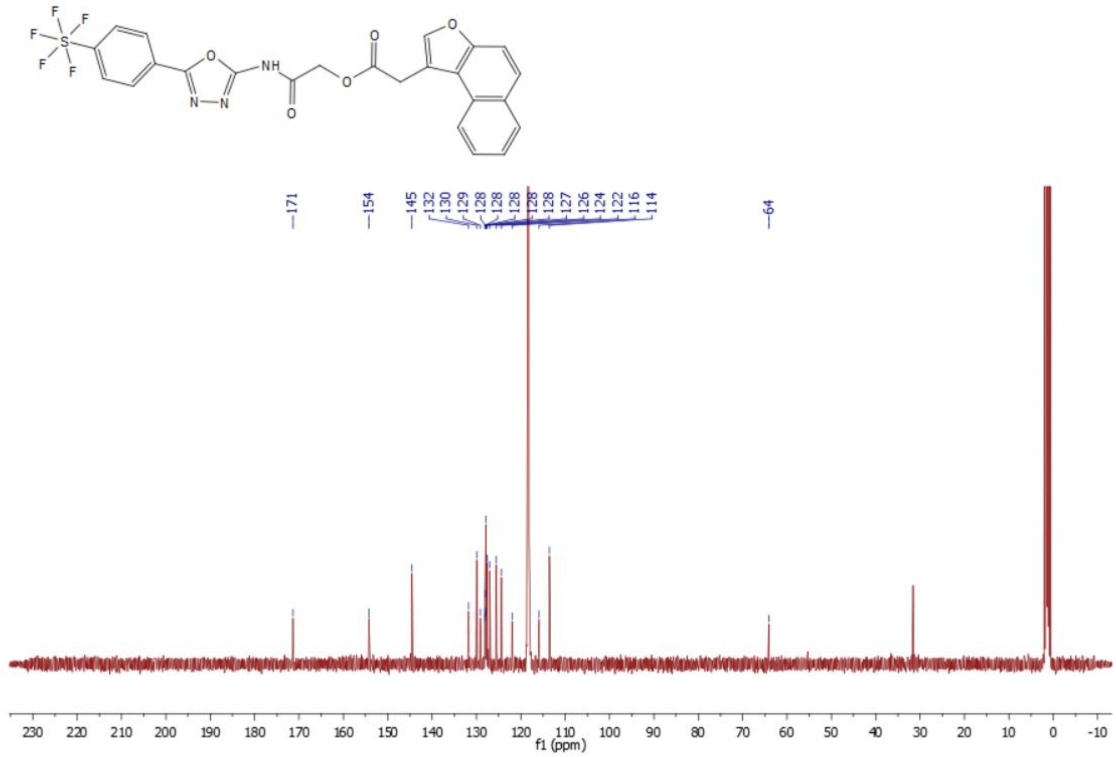

Figure S50: ^{13}C-NMR (100 MHz, acetone-d_6) of compound **13**

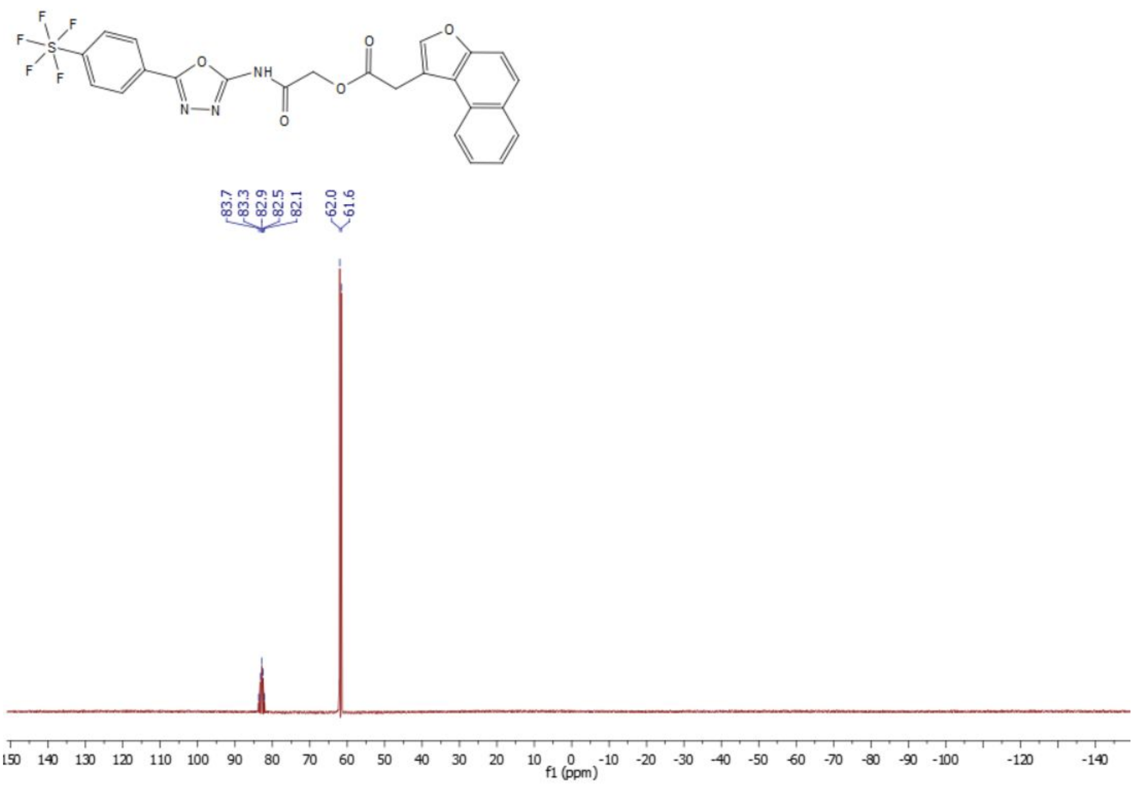

Figure S51: ^{19}F-NMR (376 MHz, acetone-d$_6$) of compound **13**

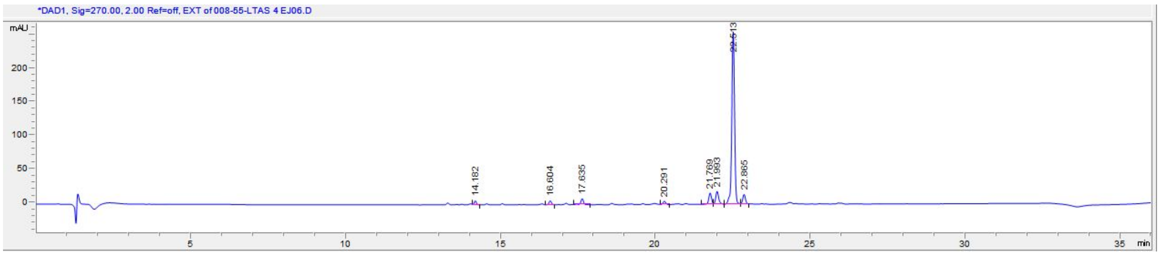

#	Time	Area	Height	Width	Area%	Symmetry
1	14.182	30.3	5.8	0.0805	1.460	0.988
2	16.604	32.4	5.8	0.0893	1.561	0.948
3	17.635	54	8.8	0.0955	2.605	1.18
4	20.291	29.2	5	0.0926	1.409	0.988
5	21.769	106	16.8	0.0975	5.108	0.998
6	21.993	127.7	19.3	0.1014	6.156	0.808
7	22.513	1608.4	256.8	0.0971	77.530	1.012
8	22.865	86.5	14.3	0.0947	4.172	0.936

Figure S52: HPLC trace of compound **13**

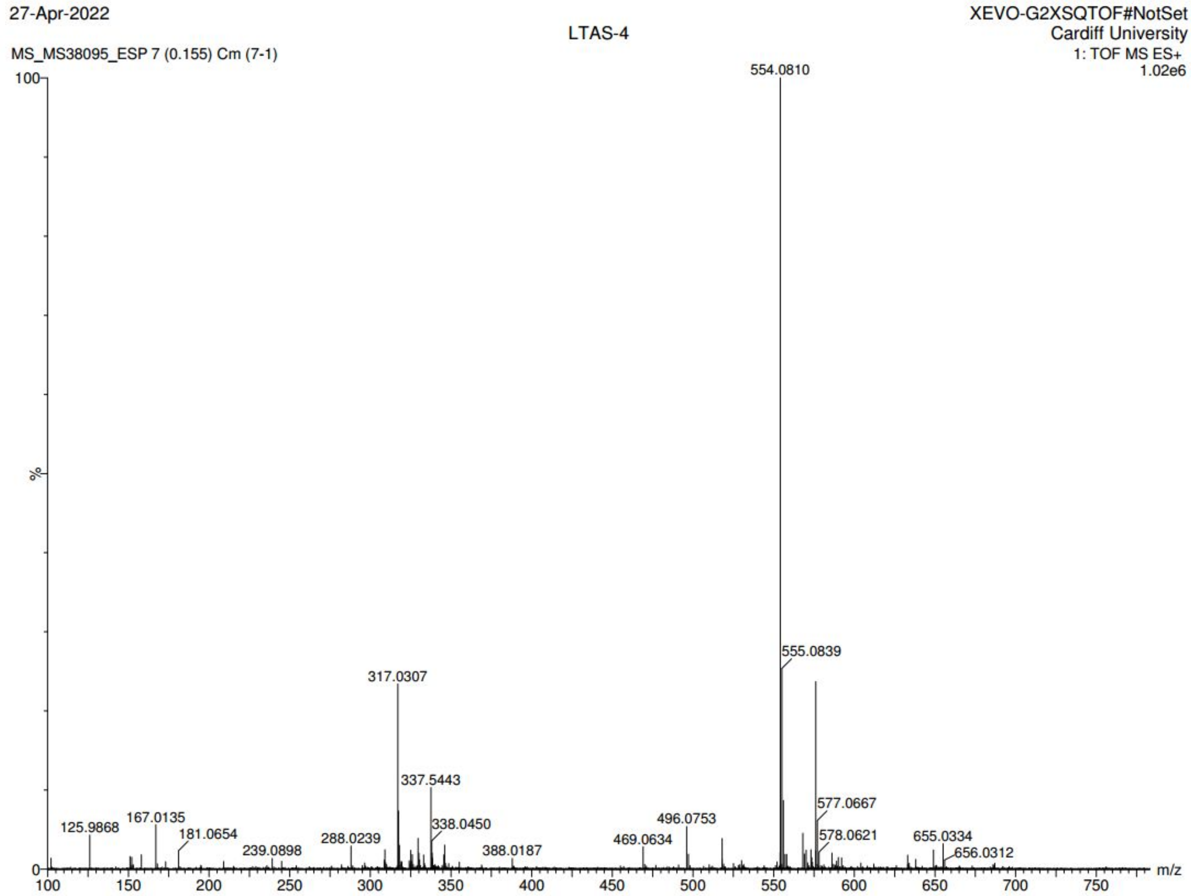

Figure S53: HR-MS of compound **13**

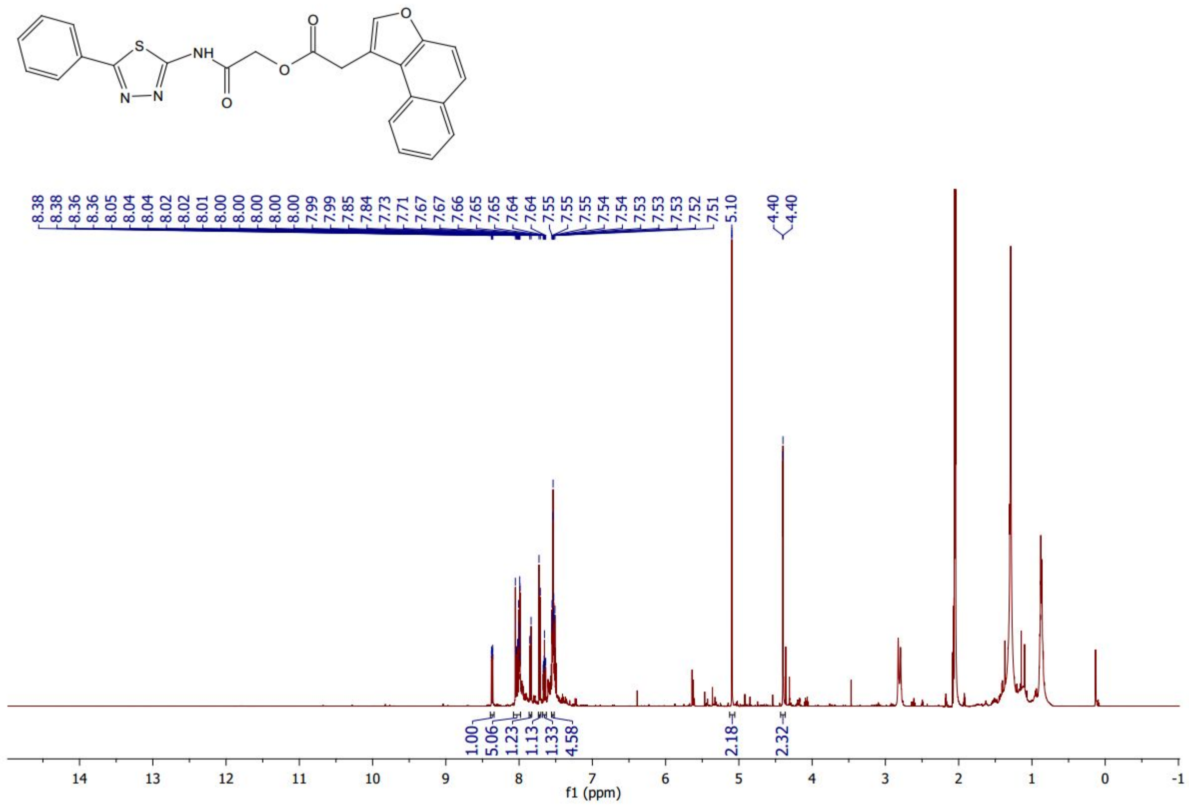

Figure S54: ^{1}H-NMR (500 MHz, acetone-d_6) of compound **14**

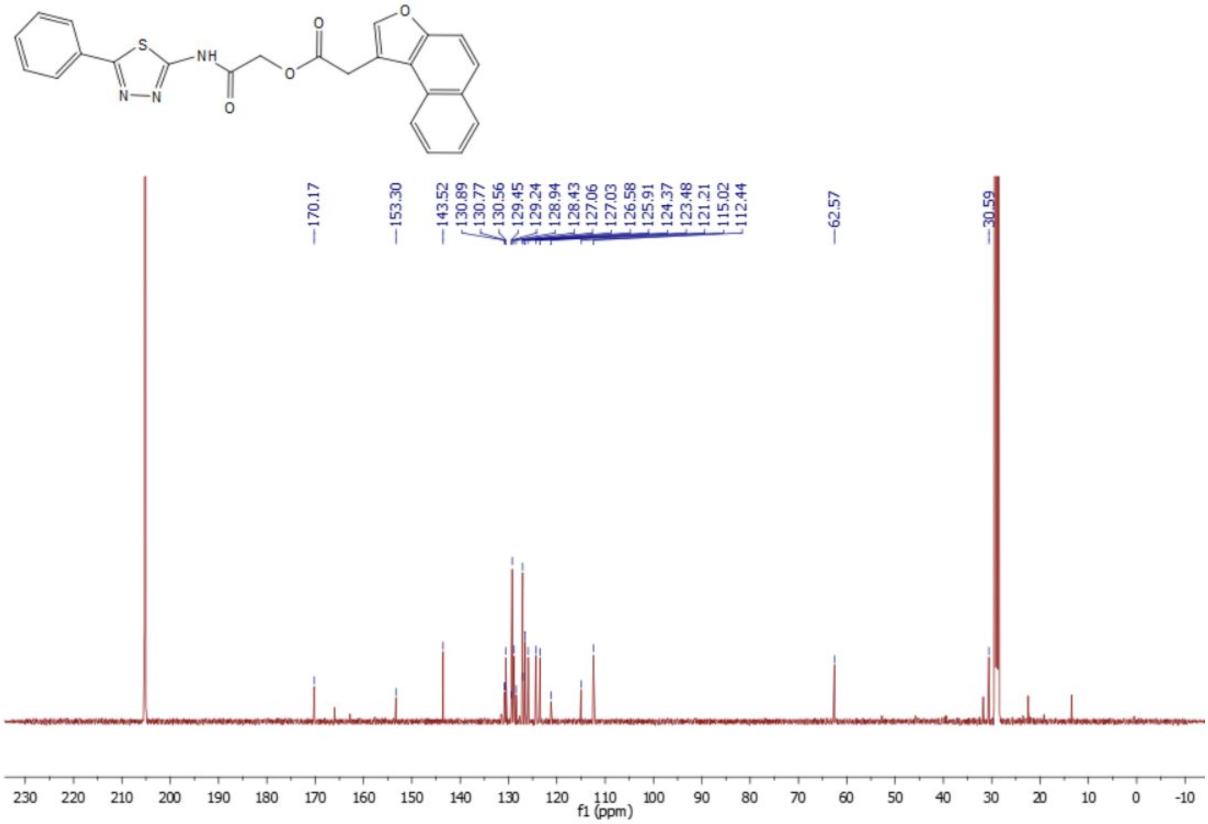

Figure S55: ^{13}C-NMR (125 MHz, acetone-d_6) of compound **14**

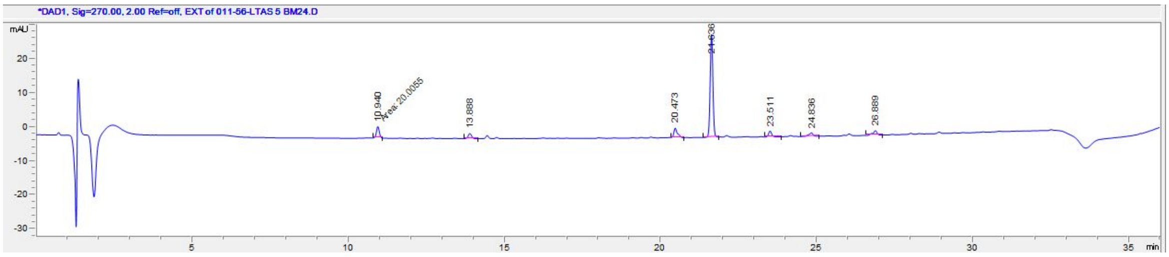

#	Time	Area	Height	Width	Area%	Symmetry
1	10.94	20	3.3	0.1017	7.369	0.962
2	13.888	11.1	1.5	0.1146	4.101	0.93
3	20.473	21.7	2.7	0.1121	7.994	0.546
4	21.636	184	30.3	0.0948	67.780	0.958
5	23.511	12.1	1.7	0.1063	4.462	0.815
6	24.836	9.9	1	0.135	3.633	1.503
7	26.889	12.7	1.3	0.1333	4.660	1.748

Figure S56: HPLC trace of compound **14**

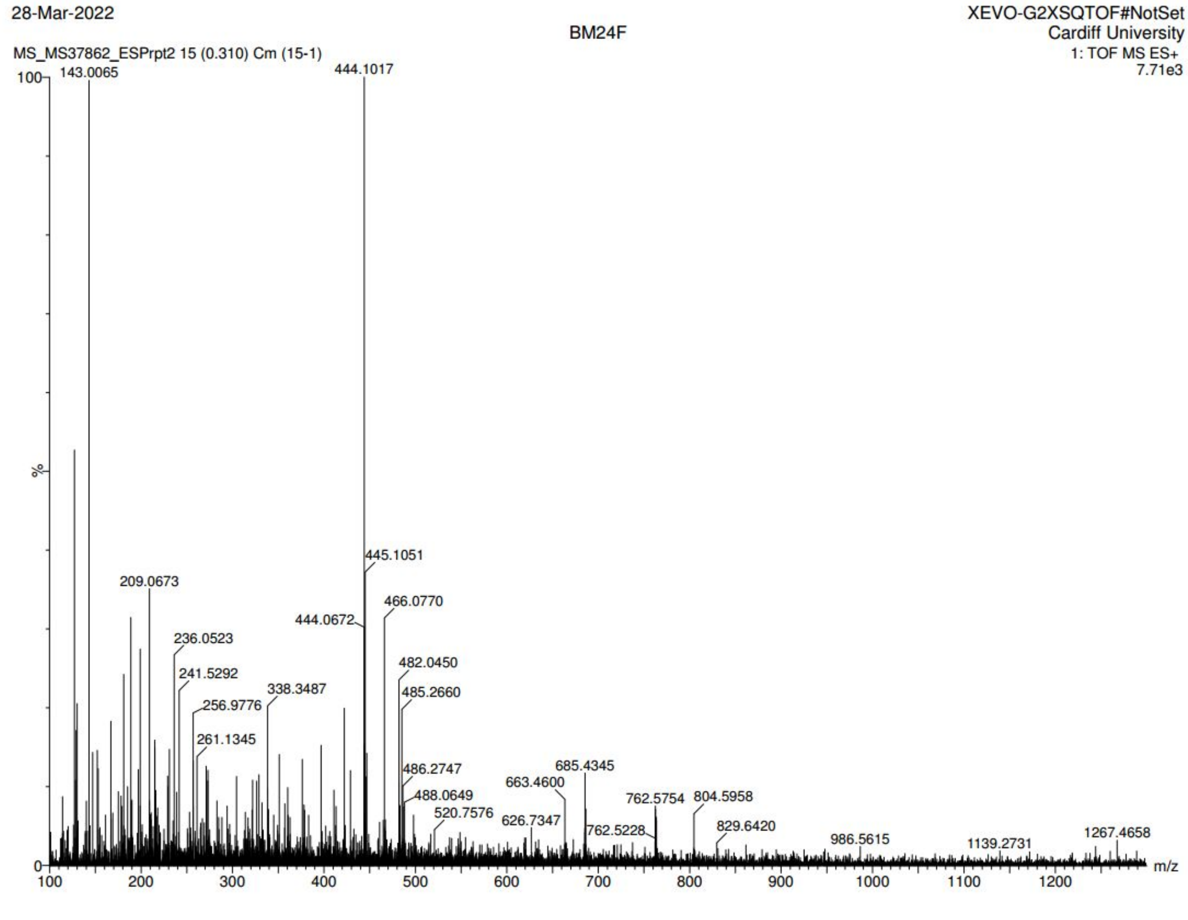

Figure S57: HR-MS of compound **14**

References

(1) Diep, B. A.; Gill, S. R.; Chang, R. F.; Phan, T. H.; Chen, J. H.; Davidson, M. G.; Lin, F.; Lin, J.; Carleton, H. A.; Mongodin, E. F.; et al. Complete genome sequence of USA300, an epidemic clone of community-acquired meticillin-resistant Staphylococcus aureus. *Lancet* **2006**, *367* (9512), 731-739. DOI: 10.1016/S0140-6736(06)68231-7.
(2) Duthie, E. S.; Lorenz, L. L. Staphylococcal coagulase; mode of action and antigenicity. *J Gen Microbiol* **1952**, *6* (1-2), 95-107. DOI: 10.1099/00221287-6-1-2-95 From NLM Medline.
(3) Grundling, A.; Schneewind, O. Cross-linked peptidoglycan mediates lysostaphin binding to the cell wall envelope of Staphylococcus aureus. *J Bacteriol* **2006**, *188* (7), 2463-2472. DOI: 10.1128/Jb.188.7.2463-2472.2006.
(4) Baba, T.; Takeuchi, F.; Kuroda, M.; Yuzawa, H.; Aoki, K.; Oguchi, A.; Nagai, Y.; Iwama, N.; Asano, K.; Naimi, T.; et al. Genome and virulence determinants of high virulence community-acquired MRSA. *Lancet* **2002**, *359* (9320), 1819-1827. DOI: Doi 10.1016/S0140-6736(02)08713-5.
(5) Horsburgh, M. J.; Aish, J. L.; White, I. J.; Shaw, L.; Lithgow, J. K.; Foster, S. J. sigmaB modulates virulence determinant expression and stress resistance: characterization of a functional rsbU strain derived from Staphylococcus aureus 8325-4. *J Bacteriol* **2002**, *184* (19), 5457-5467. DOI: 10.1128/JB.184.19.5457-5467.2002 From NLM Medline.
(6) Holden, M. T.; Feil, E. J.; Lindsay, J. A.; Peacock, S. J.; Day, N. P.; Enright, M. C.; Foster, T. J.; Moore, C. E.; Hurst, L.; Atkin, R.; et al. Complete genomes of two clinical Staphylococcus aureus strains: evidence for the rapid evolution of virulence and drug resistance. *Proc Natl Acad Sci U S A* **2004**, *101* (26), 9786-9791. DOI: 10.1073/pnas.0402521101 From NLM Medline.
(7) Holden, M. T. G.; Lindsay, J. A.; Corton, C.; Quail, M. A.; Cockfield, J. D.; Pathak, S.; Batra, R.; Parkhill, J.; Bentley, S. D.; Edgeworth, J. D. Genome Sequence of a Recently Emerged, Highly Transmissible, Multi-Antibiotic- and Antiseptic-Resistant Variant of Methicillin-Resistant Staphylococcus aureus, Sequence Type 239 (TW). *J Bacteriol* **2010**, *192* (3), 888-892. DOI: 10.1128/Jb.01255-09.
(8) Hiramatsu, K.; Aritaka, N.; Hanaki, H.; Kawasaki, S.; Hosoda, Y.; Hori, S.; Fukuchi, Y.; Kobayashi, I. Dissemination in Japanese hospitals of strains of Staphylococcus aureus heterogeneously resistant to vancomycin. *Lancet* **1997**, *350* (9092), 1670-1673. DOI: 10.1016/S0140-6736(97)07324-8 From NLM Medline.
(9) Richardson, J. F.; Reith, S. Characterization of a strain of methicillin-resistant Staphylococcus aureus (EMRSA-15) by conventional and molecular methods. *J Hosp Infect* **1993**, *25* (1), 45-52. DOI: 10.1016/0195-6701(93)90007-m From NLM Medline.
(10) Novick, R. Properties of a cryptic high-frequency transducing phage in Staphylococcus aureus. *Virology* **1967**, *33* (1), 155-166. DOI: 10.1016/0042-6822(67)90105-5 From NLM Medline. Baek, K. T.; Frees, D.; Renzoni, A.; Barras, C.; Rodriguez, N.; Manzano, C.; Kelley, W. L. Genetic variation in the Staphylococcus aureus 8325 strain lineage revealed by whole-genome sequencing. *PLoS One* **2013**, *8* (9), e77122. DOI: 10.1371/journal.pone.0077122 From NLM Medline.
(11) Frees, D.; Qazi, S. N. A.; Hill, P. J.; Ingmer, H. Alternative roles of ClpX and ClpP in Staphylococcus aureus stress tolerance and virulence. *Mol Microbiol* **2003**, *48* (6), 1565-1578. DOI: 10.1046/j.1365-2958.2003.03524.x.
(12) Baek, K. T.; Bowman, L.; Millership, C.; Dupont Sogaard, M.; Kaever, V.; Siljamaki, P.; Savijoki, K.; Varmanen, P.; Nyman, T. A.; Grundling, A.; et al. The Cell Wall Polymer

Lipoteichoic Acid Becomes Nonessential in Staphylococcus aureus Cells Lacking the ClpX Chaperone. *mBio* **2016**, *7* (4). DOI: 10.1128/mBio.01228-16 From NLM Medline.
(13) Somerville, G. A.; Beres, S. B.; Fitzgerald, J. R.; DeLeo, F. R.; Cole, R. L.; Hoff, J. S.; Musser, J. M. In vitro serial passage of Staphylococcus aureus: Changes in physiology, virulence factor production, and agr nucleotide sequence. *J Bacteriol* **2002**, *184* (5), 1430-1437. DOI: 10.1128/Jb.184.5.1430-1437.2002.
(14) Jelsbak, L.; Ingmer, H.; Valihrach, L.; Cohn, M. T.; Christiansen, M. H. G.; Kallipolitis, B. H.; Frees, D. The Chaperone ClpX Stimulates Expression of Staphylococcus aureus Protein A by Rot Dependent and Independent Pathways. *Plos One* **2010**, *5* (9). DOI: ARTN e12752 10.1371/journal.pone.0012752.
(15) Stahlhut, S. G.; Alqarzaee, A. A.; Jensen, C.; Fisker, N. S.; Pereira, A. R.; Pinho, M. G.; Thomas, V. C.; Frees, D. The ClpXP protease is dispensable for degradation of unfolded proteins in Staphylococcus aureus. *Sci Rep-Uk* **2017**, *7*. DOI: ARTN 11739 10.1038/s41598-017-12122-y.
(16) Oku, Y.; Kurokawa, K.; Matsuo, M.; Yamada, S.; Lee, B. L.; Sekimizu, K. Pleiotropic Roles of Polyglycerolphosphate Synthase of Lipoteichoic Acid in Growth of Staphylococcus aureus Cells. *J Bacteriol* **2009**, *191* (1), 141-151. DOI: 10.1128/Jb.01221-08.
(17) Omae, Y.; Hanada, Y.; Sekimizu, K.; Kaito, C. Silkworm apolipophorin protein inhibits hemolysin gene expression of Staphylococcus aureus via binding to cell surface lipoteichoic acids. *J Biol Chem* **2013**, *288* (35), 25542-25550. DOI: 10.1074/jbc.M113.495051 From NLM Medline.
(18) Christensen, G. D.; Bisno, A. L.; Parisi, J. T.; Mclaughlin, B.; Hester, M. G.; Luther, R. W. Nosocomial Septicemia Due to Multiply Antibiotic-Resistant Staphylococcus-Epidermidis. *Ann Intern Med* **1982**, *96* (1), 1-10. DOI: 10.7326/0003-4819-96-1-1.
(19) Meric, G.; Mageiros, L.; Pensar, J.; Laabei, M.; Yahara, K.; Pascoe, B.; Kittiwan, N.; Tadee, P.; Post, V.; Lamble, S.; et al. Disease-associated genotypes of the commensal skin bacterium Staphylococcus epidermidis. *Nat Commun* **2018**, *9* (1), 5034. DOI: 10.1038/s41467-018-07368-7 From NLM Medline.
(20) Yoshikawa, H.; O'Sullivan, A.; Sueoka, N. Sequential Replication of the Bacillus Subtilis Chromosome. 3. Regulation of Initiation. *Proc Natl Acad Sci U S A* **1964**, *52* (4), 973-980. DOI: 10.1073/pnas.52.4.973 From NLM Medline.
(21) Jacob, A. E.; Hobbs, S. J. Conjugal Transfer of Plasmid-Borne Multiple Antibiotic-Resistance in Streptococcus-Faecalis Var Zymogenes. *J Bacteriol* **1974**, *117* (2), 360-372. DOI: Doi 10.1128/Jb.117.2.360-372.1974.
(22) Rice, L. B.; Carias, L.; Rudin, S.; Vael, C.; Goossens, H.; Konstabel, C.; Klare, I.; Nallapareddy, S. R.; Huang, W.; Murray, B. E. A potential virulence gene, hylEfm, predominates in Enterococcus faecium of clinical origin. *J Infect Dis* **2003**, *187* (3), 508-512. DOI: 10.1086/367711 From NLM Medline.
(23) Kuypers, J. M.; Heggen, L. M.; Rubens, C. E. Molecular Analysis of a Region of the Group-B Streptococcus Chromosome Involved in Type-Iii Capsule Expression. *Infect Immun* **1989**, *57* (10), 3058-3065. DOI: Doi 10.1128/Iai.57.10.3058-3065.1989.
(24) Blattner, F. R.; Plunkett, G.; Bloch, C. A.; Perna, N. T.; Burland, V.; Riley, M.; ColladoVides, J.; Glasner, J. D.; Rode, C. K.; Mayhew, G. F.; et al. The complete genome sequence of Escherichia coli K-12. *Science* **1997**, *277* (5331), 1453-+. DOI: DOI 10.1126/science.277.5331.1453.
(25) Stover, C. K.; Pham, X. Q.; Erwin, A. L.; Mizoguchi, S. D.; Warrener, P.; Hickey, M. J.; Brinkman, F. S. L.; Hufnagle, W. O.; Kowalik, D. J.; Lagrou, M.; et al. Complete genome

sequence of Pseudomonas aeruginosa PAO1, an opportunistic pathogen. *Nature* **2000**, *406* (6799), 959-964. DOI: Doi 10.1038/35023079.
(26) Thorpe, H. A.; Booton, R.; Kallonen, T.; Gibbon, M. J.; Couto, N.; Passet, V.; Loez-Fernadez, S.; Rodrigues, C.; Matthews, L.; Mitchell, S.; et al. A large-scale genomic snapshot of Klebsiella spp. isolates in Northern Italy reveals limited transmission between clinical and non-clinical settings. *Nat Microbiol* **2022**, *7* (12), 2054-+. DOI: 10.1038/s41564-022-01263-0.
(27) Magda, M.; Bettoni, S.; Laabei, M.; Fairley, D.; Russo, T. A.; Riesbeck, K.; Blom, A. M. Clinical Isolates of Acinetobacter spp. Are Highly Serum Resistant Despite Efficient Recognition by the Complement System. *Front Immunol* **2022**, *13*, 814193. DOI: 10.3389/fimmu.2022.814193 From NLM Medline.
